# Expedient Synthesis of Substituted Thieno[3,2-*b*]thiophenes and Selenopheno[3,2-*b*]selenophenes Through Cascade Cyclization of Alkynyl Diol Derivatives

**DOI:** 10.3390/molecules29235507

**Published:** 2024-11-21

**Authors:** Yingqi Feng, Xuelin Zhang, Ziqing He, Miaoshan Zhao, Lu Chen, Yibiao Li, Xiai Luo

**Affiliations:** 1 Jiangmen Key Laboratory of Synthetic Chemistry and Cleaner Production, School of Environmental & Chemical Engineering, Wuyi University, Jiangmen 529020, China; wyuchemfyq@126.com (Y.F.); wyuchemzxl@126.com (X.Z.); wyuchemhzq@126.com (Z.H.); wyuchemzms@126.com (M.Z.); wyuchemcl@126.com (L.C.); 2 Hunan Province Key Laboratory for Synthetic Biology of Traditional Chinese Medicine, School of Pharmaceutical Sciences, Hunan University of Medicine, Huaihua 418000, China

**Keywords:** thienothiophene, cyclization, sulfur radical, step-efficiency, dehydration

## Abstract

Thieno[3,2-*b*]thiophenes are used as key components in optoelectronic materials, porous hydrogen-storage hosts, organic solar cells, and polymer semiconductors. A step-efficient synthetic protocol was proposed herein for obtaining multisubstituted thieno[3,2-*b*]thiophene and selenopheno[3,2-*b*]selenophenes in moderate to good yields via the bisulfur/biselenium cyclization of alkynyl diols with I_2_/Na_2_S_2_O_3_ or selenium. Using this strategy, substitution patterns were obtained for backbone modification in functional materials.

## 1. Introduction

Efficient, sustainable methods for thienothiophene (TT) synthesis have been increasingly developed because these compounds are crucial in the fields of fine chemicals, biodiagnostics, and electronic and optoelectronic devices. TTs have four regional isomers based on the position of two sulfur atoms: thieno[3,2-*b*]thiophene, thieno[2,3-*b*]thiophene, thieno[3,4-*b*]thiophene, and thieno[3,4-*c*]thiophene [1,2,3,4]. Among these, thieno[3,2-*b*]thiophene derivatives have garnered considerable interest due to their well-known optoelectronic applications. These are also widely used in organic solar cells, organic field-effect transistors, photovoltaic devices [5,6,7,8], and as porous hydrogen-storage hosts and polymer semiconductors [9,10,11]. However, methods to efficiently construct the core nucleus of this derivative are highly limited, thereby hampering its further research and applications in the material chemistry and fine chemical industries. Therefore, the step-efficiency synthesis of thieno[3,2-*b*]thiophenes via the direct use of simple, commercial raw materials has gained popularity.

**Scheme 1:**
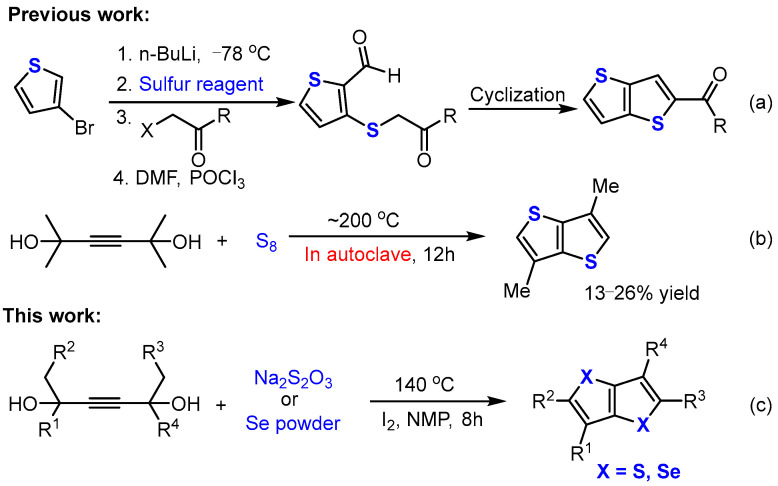
Previous methods for synthesis of thieno[3,2-*b*]thiophene (**a**,**b**) and sulfur cyclization hypothesis (**c**).

The majority of synthesis strategies for thieno[3,2-*b*]thiophenes involve multistep synthesis such as nucleophilic substitution and the cyclization reaction. Moreover, 3-bromothiophenes are used as starting materials in these reactions, which usually yield low synthesis efficiency and overall yield (Scheme 1a) [12,13,14,15]. For instance, Rutherford et al. proposed a typical method for thieno[3,2-*b*]thiophene synthesis where 3-bromothiophene was used as a starting material and subsequently reacted with n-BuLi, sulfur powder, BrCH_2_COOMe, POCl_3_, MeONa, and LiOH to obtain the desired products via four-step synthesis. However, more reaction steps were required to synthesize its substituted derivatives [16]. Moreover, multistep synthesis and many additional chemical reagents are required in such methods, thereby hindering the production efficiency and green chemistry requirements. To overcome these limitations, the step-efficiency synthesis method was proposed to produce thieno[3,2-*b*]thiophenes via the tandem bisulfide cyclization of unsaturated hydrocarbons. To our knowledge, Lozac’h et al. was the first to directly transform alkynyl diols into thieno[3,2-*b*]thiophenes in 1955 [17,18]. They synthesized only small amounts of thieno[3,2-*b*]thiophenes using elemental sulfur as a sulfurizing agent; the reaction proceeded in an autoclave and a high temperature of 200 °C (Scheme 1b). Although this method provided very low yields, it is still used to synthesize functional materials due to the advantages of step efficiency [19,20,21]. The trisulfur radical anion (S3^•−^) is an efficient sulfurization reagent for synthesizing thiophenes via the interaction between sulfur/base [22,23,24,25], Na_2_S·9H_2_O [26,27], and K_2_S [28,29]; an alkaline environment is usually required to obtain S3^•−^. User-friendly sulfinates are also used as efficient sulfinyl or sulfonyl radicals for radical addition on unsaturated hydrocarbons [30,31,32,33] and C–H/C–X sulfenylation [34,35,36], which better complement S_3_^•−^ radicals. The Na_2_S_2_O_3_ is generally used to generate acyl-Bunte salts or thiol intermediates [37,38,39]; however, its use in synthesizing sulfur-containing heterocycles has rarely been reported. Based on the sulfur cyclization reaction in our previous work [40,41,42,43], a facile approach was reported herein for producing thieno[3,2-*b*]thiophenes and selenopheno[3,2-*b*]selenophenes using Na_2_S_2_O_3_ and Se powder as the efficiency sulfurizing and selenating reagent in good yields (Scheme 1c).

## 2. Results and Discussion

2,5-Dimethylhex-3-yne-2,5-diol **1a** and various sulfur sources were used as the model substrates to optimize the reaction conditions to evaluate this bisulfur cyclization hypothesis (Table 1). A series of experiments was conducted to determine the optimal sulfur sources, solvents, additives, and reaction temperature and time. First, bisulfur cyclization proceeded at a reaction temperature of 150 °C to afford 3,6-dimethylthieno[3,2-*b*]thiophene **2a** in a 21% yield (Table 1, entry 1), consistent with previous reports [17,18]. Then, a series of sulfur sources was tested including S_8_, potassium ethylxanthate (EtOCS_2_K), thiourea, K_2_S, Na_2_S·9H_2_O, and Na_2_S_2_O_3_ (Table 1, entries 1–6). The reaction proceeded most efficiently when Na_2_S_2_O_3_ was used as the sulfur reagent, affording the target product **2a** in a 31% yield (Table 1, entry 6). However, the reaction yield was not improved by further increasing the number of equivalents of Na_2_S_2_O_3_ (Table 1, entry 7). Surprisingly, when I_2_ (1 equiv.) was used as an additive, the reaction yield improved considerably (Table 1, entries 8–9). The remaining additive NH_4_I did not effectively promote bisulfur cyclization (Table 1, entry 10). The results indicated that protonic acids, such as HCl, also promoted bisulfur cyclization and afforded **2a** in a 57% yield (Table 1, entry 11). Notably, the yield of **2a** decreased to 61% on reducing the amount of I_2_ (entry 12); in contrast, when the amount of I_2_ was increased, the yield increased only slightly (entry 13). Using Na_2_S_2_O_3_ as the sulfur reagent and NMP as the solvent afforded **2a** in high yields, making them ideal for bisulfur cyclization. The other solvents—DMSO, DMF, DMAc, and CH_3_COOH—reduced the yield of **2a** (Table 1, entries 14–17). The screening of various reaction temperatures revealed that it was crucial for bisulfur cyclization (Table 1, entries 18–19). The yield of **2a** decreased slightly when the reaction time was reduced or extended (Table 1, entries 20–21). When the oxidant DDQ was added, the yield of the reaction did not change significantly (Table 1, entry 22). Thus, the optimized reaction conditions were **1a** (0.5 mmol), Na_2_S_2_O_3_ (1.0 mmol), and I_2_ (0.5 mmol) in 2.0 mL of NMP at 140 °C for 8 h (Table 1, entry 20).

**Scheme 2:**
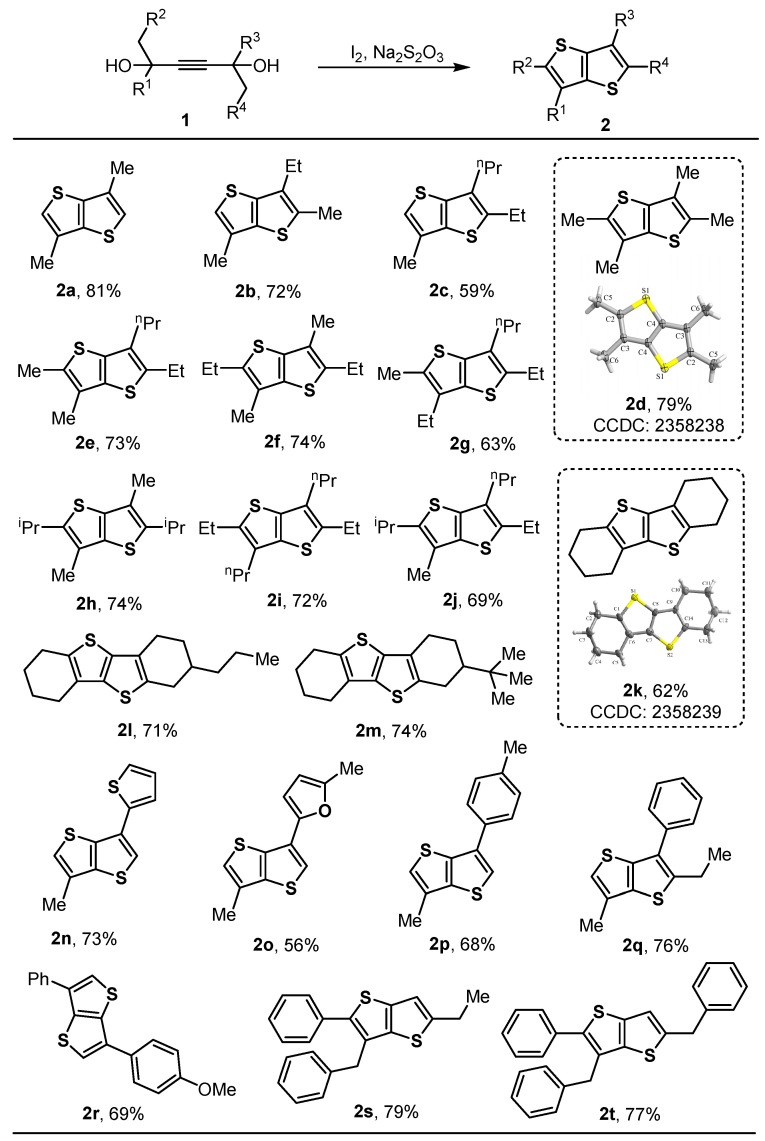
Substrate scope of alkynyl diol derivatives. Reaction condition: alkynyl diol **1** (0.5 mmol), Na_2_S_2_O_3_ (1.0 mmol), I_2_ (0.5 mmol), and NMP (2 mL) stirred at 140 °C for 8 h in a sealed tube under an air atmosphere. Isolated yields.

Using these optimized reaction conditions (Table 1, entry 20), the versatility of the proposed method for the bisulfur cyclization of various alkynyl diols was validated. The corresponding results are shown in Scheme 2. First, dialkyl alkynyl diols (i.e., **1a**, 1-phenyldodec-4-yn-6-ol (**1b**), and 2-methyl-5-propyloct-3-yne-2,5-diol (**1c**)) yielded their respective thieno[3,2-*b*]thiophene products in 59–81% yields (Scheme 2, **2a**–**2c**). These dialkyl alkynyl diols were not derived from isomerization during dehydration and bisulfur cyclization because they follow Zaitsev’s rule for hydroxyl elimination [44]. Subsequently, 2,3,4,5-tetramethyl substituted thieno[3,2-*b*]thiophene (**2d**) was obtained in a 79% yield after 8 h. Single-crystal X-ray diffraction analysis was performed to verify the structure of **2d** (Scheme 2; CCDC: 2358238). Then, a series of tetrasubstituted thieno[3,2-*b*]thiophene derivatives with different alkyl combinations was efficiently obtained in moderate yields using high chemo and regioselectivity modes (Scheme 2, **2e**–**2j**). The reactivity of different alkynyl diols containing a cyclic substituent on the alkyl side chains was also investigated. Various cyclohexane substituents were well-tolerated in the reaction, affording thieno[3,2-*b*]thiophenes in moderate yields (Scheme 2, **2k**–**2m**). The molecular structure of thieno[3,2-*b*]thiophene **2k** was verified via single-crystal X-ray diffraction (CCDC: 2358239). Alkynyl diols with heterocycle substituents on the R3 groups also successfully underwent sulfur cyclization, affording thiophene-substituted thieno[3,2-*b*]thiophene (**2n**) and furan-substituted thieno[3,2-*b*]thiophene (**2o**) in moderate yields. Subsequently, additional classes of aryl-substituted alkynyl diols were used as starting materials to obtain aryl-substituted thieno[3,2-*b*]thiophenes. The reaction of aryl-substituted alkynyl diols with sulfur powder afforded acetophenone as the major product, with only thieno[3,2-*b*]thiophenes in a 3% yield [18]. When the electron-rich aromatic side chains (R^3^ or R^1^ group) linked to the hydroxyl group were reacted with I_2_/Na_2_S_2_O_3_, the corresponding thieno[3,2-*b*]thiophene products were obtained in 69%yields (Scheme 2, **2p**–**2r**). This reaction tolerated various substituents including –Me and –OMe groups. Bisulfur cyclization also proceeded smoothly when the benzyl substituent was linked to the hydroxyl group, affording triphenyl substituted thieno[3,2-*b*]thiophenes **2s** and **2t** in 79% and 77% yields, respectively.

**Scheme 3:**
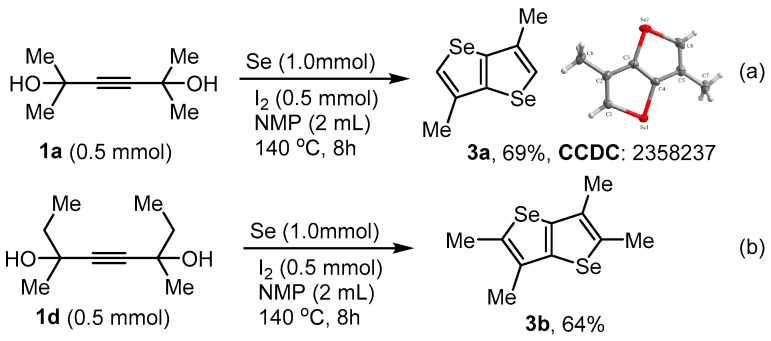
Synthesis of selenopheno[3,2-*b*]selenophenes. (**a**) Synthesis of 3,6-dimethylselenopheno[3,2-*b*]selenophene **3a**. (**b**) Synthesi of 2,3,5,6-tetramethylselenopheno[3,2-*b*]selenophene **3b**.

Alkynyl diols **1a** and **1d** were used as starting materials to synthesize selenopheno[3,2-*b*]selenophene derivatives. Using Se powder as the “Se” source, selenopheno[3,2-*b*]selenophenes were afforded under similar reaction conditions. When 2,5-dimethylhex-3-yne-2,5-diol and ethyloct-4-yne-3,6-diol were used as starting materials, selenium cyclization was successfully completed to obtain the corresponding 3,6-disubstituted and 2,3,4,5-tetrasubstituted selenopheno[3,2-*b*]selenophene **3a** and **3b** in 69% and 64% yields, respectively (Scheme 3). To confirm the chemical structure of selenopheno[3,2-*b*]selenophene **3a**, the product was crystallized and analyzed via single-crystal X-ray diffraction (CCDC: 2358237).

**Scheme 4:**
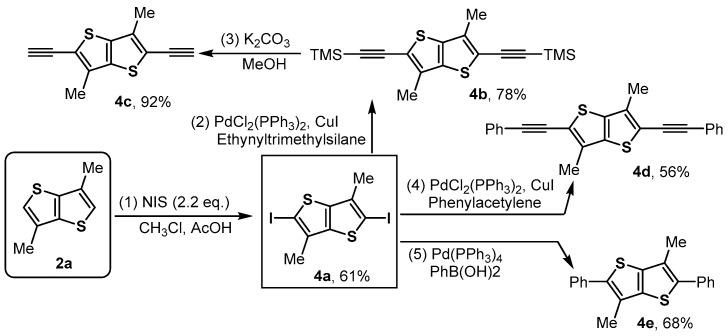
Synthetic modifications of thienothiophene **2a**.

As an important building block, the newly formed thieno[3,2-*b*]thiophene **2a** was used in various synthetic transformations (Scheme 4). The iodination of thieno[3,2-*b*]thiophene **2a** afforded 2,5-diiodo-3,6-dimethylthieno[3,2-*b*]thiophene **4a**, which could be effectively transformed in transition metal-catalyzed coupling reactions. For instance, 2,5-diiodo-3,6-dimethylthieno[3,2-*b*]thiophene **4a** smoothly reacted with terminal alkynes via Sonogashira coupling to afford **4b** and **4d** in 78% and 56% yields, respectively. Then, acetylene-substituted thieno[3,2-*b*]thiophene (**4c**) was obtained via a desilylation of thieno[3,2-*b*]thiophene **4b** in a 92% yield; **4b** is an important organic synthon that can be used in optoelectronic materials. The Pd-catalyzed Suzuki–Miyaura coupling reaction between **4a** and phenylboronic acid afforded 3,6-dimethyl-2,5-diphenylthieno[3,2-*b*]thiophene **4e** in a 68% yield. These products can be converted into oligothiophenes that are potentially valuable in optoelectronic materials [45,46].

**Scheme 5:**
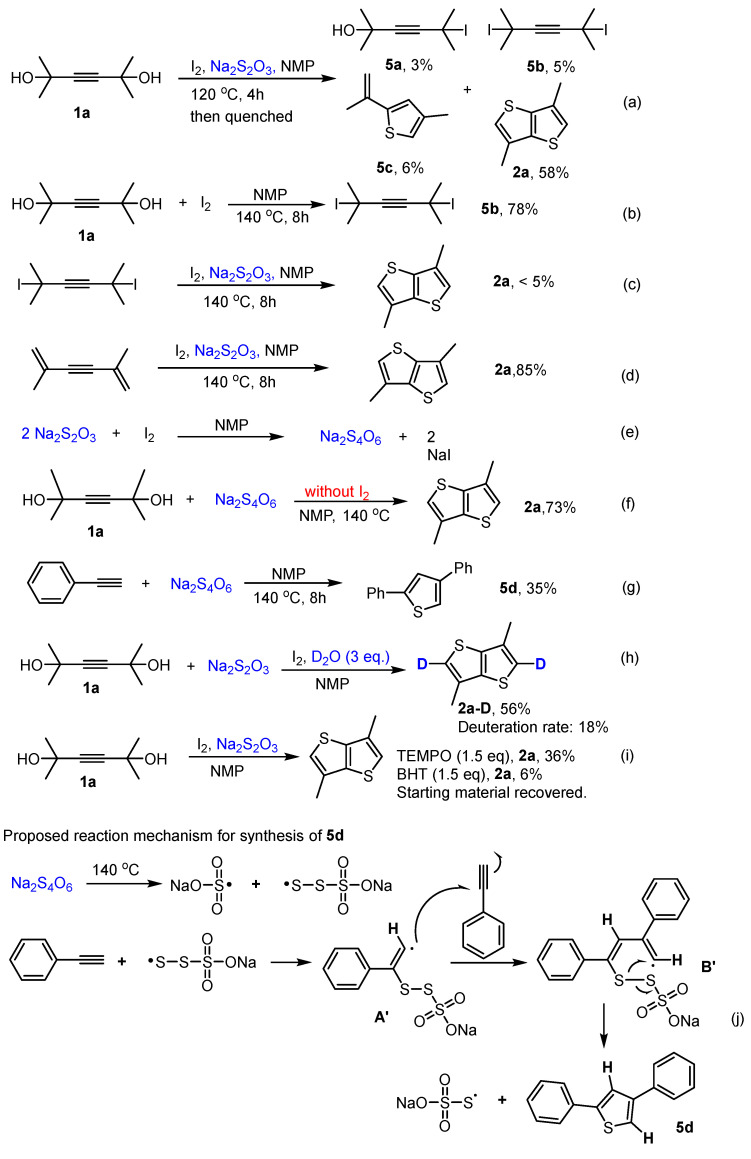
Control experiments. (**a**) Intermediate detection reaction. (**b**) Iodination of 2,5-dimethylhex-3-yne-2,5-diol. (**c**) Sulfur cyclization of 1,4-diiodobut-2-yne. (**d**) Sulfur cyclization of 2,5-dimethylhexa-1,5-dien-3-yne. (**e**) Redox reaction of Na_2_S_2_O_3_ with I_2._ (**f**) Na_2_S_4_O_6_ reaction with 2,5-dimethylhex-3-yne-2,5-diol give thieno[3,2-*b*]thiophene. (**g**) Sulfur cyclization of phenylacetylene with Na_2_S_4_O_6._ (**h**) Deuteration experiments. (**i**) Radical inhibition reaction. (**j**) Proposed reaction mechanism for synthesis of **5d**.

To investigate the sulfur cyclization mechanism for synthesizing thieno[3,2-*b*]thiophenes, several control experiments were conducted (Scheme 5). The reaction temperature and time were initially reduced, which yielded iodine alkyne **5a**, **5b**, thiophene **5c**, and thieno[3,2-*b*]thiophene **2a** (Scheme 5a). Then, in the absence of Na_2_S_2_O_3_, 2,5-dimethylhex-3-yne-2,5-diol **1a** and I_2_ underwent iodination at 140 °C to afford 1,4-diiodobut-2-yne **5b** in a 78% yield (Scheme 5b). Unfortunately, 1,4-diiodobut-2-yne **5b** did not yield **2a** under standard reaction conditions, indicating that **5b** is not a reaction intermediate (Scheme 5c). The expected product **2a** was formed when using 2,5-dimethylhexa-1,5-dien-3-yne as the substrate, suggesting that alkynyldiene may be an intermediate in the reaction (Scheme 5d). Na_2_S_4_O_6_ can be rapidly produced via the redox reaction of Na_2_S_2_O_3_ with I_2_ under neutral or weakly acidic conditions, as opposed to the iodination of alkynyl diol **1a** (Scheme 5e). As Na_2_S_2_O_3_ readily underwent the redox reaction with I_2_ to afford sodium tetrathionate (Na_2_S_4_O_6_), Na_2_S_4_O_6_ was used in the absence of I_2_ to obtain thieno[3,2-*b*]thiophene **2a** in a 73% yield (Scheme 5f). This confirms that Na_2_S_4_O_6_ is a key intermediate for generating a reactive sulfur reagent for bisulfur cyclization. To further verify this result, phenylacetylene was reacted with Na_2_S_4_O_6_ under a transition-metal catalysis free condition; the reaction afforded 2,4-diphenylthiophene **5d** in a 35% yield, indicating that the process can be completed by the tandem sulfur radical addition to alkynes (Scheme 5g) [47]. The plausible mechanism for the synthesis of **5d** is shown in Scheme 5j. Although sulfur cyclization is not an H-abstraction process, deuteration experiments suggested that hydrogen–deuterium exchange occurred during the reaction (Scheme 5h). Nevertheless, this is more likely due to the direct hydrogen-deuterium exchange reaction of the 2-position C(sp^2^)-H bond of thieno[3,2-*b*]thiophenes [48,49]. Furthermore, when radical scavengers such as butylated hydroxytoluene (BHT) and 2,2,6,6-tetramethylpiperidin-1-yl)oxidanyl (TEMPO) were added, dehydration and cyclization were subdued, and most of the raw materials were recycled. This indicates that sulfur cyclization may proceed via a radical pathway (Scheme 5i).

**Scheme 6:**
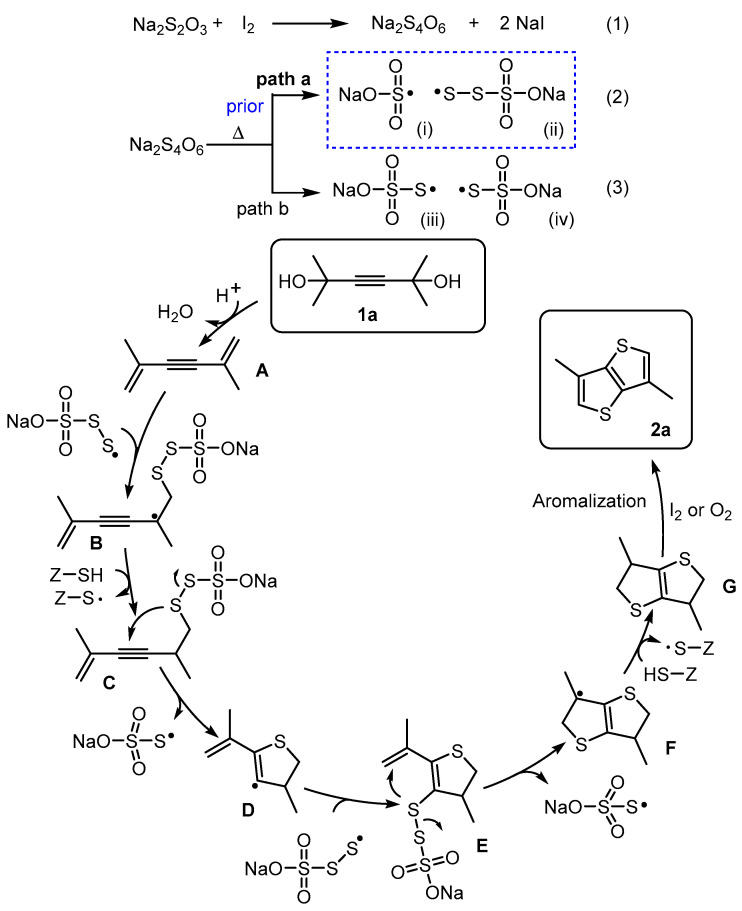
Proposed reaction mechanism.

Based on these control experiments and previous reports, a plausible mechanism was developed (Scheme 6). First, Na_2_S_2_O_3_ reacted with I_2_ to afford Na_2_S_4_O_6_, which may thermally decompose into reactive sulfur radicals (i–iv) under high-temperature conditions. As the breaking and bonding of sulfur radicals are reversible, the resulting sulfur radical must be sufficiently stable to be captured by the unsaturated bond. Moreover, the sulfite radical anion (i) is relatively stable among the four sulfur radicals reported herein [50,51,52,53,54]. Therefore, i and perthiyl radical (ii) can be obtained via the heterolytic cleavage of Na_2_S_4_O_6_ according to the reaction pathway (Scheme 6, Equation (2)). The dehydrogenation process leads to alkynyldiene intermediate **A**. Then, the more reactive perthiyl radical (RSS•) was added to C=C double bonds of alkynyldiene **A**, which gave the intermediate **B** [55,56,57]. Intermediate **B** captured a proton from Z-SH or solvents to yield the intermediate **C**, which subsequently generated a sulfur radical via S–S bond homolysis and released NaO_3_S_2_•. In this process, the Z-SH represents any thiol in the medium (sulfide or H_2_S, etc.). This sulfur radical was intramolecularly added to the C≡C triple bond formation of intermediate **D**, which immediately underwent radical coupling with the perthiyl radical (ii) to afford intermediate **E**. This intermediate then underwent a second intramolecular radical cyclization to yield the alkenyl sulfide intermediate **F** and released NaO_3_S_2_•. Finally, thieno[3,2-*b*]thiophene **2a** was produced via sequential H-abstraction and further oxidation aromatization of the intermediate **G** [28,58,59,60]. It should be noted that although the reaction requires the removal of two molecules of H_2_O, it is possible that the dehydration process is not simultaneous.

## 3. Materials and Methods

### 3.1. General Methods (Chemistry)

The general methods are described in the Supplementary Materials.

### 3.2. General Procedures for the Preparation of Compounds ***2a***–***2t***

A mixture of 2,5-dimethylhex-3-yne-2,5-diol (71 mg, 0.5 mmol), Na_2_S_2_O_3_ (158 mg, 1.0 mmol), I_2_ (127 mg, 0.5 mmol), and NMP (2 mL) was added successively into a 20 mL Schlenk tube. The Schlenk tube was then immersed in an oil bath at 140 °C with stirring for 8 h. After cooling down to room temperature, the reaction was quenched with 10 mL of brine and extracted with ethyl acetate (2 × 5 mL). The combined organic layer was dried over Na_2_SO_4_, filtered, and concentrated under vacuum. The residue was purified by flash chromatography (Hexane, R*_f_* = 0.7) on silica gel, which furnished product **2a** (68 mg, 81% yield) as a pale yellow solid. The following compounds **2b**–**2v** were prepared by a similar method, unless otherwise noted.

*3,6-Dimethylthieno[3,2-b]thiophene* (**2a**), Yellow solid (68 mg, 81% yield); R*_f_* = 0.7 (Hexane); ^1^H NMR (500 MHz, CDCl_3_) δ 6.96 (s, 2H), 2.36 (s, 6H). ^13^C NMR (125 MHz, CDCl_3_) δ 140.0 (2C), 130.3 (2C), 121.8 (2C), 14.6 (2C). ESI-HRMS (*m*/*z*) [M + H]^+^ calcd. for C_8_H_9_S_2_^+^, 169.0140, found: 169.0138.
*3-Ethyl-2,6-dimethylthieno[3,2-b]thiophene* (**2b**), Yellow liquid (71 mg, 72% yield); R*_f_* = 0.7 (Hexane); ^1^H NMR (400 MHz, CDCl_3_) δ 6.87 (s, 1H), 2.69 (q, *J* = 7.6 Hz, 2H), 2.48 (s, 3H), 2.34 (s, 3H), 1.27 (t, *J* = 7.6 Hz, 3H). ^13^C NMR (100 MHz, CDCl_3_) δ 139.3, 136.1, 133.9, 132.2, 129.9, 119.7, 20.9, 14.7, 14.1, 13.4. ESI-HRMS (*m*/*z*): [M]^+^ calcd. for C_10_H_12_S_2_^+^, 196.0374, found: 196.0368.
*2-Ethyl-6-methyl-3-propylthieno[3,2-b]thiophene* (**2c**), Brown liquid (66 mg, 59% yield); R*_f_* = 0.7 (Hexane); ^1^H NMR (400 MHz, CDCl_3_) δ 6.87 (s, 1H), 2.86 (q, *J* = 7.5 Hz, 2H), 2.70–2.62 (m, 2H), 2.34 (s, 3H), 1.82–1.66 (m, 2H), 1.32 (t, *J* = 7.5 Hz, 3H), 0.98 (t, *J* = 7.4 Hz, 3H). ^13^C NMR (100 MHz, CDCl_3_) δ 142.5, 130.0, 129.9, 119.1, 29.6, 22.4, 22.3, 16.4, 14.7, 14.0. ESI-HRMS (*m*/*z*) [M]^+^ calcd. for C_12_H_16_S_2_^+^, 224.0688, found: 224.0679.
*2,3,5,6-Tetramethylthieno[3,2-b]thiophene* (**2d**), Yellow solid (77 mg, 79% yield); R*_f_* = 0.7 (Hexane); MP: 136–137 °C. ^1^H NMR (500 MHz, CDCl_3_) *δ* 2.45 (s, 6H), 2.21 (s, 6H). ^13^C NMR (125 MHz, CDCl_3_) *δ* 136.1 (2C), 132.0 (2C), 125.3 (2C), 14.0 (2C), 12.5 (2C). ESI-HRMS (*m*/*z*) [M + K]^+^ calcd. for C_10_H_12_KS_2_^+^, 235.0012, found: 235.0022.
*2-Ethyl-5,6-dimethyl-3-propylthieno[3,2-b]thiophene* (**2e**), Brown liquid (87 mg, 73% yield); R*_f_* = 0.7 (Hexane); ^1^H NMR (400 MHz, CDCl_3_) *δ* 2.83 (q, *J* = 7.5 Hz, 2H), 2.62–2.59 (m, 2H), 2.43 (s, 3H), 2.20 (s, 3H), 1.70 (q, *J* = 7.5 Hz, 2H), 1.30 (t, *J* = 7.5 Hz, 3H), 0.96 (t, *J* = 7.3 Hz, 3H). ^13^C NMR (100 MHz, CDCl_3_) *δ* 140.3, 136.7, 135.1, 132.2, 129.5, 125.38, 29.7, 22.3, 22.2, 16.5, 14.0, 14.0, 12.5. ESI-HRMS (*m*/*z*) [M + H]^+^ calcd. for C_13_H_19_S_2_^+^, 239.0923, found: 239.0921.
*2,5-Diethyl-3,6-dimethylthieno[3,2-b]thiophene* (**2f**), Yellow solid (83 mg, 74% yield); R*_f_* = 0.7 (Hexane); MP: 79–81 °C. ^1^H NMR (500 MHz, CDCl_3_) *δ* 2.83 (q, *J* = 7.5 Hz, 4H), 2.23 (s, 6H), 1.30 (t, *J* = 7.6 Hz, 6H). ^13^C NMR (125 MHz, CDCl_3_) *δ* 139.9 (2C), 136.2 (2C), 124.5 (2C), 22.3 (2C), 16.0 (2C), 12.4 (2C). ESI-HRMS (*m*/*z*) [M + H]^+^ calcd. for C_12_H_17_S_2_^+^, 225.0766, found: 225.0766.
*2,6-Diethyl-5-methyl-3-propylthieno[3,2-b]thiophene* (**2g**), Brown liquid (79 mg, 63% yield); R*_f_* = 0.7 (Hexane); ^1^H NMR (400 MHz, CDCl_3_) *δ* 2.83 (q, *J* = 7.5 Hz, 2H), 2.68–2.58 (m, 4H), 2.44 (s, 3H), 1.75–1.63 (m, 2H), 1.31–1.23 (m, 6H), 0.96 (t, *J* = 7.4 Hz, 3H). ^13^C NMR (100 MHz, CDCl_3_) *δ* 140.3, 135.7, 135.5, 131.8, 131.7, 129.4, 29.7, 22.3, 22.1, 20.9, 16.4, 14.0, 13.8, 13.4. ESI-HRMS (*m*/*z*) [M + H]^+^ calcd. for C_14_H_21_S_2_^+^, 253.1079, found: 253.1077.
*2,5-Diisopropyl-3,6-dimethylthieno[3,2-b]thiophene* (**2h**), Yellow solid (93 mg, 74% yield); R*_f_* = 0.7 (Hexane); MP: 141–142 °C. ^1^H NMR (500 MHz, CDCl_3_) *δ* 3.35–3.29 (m, 2H), 2.25 (s, 3H), 1.33 (s, 6H), 1.32 (s, 6H). ^13^C NMR (125 MHz, CDCl_3_) *δ* 146.1 (2C), 135.9 (2C), 123.7 (2C), 28.9 (2C), 24.5 (4C), 12.6 (2C). ESI-HRMS (*m*/*z*) [M + H]^+^ calcd. for C_14_H_21_S_2_^+^, 253.1079, found: 253.1077.
*2,5-Diethyl-3,6-dipropylthieno[3,2-b]thiophene* (**2i**), Brown liquid (101 mg, 72% yield); R*_f_* = 0.7 (Hexane); ^1^H NMR (500 MHz, CDCl_3_) *δ* 2.83 (q, *J* = 7.5 Hz, 4H), 2.64–2.58 (m, 4H), 1.76–1.64 (m, 4H), 1.29 (t, *J* = 7.5 Hz, 6H), 0.97 (t, *J* = 7.4 Hz, 6H). ^13^C NMR (125 MHz, CDCl_3_) *δ* 140.4 (2C), 135.7 (2C), 129.5 (2C), 29.7 (2C), 22.3 (2C), 22.2 (2C), 16.5 (2C), 14.1 (2C). ESI-HRMS (*m*/*z*) [M + H]^+^ calcd. for C_16_H_25_S_2_^+^, 281.1392, found: 281.1391.
*2-Ethyl-5-isopropyl-6-methyl-3-propylthieno[3,2-b]thiophene* (**2j**), Brown liquid (92 mg, 69% yield); R*_f_* = 0.7 (Hexane); ^1^H NMR (500 MHz, CDCl_3_) *δ* 3.36–3.22 (m, 1H), 2.83 (q, *J* = 7.5 Hz, 2H), 2.64–2.60 (m, 2H), 2.23 (s, 3H), 1.71 (q, *J* = 7.5 Hz, 2H), 1.33–1.27 (m, 9H), 0.98 (t, *J* = 7.3 Hz, 3H). ^13^C NMR (125 MHz, CDCl_3_) *δ* 146.1, 140.4, 136.7, 135.0, 129.8, 123.4, 29.8, 28.9, 24.5 (2C), 22.3, 22.2, 16.6, 14.1, 12.6. ESI-HRMS (*m*/*z*) [M + H]^+^ calcd. for C_15_H_23_S_2_^+^, 267.1236, found: 267.1233.
*1,2,3,4,6,7,8,9-Octahydrobenzo[b]benzo[4,5]thieno[2,3-d]thiophene* (**2k**), Yellow solid (77 mg, 62% yield); R*_f_* = 0.7 (Hexane); MP: 126–128 °C. ^1^H NMR (400 MHz, CDCl_3_) *δ* 2.88–2.85 (m, 4H), 2.67–2.63 (m, 4H), 1.97–1.82 (m, 8H). ^13^C NMR (100 MHz, CDCl_3_) *δ* 135.3 (2C), 134.9 (2C), 127.8 (2C), 26.0 (2C), 24.6 (2C), 23.5 (2C), 22.5 (2C). ESI-HRMS (*m*/*z*) [M + H]^+^ calcd. for C_14_H_17_S_2_^+^, 249.0766, found: 249.0767.
*2-Propyl-1,2,3,4,6,7,8,9-octahydrobenzo[b]benzo[4,5]thieno[2,3-d]thiophene* (**2l**), Yellow solid (103 mg, 71% yield); R*_f_* = 0.7 (Hexane); MP: 99–101 °C. ^1^H NMR (400 MHz, CDCl_3_) *δ* 2.95–2.90 (m, 1H), 2.86–2.82 (m, 2H), 2.72–2.66 (m, 1H), 2.64–2.28 (m, 3H), 2.52–2.44 (m, 1H), 2.00–1.95 (m, 1H), 1.92–1.83 (m, 5H),1.52–1.37 (m, 5H), 0.94 (t, *J* = 6.4 Hz, 3H). ^13^C NMR (100 MHz, CDCl_3_) *δ* 135.3, 135.2, 127.9, 127.8, 38.1, 34.6, 32.3, 28.9, 26.0, 24.6, 24.3, 23.5, 22.5, 20.1, 14.2. ESI-HRMS (*m*/*z*) [M + H]^+^ calcd. for C_17_H_23_S_2_^+^, 291.1235, found: 291.1233.
*2-(Tert-butyl)-1,2,3,4,6,7,8,9-octahydrobenzo[b]benzo[4,5]thieno[2,3-d]thiophene* (**2m**), Yellow solid (112 mg, 74% yield); R*_f_* = 0.7 (Hexane); MP: 136–138 °C. ^1^H NMR (400 MHz, CDCl_3_) *δ* 2.92–2.84 (m, 3H), 2.78–2.72 (m, 1H), 2.65–2.54 (m, 4H), 2.09–2.05 (m, 1H), 1.92–1.85 (m, 4H), 1.64–1.56 (m, 1H), 1.49–1.38 (m, 1H), 0.98 (s, 9H). ^13^C NMR (100 MHz, CDCl_3_) *δ* 136.2, 135.3, 135.2, 134.5, 127.9, 127.9, 45.5, 32.5, 27.6, 27.3 (3C), 26.0, 25.5, 24.6, 24.1, 23.5, 22.5. ESI-HRMS (*m*/*z*) [M + H]^+^ calcd. for C_18_H_25_S_2_^+^, 305.1392, found: 305.1391.
*3-Methyl-6-(thiophen-2-yl)thieno[3,2-b]thiophene* (**2n**), Black liquid (86 mg, 73% yield); R*_f_* = 0.7 (Hexane); ^1^H NMR (400 MHz, CDCl_3_) *δ* 7.47 (s, 1H), 7.39–7.38 (m, 1H), 7.29–7.28 (m, 1H), 7.13–7.10 (m, 1H), 7.06 (s, 1H), 2.40 (s, 3H). ^13^C NMR (100 MHz, CDCl_3_) *δ* 140.8, 137.5, 136.6, 130.0, 128.7, 127.7, 124.2, 123.8, 122.6, 121.0, 14.6. ESI-HRMS (*m*/*z*) [M + H]^+^ calcd. for C_11_H_9_S_3_^+^, 236.9861, found: 236.9861.
*2-Methyl-5-(6-methylthieno[3,2-b]thiophen-3-yl)furan* (**2o**), Yellow solid (66 mg, 56% yield); R*_f_* = 0.7 (Hexane); MP: 114–116 °C. ^1^H NMR (400 MHz, CDCl_3_) *δ* 7.49 (s, 1H), 7.04 (s, 1H), 6.51 (s, 1H), 6.09 (s, 1H), 2.39 (s, 6H). ^13^C NMR (100 MHz, CDCl_3_) *δ* 151.7, 147.8, 140.6, 129.8, 125.6, 122.5, 118.9, 107.4, 106.8, 14.6, 13.6. ESI-HRMS (*m*/*z*) [M + H]^+^ calcd. for C_12_H_11_OS_2_^+^, 235.0246, found: 235.0247.
*3-Methyl-6-(p-tolyl)thieno[3,2-b]thiophene* (**2p**), Brown solid (73 mg, 68% yield); R*_f_* = 0.5 (Hexane); MP: 135–137 °C. ^1^H NMR (400 MHz, CDCl_3_) *δ* 7.66 (s, 1H), 7.64 (s, 1H), 7.44 (s, 1H), 7.27 (d, *J* = 8.0 Hz, 2H), 7.04 (s, 1H), 2.40 (s, 6H). ^13^C NMR (125 MHz, CDCl_3_) *δ* 140.9, 137.4, 137.1, 135.1, 132.0, 130.0, 129.6 (2C), 126.3 (2C), 122.2, 121.1, 21.2, 14.6. ESI-HRMS (*m*/*z*) [M + H]^+^ calcd. for C_14_H_13_S_2_^+^, 213.0732, found: 213.0733.
*2-Ethyl-6-methyl-3-phenylthieno[3,2-b]thiophene* (**2q**), Yellow liquid (72 mg, 56% yield); R*_f_* = 0.5 (Hexane); ^1^H NMR (400 MHz, CDCl_3_) *δ* 7.56–7.34 (m, 5H), 6.90 (s, 1H), 2.97 (q, *J* = 7.5 Hz, 2H), 2.38 (s, 3H), 1.33 (t, *J* = 7.5 Hz, 3H). ^13^C NMR (100 MHz, CDCl_3_) *δ* 144.1, 139.7, 136.3, 135.3, 130.8, 130.0, 128.7 (2C), 128.6 (2C), 127.4, 120.4, 23.0, 16.7, 14.7. ESI-HRMS (*m*/*z*) [M + H]^+^ calcd. for C_15_H_15_S_2_^+^, 259.0610, found: 259.0609.
*3-(4-Methoxyphenyl)-6-phenylthieno[3,2-b]thiophene* (**2r**), Yellow solid (111 mg, 69% yield); R*_f_* = 0.5 (Hexane); MP: 143–145 °C. ^1^H NMR (400 MHz, CDCl_3_) *δ* 7.89–7.33 (m, 9H), 7.05–6.99 (m, 2H), 3.88 (s, 3H). ^13^C NMR (100 MHz, CDCl_3_) *δ* 159.2, 138.2, 138.1, 134.9, 134.6, 134.5, 128.9 (2C), 127.72 (2C), 127.70, 127.4, 126.5 (2C), 122.2, 120.9, 114.4 (2C), 55.3. ESI-HRMS (*m*/*z*) [M + H]^+^ calcd. for C_19_H_15_OS_2_^+^, 323.0559, found: 323.0556.
*3-Benzyl-6-ethyl-2-phenylthieno[3,2-b]thiophene* (**2s**), Yellow liquid (132 mg, 79% yield); R*_f_* = 0.5 (Hexane); ^1^H NMR (400 MHz, CDCl_3_) *δ* 7.52–7.13 (m, 10H), 6.89 (s, 1H), 4.12 (s, 2H), 2.84–2.77 (m, 2H), 1.28–1.24 (m, 3H). ^13^C NMR (125 MHz, CDCl_3_) *δ* 149.4, 139.1, 139.1, 138.94, 136.1, 134.7, 129.3 (2C), 128.6 (2C), 128.6 (2C), 128.4 (2C), 128.2, 127.6, 126.3, 115.6, 34.2, 24.3, 15.7. ESI-HRMS (*m*/*z*) [M + H]^+^ calcd. for C_21_H_19_S_2_^+^, 335.0923, found: 335.0919.
*3,6-Dibenzyl-2-phenylthieno[3,2-b]thiophene* (**2t**), Yellow liquid (145 mg, 77% yield); R*_f_* = 0.5 (Hexane); ^1^H NMR (500 MHz, CDCl_3_) *δ* 7.51–7.27 (m, 9H), 7.25–7.15 (m, 6H), 6.91 (s, 1H), 4.14 (s, 2H), 4.11 (s, 2H). ^13^C NMR (125 MHz, CDCl_3_) *δ* 145.9, 140.1, 139.7, 139.5, 138.8, 136.1, 133.6, 134.6, 129.3 (2C), 128.6 (2C), 128.6 (2C), 128.5 (2C), 128.4 (2C), 128.2, 127.7 (2C), 126.6, 126.3, 117.5, 37.1, 34.1. ESI-HRMS (*m*/*z*) [M]^+^ calcd. for C_26_H_20_S_2_^+^, 396.1001, found: 396.0998.

### 3.3. General Procedures for the Preparation of Compounds ***3a***–***3b***

A mixture of 2,5-dimethylhex-3-yne-2,5-diol (71 mg, 0.5 mmol), Se powder (78 mg, 1.0 mmol), I_2_ (127 mg, 0.5 mmol), and NMP (2 mL) was added successively into a 20 mL Schlenk tube. The Schlenk tube was inserted in a constant temperature and pressure reactor at 140 °C with stirring for 8 h. After the reaction had completed, this was cooled down to room temperature, and quenched with 10 mL of brine and extracted with ethyl acetate (2 × 5 mL). The combined organic layer was dried over Na_2_SO_4_, filtered, and concentrated under vacuum. The residue was purified by medium pressure preparative chromatography (hexane, R*_f_* = 0.7) on silica gel, which furnished product **3a** (91.1 mg, 69% yield) as a yellow solid.

*3,6-Dimethylselenopheno[3,2-b]selenophene* (**3a**), Yellow solid (87 mg, 69% yield); R*_f_* = 0.7 (Hexane); MP: 96–98 °C. ^1^H NMR (400 MHz, CDCl_3_) *δ* 7.47 (s, 2H), 2.37 (s, 6H). ^13^C NMR (100 MHz, CDCl_3_) *δ* 141.6 (2C), 135.2 (2C), 123.6 (2C), 17.2 (2C). ESI-HRMS (*m*/*z*) [M + H]^+^ calcd. for C_8_H_9_Se_2_^+^, 252.9148, found: 252.9158.
*2,3,5,6-Tetramethylselenopheno[3,2-b]selenophene* (**3b**), Brown solid (85 mg, 64% yield); R*_f_* = 0.7 (Hexane); MP: 101–103 °C. ^1^H NMR (400 MHz, CDCl_3_) *δ* 2.51 (s, 6H), 2.16 (s, 6H). ^13^C NMR (100 MHz, CDCl_3_) *δ* 137.6 (2C), 135.8 (2C), 130.2 (2C), 16.2 (2C), 14.5 (2C). ESI-HRMS (*m*/*z*) [M + H]^+^ calcd. for C_10_H_13_Se_2_^+^, 280.9461, found: 280.9459.

### 3.4. Synthesis of 2,5-Diiodo-3,6-dimethylthieno[3,2-b]thiophene ***4a***

A mixture of 3,6-dimethylthieno[3,2-*b*]thiophene **2a** (336 mg, 2 mmol), chloroform–acetic acid (1:1 *v*/*v*, 10 mL) was added to N-iodosuccinimide (NIS) (563 mg, 2.5 mmol) in a 50 mL round bottom flask. The round bottom flask was kept at 40 °C with stirring for 3 h. After the reaction had completed, the reaction quenched with 10 mL of brine and extracted with ethyl acetate (3 × 15 mL). The combined organic layer was dried over Na_2_SO_4_, filtered, and concentrated under vacuum. The residue was purified by medium pressure preparative chromatography (Hexane, R*_f_* = 0.7) on silica gel, which furnished product **4a** (512 mg, 61% yield) as a pale yellow solid.

*2,5-Diiodo-3,6-dimethylthieno[3,2-b]thiophene* (**4a**), White solid (512 mg, 61% yield); R*_f_* = 0.7 (Hexane); ^1^H NMR (400 MHz, CDCl_3_) *δ* 2.26 (s, 1H). ^13^C NMR (100 MHz, CDCl_3_) *δ* 140.9 (2C), 134.2 (2C), 76.0 (2C), 16.9 (2C).

### 3.5. Synthesis of ((3,6-Dimethylthieno[3,2-b]thiophene-2,5-diyl)bis(ethyne-2,1-diyl))bis(trimethylsilane) ***4b***

Under the N_2_ condition, a mixture of 2,5-diiodo-3,6-dimethylthieno[3,2-*b*]thiophene **4a** (100 mg, 0.24 mmol), ethynyltrimetilsilane (71 mg, 0.72 mmol), PdCl_2_ (PPh_3_)_2_ (17 mg, 10 mol%), CuI (5 mg, 10 mol%), THF (2 mL), and Et_3_N (1 mL) was added successively into a 20 mL Schlenk tube. The Schlenk tube was then immersed in an oil bath at 60 °C with stirring for 12 h. After cooling down to room temperature, the solution was filtered through a small amount of silica gel. The residue was then concentrated in vacuo and the crude was purified by medium pressure preparative chromatography with n-hexane to afford the 3,6-dimethyl-2,5-bis(phenylethynyl)thieno[3,2-*b*]thiophene **4b** (67 mg, 78% yield) as a yellow solid.

*((3,6-Dimethylthieno[3,2-b]thiophene-2,5-diyl)bis(ethyne-2,1-diyl))bis(trimethylsilane)* (**4b**), Yellow solid (67 mg, 78% yield); MP:157–158 °C. R*_f_* = 0.7 (Hexane); ^1^H NMR (400 MHz, CDCl_3_) *δ* 2.35 (s, 6H), 0.27 (s, 18H). ^13^C NMR (100 MHz, CDCl_3_) *δ* 138.0 (2C), 135.7 (2C), 121.2 (2C), 103.5 (2C), 97.6 (2C), 14.0 (2C), 0.0 (6C). ESI-HRMS (*m*/*z*) [M + Na]^+^ calcd. for C_18_H_24_NaS_2_Si_2_^+^, 383.0750, found: 383.0752.

### 3.6. Synthesis of 2,5-Diethynyl-3,6-dimethylthieno[3,2-b]thiophene ***4c***

Under the N_2_ condition, a mixture of (3,6-dimethylthieno[3,2-*b*]thiophene-2,5-diyl)bis(ethyne-2,1-diyl))bis(trimethylsilane) **4b** (180 mg, 0.5 mmol), CH_3_OH (2 mL), and K_2_CO_3_ (1 mmol) was added successively into a 20 mL Schlenk tube. The Schlenk tube was then at room temperature stirring for 3 h. The solution was filtered through a small amount of silica gel. Then the residue was concentrated in vacuo and the crude was purified by medium pressure preparative chromatography with n-hexane to afford the 2,5-diethynyl-3,6-dimethylthieno[3,2-*b*]thiophene **4c** in 92% yield.

*2,5-Diethynyl-3,6-dimethylthieno[3,2-b]thiophene* (**4c**), White solid (46 mg, 92% yield); R*_f_* = 0.5 (Hexane); MP:123–124 °C. ^1^H NMR (400 MHz, CDCl_3_) *δ* 3.62 (s, 2H), 2.39 (s, 6H). ^13^C NMR (100 MHz, CDCl_3_) *δ* 138.1 (2C), 136.2 (2C), 120.2 (2C), 85.5 (4C), 13.9 (2C). ESI-HRMS (*m*/*z*) [M + Na]^+^ calcd. for C_24_H_16_NaS_2_^+^, 238.9960, found: 238.9951.

### 3.7. Synthesis of 3,6-Dimethyl-2,5-bis(phenylethynyl)thieno[3,2-b]thiophene ***4d***

Under the N_2_ condition, a mixture of 2,5-diiodo-3,6-dimethylthieno[3,2-*b*]thiophene **4a** (100 mg, 0.24 mmol), ethynylbenzene (97 mg, 0.96 mmol), PdCl_2_ (PPh_3_)_2_ (17 mg, 10 mol%), CuI (5 mg, 10 mol%), THF (2 mL), and Et_3_N (1 mL) was added successively into a 20 mL Schlenk tube. The Schlenk tube was then immersed in an oil bath at 60 °C with stirring for 12 h. After cooling down to room temperature, the solution was filtered through a small amount of silica gel. The residue was then concentrated in vacuo and the crude was purified by medium pressure preparative chromatography with n-hexane to afford the 3,6-dimethyl-2,5-bis(phenylethynyl)thieno[3,2-*b*]thiophene **4d** in 56% yield as a yellow solid.

*3,6-Dimethyl-2,5-bis(phenylethynyl)thieno[3,2-b]thiophene* (**4d**), Yellow solid (49 mg, 56% yield); R*_f_* = 0.7 (Hexane); MP:137–139 °C. ^1^H NMR (400 MHz, CDCl_3_) *δ* 7.57–7.52 (m, 4H), 7.40–7.33 (m, 6H), 2.46 (s, 6H). ^13^C NMR (100 MHz, CDCl_3_) *δ* 138.5 (2C), 134.9 (2C), 131.3 (4C), 128.4 (2C), 128.4 (4C), 122.9 (2C), 121.0 (2C), 97.5 (2C), 82.9 (2C), 14.1 (2C). ESI-HRMS (*m*/*z*) [M + Na]^+^ calcd. for C_24_H_16_NaS_2_^+^, 391.0586, found: 391.0580.

### 3.8. Synthesis of 3,6-Dimethyl-2,5-diphenylthieno[3,2-b]thiophene ***4e***

Under the N_2_ condition, a mixture of 2,5-diiodo-3,6-dimethylthieno[3,2-*b*]thiophene **4a** (100 mg, 0.24 mmol), phenylboric acid (88 mg, 0.72 mmol), Pd (PPh_3_)_4_ (55 mg, 20 mol%), Cs_2_CO_3_ (157 mg, 2 equiv.), THF (2 mL), and H_2_O (0.2 mL) was added successively into a 20 mL Schlenk tube. The Schlenk tube was then immersed in an oil bath at 60 °C with stirring for 12 h. After the reaction had completed, the reaction was quenched with 10 mL of brine and extracted with ethyl acetate (3 × 15 mL). The combined organic layer was dried over Na_2_SO_4_, filtered, and concentrated under vacuum, and the crude was purified by medium pressure preparative chromatography with n-hexane to afford the 3,6-dimethyl-2,5-diphenylthieno[3,2-*b*]thiophene **4e** in 68% yield as a yellow solid.

*3,6-Dimethyl-2,5-diphenylthieno[3,2-b]thiophene* (**4e**), Yellow solid (67 mg, 68% yield); R*_f_* = 0.7 (Hexane); MP:168–170 °C. ^1^H NMR (400 MHz, CDCl_3_) *δ* 7.53 (d, *J* = 7.3 Hz, 4H), 7.45 (t, *J* = 7.6 Hz, 4H), 7.36 (d, *J* = 7.9 Hz, 2H), 2.44 (s, 6H). ^13^C NMR (100 MHz, CDCl_3_) *δ* 139.1 (2C), 138.1 (2C), 135.1 (2C), 129.1 (4C), 128.6 (4C), 127.4 (2C), 125.8 (2C), 14.0 (2C). ESI-HRMS (*m*/*z*) [M + H]^+^ calcd. for C_20_H_17_S_2_^+^, 321.0766, found: 321.0761.

### 3.9. Synthesis of 2,5-Diiodo-2,5-dimethylhex-3-yne ***5b***

A mixture of 2,5-dimethylhex-3-yne-2,5-diol (71 mg, 0.5 mmol), I_2_ (254 mg, 1.0 mmol), and NMP (2 mL) was added successively into a 20 mL Schlenk tube. The Schlenk tube was then immersed in an oil bath at 140 °C with stirring for 8 h. After cooling down to room temperature, the reaction was quenched with 10 mL of brine and extracted with ethyl acetate (3 × 5 mL). The combined organic layer was dried over Na_2_SO_4_, filtered, and concentrated under vacuum. The residue was purified by medium pressure preparative chromatography (hexane/EtOAc = 30:1, R*_f_* = 0.6) on silica gel, which furnished product **5b** (141 mg, 78% yield) as a yellow liquid.

*2,5-Diiodo-2,5-dimethylhex-3-yne* (**5b**), Yellow liquid (141 mg, 78% yield); R*_f_* = 0.6 (Hexane/EtOAc = 30:1); ^1^H NMR (400 MHz, CDCl_3_) *δ* 1.36 (s, 12H). ^13^C NMR (100 MHz, CDCl_3_) *δ* 113.0 (2C), 91.2 (2C), 28.9 (42C). ESI-HRMS (*m*/*z*) [M + Na]^+^ calcd. for C_8_H_12_NaI_2_^+^, 384.8921, found: 384.8926.

### 3.10. Synthesis of 2,4-Diphenylthiophene ***5d***

A mixture of ethynylbenzene (51 mg, 0.5 mmol), Na_2_S_4_O_6_·2H_2_O (460 mg, 1.5 mmol), and NMP (2 mL) was added successively into a 20 mL Schlenk tube. The Schlenk tube was then immersed in an oil bath at 140 °C with stirring for 8 h. After cooling down to room temperature, the reaction was quenched with 10 mL of brine and extracted with ethyl acetate (2× 5 mL). The combined organic layer was dried over Na_2_SO_4_, filtered, and concentrated under vacuum. The residue was purified by medium pressure preparative chromatography (hexane, R*_f_* = 0.7) on silica gel, which furnished 2,4-diphenylthiophene product **5d** (20.6 mg, 35% yield) as a pale yellow solid.

*2,4-Diphenylthiophene* (**5d**), Yellow solid (20.6 mg, 35% yield); R*_f_* = 0.5 (Hexane); ^1^H NMR (400 MHz, CDCl_3_) *δ* 7.67–7.61 (m, 4H), 7.60 (s, 1H), 7.44–7.39 (m, 5H), 7.33–7.31 (m, 2H); ^13^C NMR (100 MHz, CDCl_3_) *δ* 145.0, 143.1, 135.9, 134.3, 128.9 (2C), 128.8 (2C), 127.7, 127.3, 126.3 (2C), 125.8 (2C), 122.3, 119.7. ESI-HRMS (*m*/*z*) [M + H]^+^ calcd. for C_16_H_13_S^+^, 237.0732, found: 237.0734.

### 3.11. Synthesis of 3,6-Dimethylthieno[3,2-b]thiophene-2,5-d2 ***2a-D***

A mixture of 2,5-dimethylhex-3-yne-2,5-diol **1a** (71 mg, 0.5 mmol), Na_2_S_2_O_3_ (158 mg, 1.0 mmol), I_2_ (127 mg, 0.5 mmol), D_2_O (0.5 mL), and NMP (2 mL) was added successively into a 20 mL Schlenk tube. The Schlenk tube was then immersed in an oil bath at 140 °C with stirring for 8 h. After cooling down to room temperature, the reaction was quenched with 10 mL of brine and extracted with ethyl acetate (2 × 5 mL). The combined organic layer was dried over Na_2_SO_4_, filtered, and concentrated under vacuum. The residue was purified by medium pressure preparative chromatography (hexane, R*_f_* = 0.7) on silica gel, which furnished product **2a-D** (47.6 mg, 56% yield) with 27% deuteration as a yellow solid.

*3,6-Dimethylthieno[3,2-b]thiophene-2,5-d2* (**2a-D**), Yellow solid (68 mg, 81% yield); R*_f_* = 0.7 (Hexane); ^1^H NMR (400 MHz, CDCl_3_) δ 6.96 (s, 1.68H), 2.36 (s, 6H). ^13^C NMR (100 MHz, CDCl_3_) δ 140.0 (2C), 130.3 (2C), 121.8 (2C), 14.6 (2C). ^2^H NMR (77 MHz, CH_2_Cl_2_) δ 6.96 (s, 2D).

## 4. Conclusions

In conclusion, a method for synthesizing multisubstituted thieno[3,2-*b*]thiophene and selenopheno[3,2-*b*]selenophene derivatives using alkynyl diols and Na_2_S_2_O_3_ and selenium under transition metal-free conditions was proposed herein. The proposed method used iodine-mediated dehydration and sulfur cyclization to afford various functionalized thieno[3,2-*b*]thiophenes and selenopheno[3,2-*b*]selenophenes in moderate to good yields. The existing synthesis methods use 3-halothiophenes as starting materials and require multistep synthesis as well as several additional chemical reagents. In contrast, the proposed method is economical and easily yields alkynyl diols for thieno[3,2-*b*]thiophene synthesis with step efficiency. In this cyclization process, the role of I_2_ may be to react with Na_2_S_2_O_3_ to generate sulfur radicals, while Na_2_S_4_O_6_ alone can decompose to give sulfur radicals under reaction conditions. Therefore, this sulfur cyclization reaction can be effectively achieved using either the I_2_/Na_2_S_2_O_3_ or Na_2_S_4_O_6_ system. We aim to apply the proposed method to synthesize thieno[3,2-*b*]thiophene core molecules and expand their applicability in optoelectronic materials.

## Figures and Tables

**Table 1:** Optimization reaction conditions *^(^^a)^*.

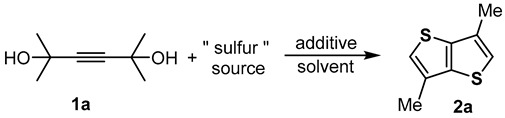				
Entry	S Sources	Solvents	Additives (mmol)	Yields (%) *^(b)^*
1	S_8_	NMP		21
2	EtOCS_2_K	NMP		trace
3	Thiourea	NMP		16
4	K_2_S	NMP		trace
5	Na_2_S·9H_2_O	NMP		trace
6	Na_2_S_2_O_3_	NMP		31
7 *^(c)^*	Na_2_S_2_O_3_	NMP		33
8	S_8_	NMP	I_2_ (0.5)	63
9	Na_2_S_2_O_3_	NMP	I_2_ (0.5)	79
10	Na_2_S_2_O_3_	NMP	NH_4_I (0.5)	13
11	Na_2_S_2_O_3_	NMP	HCl (1 M, 0.5 mL)	57
12	Na_2_S_2_O_3_	NMP	I_2_ (0.25)	61
13	Na_2_S_2_O_3_	NMP	I_2_ (0.75)	77
14	Na_2_S_2_O_3_	DMSO	I_2_ (0.5)	12
15	Na_2_S_2_O_3_	DMF	I_2_ (0.5)	45
16	Na_2_S_2_O_3_	DMAc	I_2_ (0.5)	61
17	Na_2_S_2_O_3_	AcOH	I_2_ (0.5)	trace
18* ^(d)^*	Na_2_S_2_O_3_	NMP	I_2_ (0.5)	82
19* ^(e)^*	Na_2_S_2_O_3_	NMP	I_2_ (0.5)	55
20* ^(f)^*	Na_2_S_2_O_3_	NMP	I_2_ (0.5)	81
21* ^(g)^*	Na_2_S_2_O_3_	NMP	I_2_ (0.5)	80
22 *^(h)^*	Na_2_S_2_O_3_	NMP	I_2_ (0.5)	78

*^(a)^* Reaction conditions: 2,5-dimethylhex-3-yne-2,5-diol **1a** (0.5 mmol), sulfur source (1.0 mmol), solvent (2 mL), and an additive at 150 °C for 10 h in a sealed tube under an air atmosphere. *^(b)^* Isolated yields. *^(c)^* sulfur source (1.50 mmol); *^(d)^* 140 °C, *^(e)^* 130 °C, *^(f)^* 140 °C, 8 h, *^(g)^* 140 °C, 12 h; *^(h)^* DDQ (2,3-dichloro-5,6-dicyano-1,4-benzoquinone) (0.5 mmol) was added.

## Data Availability

All data are available from the authors.

## Supplementary Materials

The following supporting information can be downloaded at: https://www.mdpi.com/article/10.3390/molecules29235507/s1, NMR spectra of compounds **2a**–**2t**, **3a**–**3b** and **4a**–**4e**, **5b**, **5d**. HRMS for compounds **2a**–**2t**, **3a**–**3b**, and **4a**–**4e**, **5b**, **5d**. References [42,61,62,63] are cited in the Supplementary Materials.
