# Supplementary material for: Expedient Synthesis of Substituted Thieno[3,2-b]thiophenes and Selenopheno[3,2-b]selenophenes Through Cascade Cyclization of Alkynyl Diol Derivatives
Source: Molecules. 2024 Nov 21;29(23):5507. doi: 10.3390/molecules29235507 (PMC11643606; doi:10.3390/molecules29235507)

Supporting Information

Expedient Synthesis of Substituted Thieno[3,2-*b*]thiophenes and selenopheno[3,2-*b*]selenophenes through Cascade Cyclization of Alkynyl Diol Derivatives

Yingqi Feng [1], Xuelin Zhang [1], Ziqing He [1], Miaoshan Zhao [1], Lu Chen [1], Yibiao Li [1,*] and Xiai Luo [2,*]

[1] Jiangmen Key Laboratory of Synthetic Chemistry and Cleaner Production, School of Environmental & Chemical Engineering, Wuyi University, Jiangmen 529020, China

[2] Hunan Province Key Laboratory for Synthetic Biology of Traditional Chinese Medicine, School of Pharmaceutical Sciences, Hunan University of Medicine, Huaihua, 418000, China;

***** Correspondence:luoxiai83@163.com (X. Luo); leeyib268@126.com (Y. Li)

Table of Contents

A. General Information 2

B. General reaction procedures 2

C. Crystal structure 4

D. Characterization data for all prepared compounds 16

E. ^{1}H NMR and ^{13}C NMR spectra for products 25

A. General Information

Chemicals and solvents were purchased from commercial suppliers, such as Bidepharm and Energy Chemical, and used as received unless noted. All products were purified by flash chromatography on silica gel. The chemical yields referred are isolated products. ^{1}H NMR and ^{13}C NMR spectra were recorded on 400 MHz, 500MHz and 600 MHz Bruker spectrometers. Chemical shifts of ^{1}H were reported in part per million relative to the $CDCl_3$ residual peak (δ 7.260). Chemical shifts of ^{13}C NMR were reported relative to $CDCl_3$ (δ 77.0). The used abbreviations are as follows: s (singlet), d (doublet), t (triplet), quart. (quartet), quint (quintet), m (multiplet), br (broad). Multiplets which arise from accidental equality of coupling constants of magnetically non-equivalent protons are marked as virtual (*virt.*). High resolution mass spectra (HRMS) data were measured on a ESI-microTOF II. The crystal data were collected by a diffractometer Rigaku Oxford Diffraction Supernova Dual Source, Cu at Zero equipped with an AtlasS2 CCD using Cu Kα radiation (1.54178 Å) by using a w scan mode. Major elements, such as Zn, Cd, Cu, Ni, Cr et al. in reaction system were determined by using Microwave plasma-atomic emission spectrometry (Agilent/4210 MP-AES). Melting points were measured on a SGW® X-4B and are not corrected. Reactions were monitored by TLC analysis using silica gel 60 Å F-254 thin layer plates and compounds were visualized with a UV light at 254 nm or 365 nm.

B. General reaction procedures

General methods for the synthesis of alkynyl diols

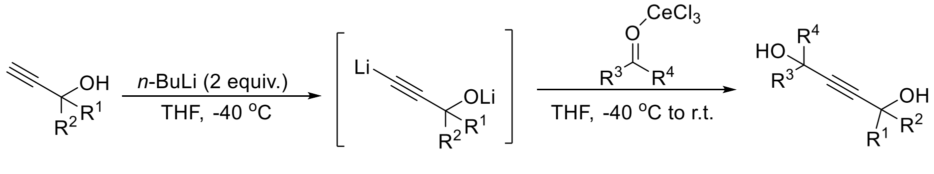

1b-1c, 1l-1n, and **1r** were synthesized according to previously reported procedures:[1] To a round bottom flask containing the propargylic alcohol (3 mmol) in THF (5 mL) at -40 °C under a dry N_2 atmosphere with stirring, n-butyl lithium (6.6 mmol, 7.2 mL, 0.92 mol/L solution in hexane) was added dropwise. After stirring the solution at the same temperature for 20 min, it was transferred to another round bottomed flask

containing a previously prepared mixture of the carbonyl compound (3 mmol) and anhydrous $CeCl_3$ (1.5 mmol) in THF (10 mL). The reaction was monitored by TLC until consumption of the carbonyl compound, quenched with a saturated solution of NH_4Cl (2 mL) and the phases separated. The aqueous phase was extracted with ethyl acetate (2*5 mL) washed with brine, dried with magnesium sulphate, filtered, and the solvents removed using a rotary evaporator under vacuum. The crude was purified by silica gel chromatography using n-hexane/ethyl acetate (1:1) as eluent.

Scheme S1. Synthesis of alkynyl diols from alkylols

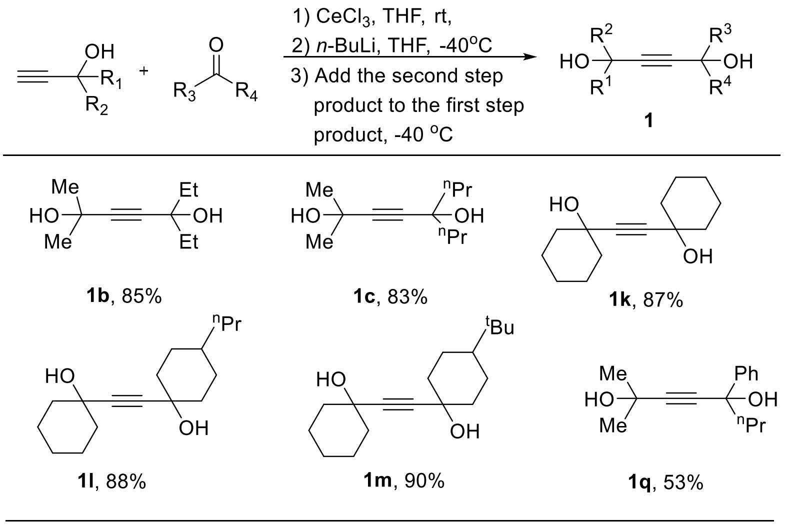

Scheme S2. Alkynyl diols for sulfur cyclization reaction

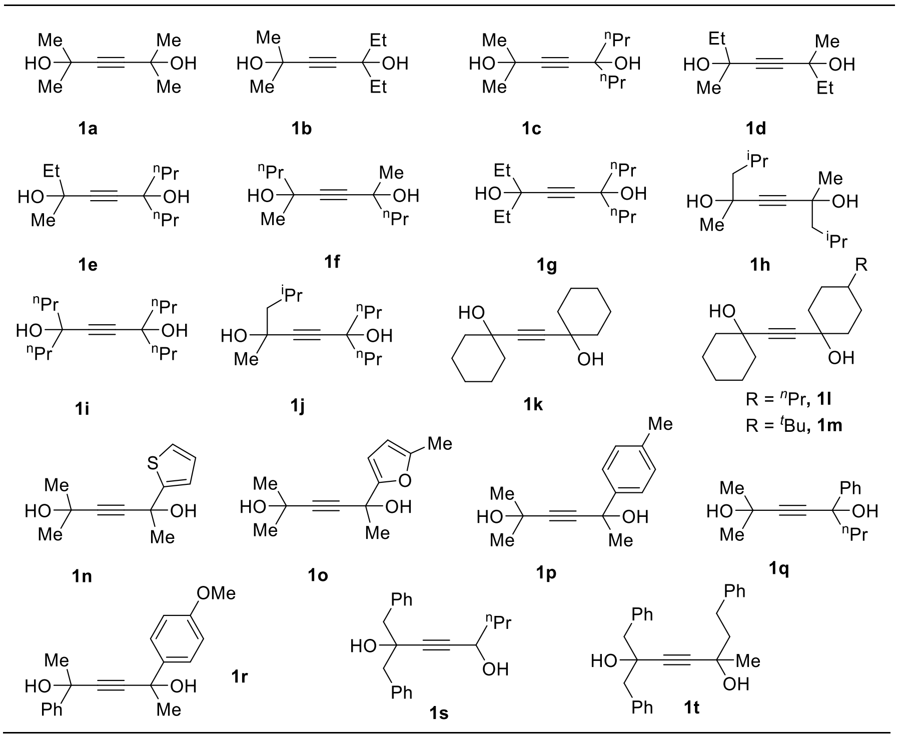

General methods for the synthesis of thieno[3,2-*b*]thiophenes

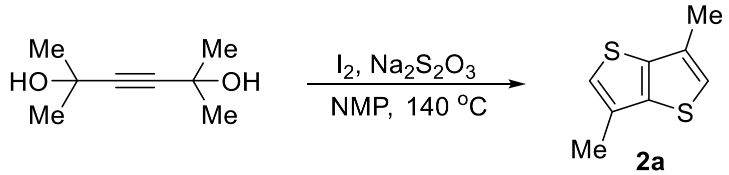

2a

A mixture of 2,5-dimethylhex-3-yne-2,5-diol (71 mg, 0.5 mmol), $Na_2S_2O_3$ (158 mg, 1.0 mmol), I_2 (127 mg, 0.5 mmol), and NMP (2 mL) was added successively in a 20 mL Schlenk tube. The Schlenk tube was then immersed in an oil bath at 140 °C stirring for 8 h. After cooling down to room temperature, the reaction quenched with 10 mL of brine and extracted with ethyl acetate (2*5 mL). The combined organic layer was dried over Na_2SO_4, filtered and concentrated under vacuum. The residue was purified by flash chromatography (Hexane, R_f = 0.7) on silica gel, which furnished product **2a** (68 mg, 81% yield) as a pale yellow solid. The following compounds **2b-2v** were prepared by a similar method, unless otherwise note.

General methods for the synthesis of 3,6-dimethylthieno[3,2-*b*]thiophene 2a in 1 mmol-scale

A mixture of 2,5-dimethylhex-3-yne-2,5-diol (142 mg, 1 mmol), $Na_2S_2O_3$ (316 mg, 2 mmol), I_2 (253 mg, 1 mmol), and NMP (2.5 mL) was added successively in a 20 mL Schlenk tube. The Schlenk tube was then immersed in an oil bath at 140 °C stirring for 8 h. After cooling down to room temperature, the reaction quenched with 10 mL of brine and extracted with ethyl acetate (2*5 mL). The combined organic layer was dried over Na_2SO_4, filtered and concentrated under vacuum. The residue was purified by flash chromatography (Hexane, R_f = 0.7) on silica gel, which furnished product **2a** (131 mg, 78% yield) as a pale yellow solid.

General methods for the synthesis of selenopheno[3,2-*b*]selenophenes

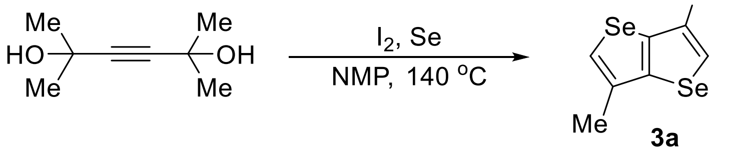

3a

A mixture of 2,5-dimethylhex-3-yne-2,5-diol (71 mg, 0.5 mmol), Se powder (78 mg, 1.0 mmol), I_2 (127 mg, 0.5 mmol), and NMP (2 mL) was added successively in a 20

mL Schlenk tube. The Schlenk tube was insert in constant temperature and pressure reactor at 140 ^{o}C stirring for 8 h. After the reaction is complete, then cooling down to room temperature, and quenched with 10 mL of brine and extracted with ethyl acetate (2*5 mL). The combined organic layer was dried over Na_2SO_4, filtered and concentrated under vacuum. The residue was purified by Medium Pressure Preparative Chromatography (hexane, R_f = 0.7) on silica gel, which furnished product **3a** (91.1 mg, 69% yield) as a yellow solid.

General methods for the synthesis of 2,5-diiodo-3,6-dimethylthieno[3,2-*b*]thiophene

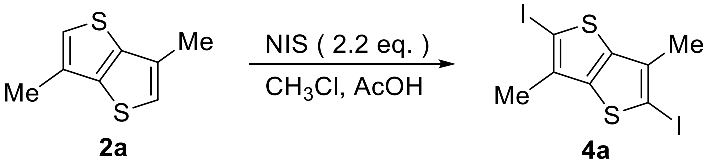

A mixture of 3,6-dimethylthieno[3,2-*b*]thiophene **2a** (336 mg, 2 mmol), chloroform-acetic acid (1:1 v/v, 10 mL), the added N-iodosuccinimide (NIS) (563 mg, 2.5 mmol) in a 50 mL round bottom flask. The round bottom flask was then at 40 °C stirring for 3 h. After the reaction is complete, the reaction quenched with 10 mL of brine and extracted with ethyl acetate (3*15 mL). The combined organic layer was dried over Na_2SO_4, filtered and concentrated under vacuum. The residue was purified by medium pressure preparative chromatography (Hexane, R_f = 0.7) on silica gel, which furnished product **4a** (512 mg, 61% yield) as a pale yellow solid.

General methods for the synthesis of ((3,6-dimethylthieno[3,2-*b*]thiophene-2,5-diyl)bis(ethyne-2,1-diyl))bis(trimethylsilane)

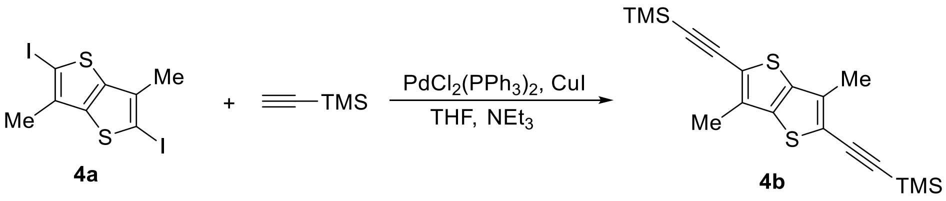

Under N_2 condition, a mixture of 2,5-diiodo-3,6-dimethylthieno[3,2-*b*]thiophene **4a** (100 mg, 0.24 mmol), ethynyltrimetilsilane (71 mg, 0.72 mmol), $PdCl_2(PPh_3)_2$ (17 mg, 10 mol%), CuI (5 mg, 10 mol%), THF (2 mL), Et_3N (1 mL) and was added

successively in a 20 mL Schlenk tube. The Schlenk tube was then immersed in an oil bath at 60 °C stirring for 12 h. After cooling down to room temperature, the solution was filtered through a small amount of silica gel. Then the residue was concentrated in vacuo and the crude was purified by medium pressure preparative chromatography with n-hexane to afford the 3,6-dimethyl-2,5-bis(phenylethynyl)thieno[3,2-*b*]thiophene **4b** (67 mg, 78% yield) as a yellow solid.

General methods for the synthesis of 2,5-diethynyl-3,6-dimethylthieno[3,2-*b*]thiophene

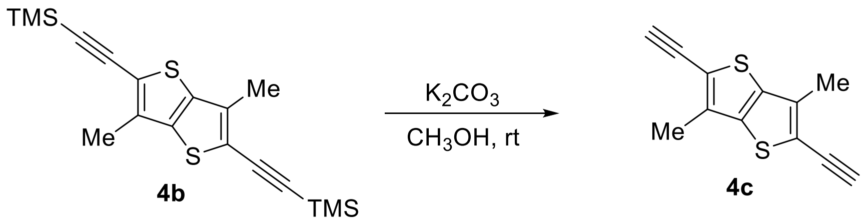

Under N_2 condition, a mixture of (3,6-dimethylthieno[3,2-*b*]thiophene-2,5-diyl)bis(ethyne-2,1-diyl))bis(trimethylsilane) **4b** (180 mg, 0.5 mmol), CH_3OH (2 mL), K_2CO_3 (1 mmol) was added successively in a 20 mL Schlenk tube. The Schlenk tube was then at room temperature stirring for 3 h. The solution was filtered through a small amount of silica gel. Then the residue was concentrated in vacuo and the crude was purified by medium pressure preparative chromatography with n-hexane to afford the 2,5-diethynyl-3,6-dimethylthieno[3,2-*b*]thiophene **4c** in 92% yield.

General methods for the synthesis of 2,5-diiodo-3,6-dimethylthieno[3,2-*b*]thiophene

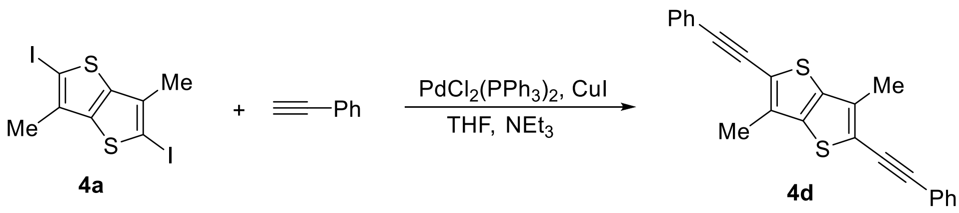

Under N_2 condition, a mixture of 2,5-diiodo-3,6-dimethylthieno[3,2-*b*]thiophene **4a** (100 mg, 0.24 mmol), ethynylbenzene (97 mg, 0.96 mmol), $PdCl_2(PPh_3)_2$ (17 mg, 10 mol%), CuI (5 mg, 10 mol%), THF (2 mL), Et_3N (1 mL) and was added successively

in a 20 mL Schlenk tube. The Schlenk tube was then immersed in an oil bath at 60 °C stirring for 12 h. After cooling down to room temperature, the solution was filtered through a small amount of silica gel. Then the residue was concentrated in vacuo and the crude was purified by medium pressure preparative chromatography with n-hexane to afford the 3,6-dimethyl-2,5-bis(phenylethynyl)thieno[3,2-*b*]thiophene **4d** in 56% yield as a yellow solid.

General methods for the synthesis of 3,6-dimethyl-2,5-diphenylthieno[3,2-*b*]thiophene

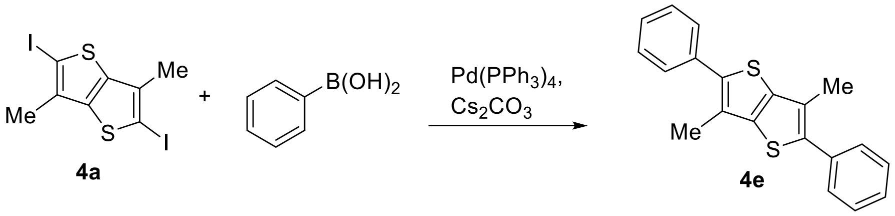

Under N_2 condition, a mixture of 2,5-diiodo-3,6-dimethylthieno[3,2-*b*]thiophene **4a** (100 mg, 0.24 mmol), phenylboric acid (88 mg, 0.72 mmol), $Pd(PPh_3)_4$ (55 mg, 20 mol%), Cs_2CO_3 (157 mg, 2 equiv.), THF (2 mL), H_2O (0.2 mL), was added successively in a 20 mL Schlenk tube. The Schlenk tube was then immersed in an oil bath at 60 °C stirring for 12 h. After the reaction is complete, the reaction quenched with 10 mL of brine and extracted with ethyl acetate (3*15 mL). The combined organic layer was dried over Na_2SO_4, filtered and concentrated under vacuum and the crude was purified by medium pressure preparative chromatography with n-hexane to afford the 3,6-dimethyl-2,5-diphenylthieno[3,2-*b*]thiophene **4e** in 68% yield as a yellow solid.

General methods for Control experiments

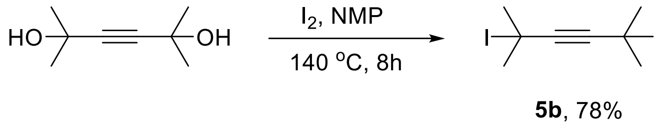

A mixture of 2,5-dimethylhex-3-yne-2,5-diol (71 mg, 0.5 mmol), I_2 (254 mg, 1.0 mmol), and NMP (2 mL) was added successively in a 20 mL Schlenk tube. The Schlenk tube was then immersed in an oil bath at 140 °C stirring for 8 h. After cooling down to room temperature, the reaction quenched with 10 mL of brine and extracted

with ethyl acetate (3*5 mL). The combined organic layer was dried over Na_2SO_4, filtered and concentrated under vacuum. The residue was purified by medium pressure preparative chromatography (hexane/EtOAc =30:1, R_f = 0.6) on silica gel, which furnished product **5ba** (141 mg, 78% yield) as a yellow liquid.

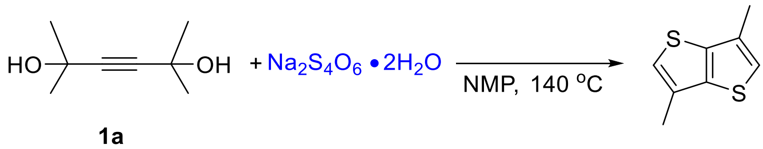

1a

2a,73%

A mixture of 2,5-dimethylhex-3-yne-2,5-diol (71 mg, 0.5 mmol), $Na_2S_4O_6$•$2H_2O$ (306 mg, 1.0 mmol), and NMP (2 mL) was added successively in a 20 mL Schlenk tube. The Schlenk tube was then immersed in an oil bath at 140 °C stirring for 8 h. After cooling down to room temperature, the reaction quenched with 10 mL of brine and extracted with ethyl acetate (2*5 mL). The combined organic layer was dried over Na_2SO_4, filtered and concentrated under vacuum. The residue was purified by medium pressure preparative chromatography (hexane, R_f = 0.7) on silica gel, which furnished product **2a** (61.3 mg, 73% yield) as a pale yellow solid.

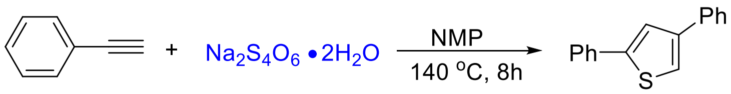

5d, 35%

A mixture of ethynylbenzene (51 mg, 0.5 mmol), $Na_2S_4O_6$•$2H_2O$ (460 mg, 1.5 mmol), and NMP (2 mL) was added successively in a 20 mL Schlenk tube. The Schlenk tube was then immersed in an oil bath at 140 °C stirring for 8 h. After cooling down to room temperature, the reaction quenched with 10 mL of brine and extracted with ethyl acetate (2*5 mL). The combined organic layer was dried over Na_2SO_4, filtered and concentrated under vacuum. The residue was purified by medium pressure preparative chromatography (hexane, R_f = 0.7) on silica gel, which furnished 2,4-diphenylthiophene product **5d** (20.6 mg, 35% yield) as a pale yellow solid.

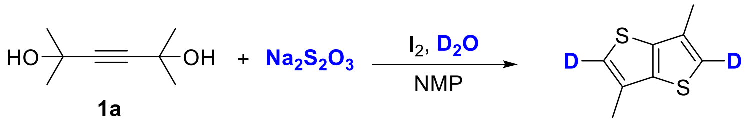

1a

2a-D, 56%

A mixture of 2,5-dimethylhex-3-yne-2,5-diol **1a** (71 mg, 0.5 mmol), $Na_2S_2O_3$ (158 mg, 1.0 mmol), I_2 (127 mg, 0.5 mmol), D_2O (0.5 mL), and NMP (2 mL) was added successively in a 20 mL Schlenk tube. The Schlenk tube was then immersed in an oil bath at 140 ℃ stirring for 8 h. After cooling down to room temperature, the reaction quenched with 10 mL of brine and extracted with ethyl acetate (2*5 mL). The combined organic layer was dried over Na_2SO_4, filtered and concentrated under vacuum. The residue was purified by medium pressure preparative chromatography (hexane, R_f = 0.7) on silica gel, which furnished product **2a-D** (47.6 mg, 56% yield) with 27% deuteration as a yellow solid.

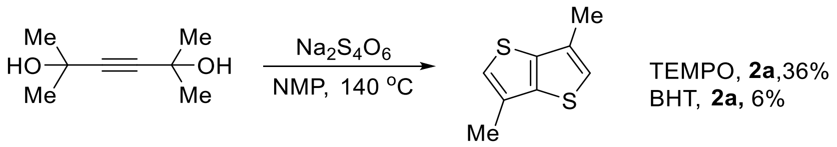

A mixture of 2,5-Dimethylhex-3-yne-2,5-diol (58 mg, 0.4 mmol), $Na_2S_2O_3$ (128 mg, 0.8 mmol), I_2 (102 mg, 0.4 mmol), TEMPO (94 mg, 1.5 eq) or BHT (132 mg, 1.5 eq) and NMP (2 mL) was added successively in reaction tube. The reaction tube was then insert in constant temperature and pressure reactor at 140 ^{o}C stirring for 8 h, then quenched with 10 mL of brine and extracted with ethyl acetate. The combined organic layer was dried over Na_2SO_4, filtered and concentrated under vacuum. The residue was purified by medium pressure preparative chromatography (hexane, R_f = 0.7) on silica gel, which furnished product **2a** as a pale yellow liquid.

C. Crystal structure

Crystal structure of 2d

Experimental: Single crystals of $C_{10}H_{12}S_2$ **(2d)** were generation in the mixed solution of dichloromethane and n-hexane. A suitable crystal was selected and analyzed it on a SuperNova, Dual, Cu at zero, AtlasS2 diffractometer. The crystal was kept at 149.99(10) K during data collection. The X-ray crystallographic structures for **2d**. ORTEP representation with 50% probability thermal ellipsoids. Crystal data have been deposited to CCDC, number 2358238.

Crystal structure determination of 2d

Crystal Data for $C_{10}H_{12}S_2$ (M=98.16 g/mol): monoclinic, space group C2/m (no. 12), a = 9.9600(7) Å, b = 7.0968(5) Å, c = 7.0727(5) Å, β = 102.179(7) , V = 488.68(6) Å3, Z = 4, T = 100.15 K, μ(Cu Kα) = 4.439 mm-1, Dcalc = 1.334 g/cm3, 1684 reflections measured (12.804 ≤ 2Θ ≤ 146.01), 517 unique (Rint = 0.0208, Rsigma = 0.0151) which were used in all calculations. The final R1 was 0.0413 (I > 2σ(I)) and R2 as 0.1164 (all data).

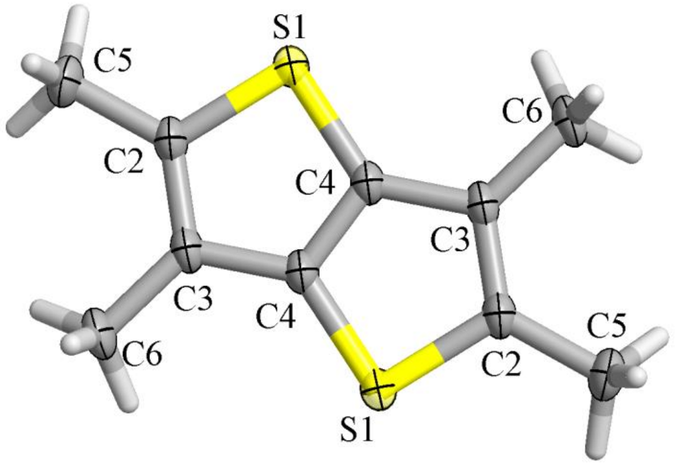

Fig. S1. X-ray crystallographic structures of **2d** with thermal ellipsoids at 50% probability.

Table S1 Crystal data and structure refinement for 2d

**Identification code**	**2d**
Empirical formula	$C_{10}H_{12}S_2$
Formula weight	98.16
Temperature/K	100.15
Crystal system	monoclinic
Space group	C2/m
a/Å	9.9600(7)
b/Å	7.0968(5)
c/Å	7.0727(5)
α/°	90
β/°	102.179(7)
γ/°	90
Volume/Å^3	488.68(6)
Z	4
ρ_{calc}g/cm^3	1.334
μ/mm^{-1}	4.439
F(000)	208.0
Crystal size/mm^3	0.13 × 0.11 × 0.09
Radiation	Cu Kα (λ = 1.54184)
2Θ range for data collection/°	12.804 to 146.01
Index ranges	-11 ≤ h ≤ 12, -8 ≤ k ≤ 8, -8 ≤ l ≤ 7
Reflections collected	1684
Independent reflections	517 [R_{int} = 0.0208, R_{sigma} = 0.0151]
Data/restraints/parameters	517/0/39
Goodness-of-fit on F^2	1.189
Final R indexes [I>=2σ (I)]	R_1 = 0.0413, wR_2 = 0.1158
Final R indexes [all data]	R_1 = 0.0419, wR_2 = 0.1164
Largest diff. peak/hole / e Å^{-3}	0.59/-0.48

Crystal structure of 2k

Experimental: Single crystals of $C_{14}H_{16}S_2$ **(2k)** were generation in the mixed solution of dichloromethane and n-hexane. A suitable crystal was selected and analyzed it on a SuperNova, Dual, Cu at zero, AtlasS2 diffractometer. The crystal was kept at 149.99(10) K during data collection. The X-ray crystallographic structures for **2k**. ORTEP representation with 50% probability thermal ellipsoids. Crystal data have been deposited to CCDC, number 2358239.

Crystal structure determination of 2k

Crystal Data for $C_{14}H_{16}S_2$ (M = 248.39 g/mol): triclinic, space group P1 (no. 1), a = 5.0343(4) Å, b = 7.6072(5) Å, c = 8.3121(8) Å, α = 96.230(7) , β = 106.369 (7), γ = 92.657(6) , V = 302.63(4) Å3, Z = 1, T = 149.99(10) K, μ(Cu Kα) = 3.704 mm-1, Dcalc = 1.363 g/cm^3, 2726 reflections measured (11.184 ≤ 2Θ ≤ 146.594), 1442 unique (Rint = 0.0201, Rsigma = 0.0218) which were used in all calculations. The final R1 was 0.0283 (I > 2σ(I)) and wR2 was 0.0804 (all data).

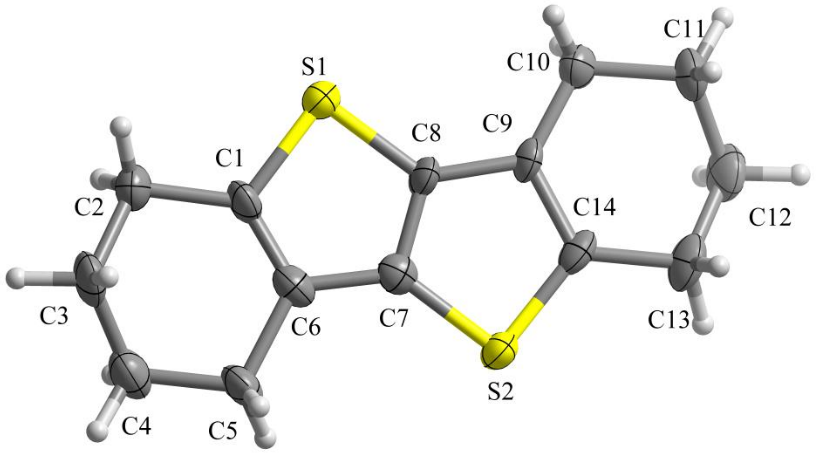

Fig. **S2**. X-ray crystallographic structures of **2k** with thermal ellipsoids at 50% probability.

Table S2. Crystal data and structure refinement for 2k.

Identification code	2k
Empirical formula	$C_{14}H_{16}S_2$
Formula weight	248.39
Temperature/K	149.99(10)
Crystal system	triclinic
Space group	P1
a/Å	5.0343(4)
b/Å	7.6072(5)
c/Å	8.3121(8)
α/°	96.230(7)
β/°	106.369(7)
γ/°	92.657(6)
Volume/Å^3	302.63(4)
Z	1
ρ_{calc}g/cm^3	1.363
μ/mm^{-1}	3.704
F(000)	132.0
Crystal size/mm^3	0.15 × 0.12 × 0.09
Radiation	Cu Kα (λ = 1.54184)
2Θ range for data collection/°	11.184 to 146.594
Index ranges	-6 ≤ h ≤ 6, -7 ≤ k ≤ 9, -10 ≤ l ≤ 8
Reflections collected	2726
Independent reflections	1442 [R_{int} = 0.0201, R_{sigma} = 0.0218]
Data/restraints/parameters	1442/132/145
Goodness-of-fit on F^2	1.137
Final R indexes [I>=2σ (I)]	R_1 = 0.0283, wR_2 = 0.0801
Final R indexes [all data]	R_1 = 0.0286, wR_2 = 0.0804
Largest diff. peak/hole / e Å^{-3}	0.23/-0.33
Flack parameter	0.04(5)

Crystal structure of 3a

Experimental: Single crystals of $C_8H_8Se_2$ (**3a**) were generation in the mixed solution of dichloromethane and n-hexane. A suitable crystal was selected and analyzed it on a SuperNova, Dual, Cu at zero, AtlasS2 diffractometer. The crystal was kept at 149.99(10) K during data collection. The X-ray crystallographic structures for **3a**. ORTEP representation with 50% probability thermal ellipsoids. Crystal data have been deposited to CCDC, number 2358237.

Crystal structure determination of 3a

Crystal Data for $C_8H_8Se_2$ (M =262.06 g/mol): monoclinic, space group P21/c (no. 14), a = 5.20270(10) Å, b = 13.3299(3) Å, c = 12.1893(2) Å, β = 93.668(2), V = 843.61(3) Å3, Z = 4, T = 149.93(11) K, μ(Cu Kα) = 10.301 mm-1, Dcalc = 2.063 g/cm^3, 5335 reflections measured (9.844 ≤ 2Θ ≤ 146.864), 1633 unique (Rint = 0.0274, Rsigma = 0.0236) which were used in all calculations. The final R1 was 0.0252 (I > 2σ(I)) and wR2 was 0.0654 (all data).

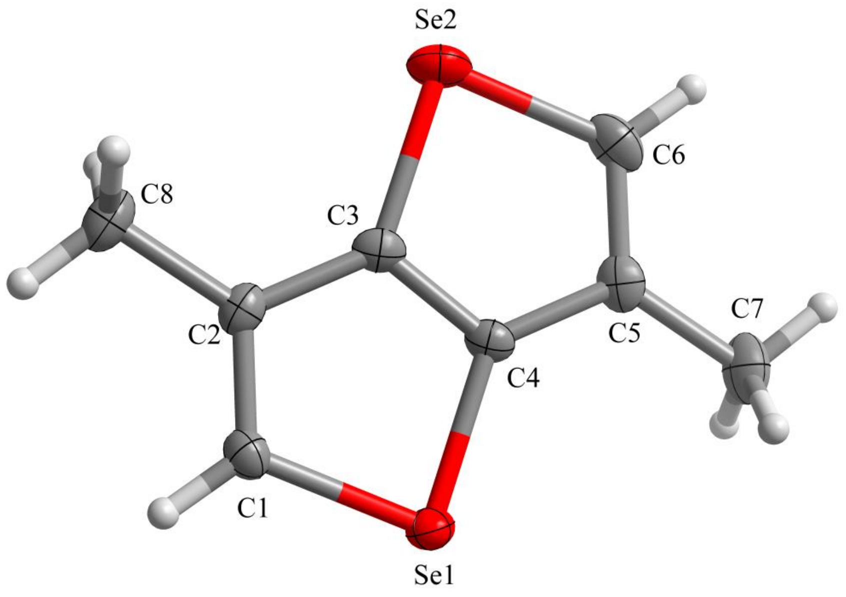

Fig. **S3**. X-ray crystallographic structures of **3a** with thermal ellipsoids at 50% probability.

Table S3. Crystal data and structure refinement for 3a

**Identification code**	**3a**
Empirical formula	$C_8H_8Se_2$
Formula weight	262.06
Temperature/K	149.93(11)
Crystal system	monoclinic
Space group	$P2_1/c$
a/Å	5.20270(10)
b/Å	13.3299(3)
c/Å	12.1893(2)
α/°	90
β/°	93.668(2)
γ/°	90
Volume/Å^3	843.61(3)
Z	4
ρ_{calc}g/cm^3	2.063
μ/mm^{-1}	10.301
F(000)	496.0
Crystal size/mm^3	0.13 × 0.11 × 0.1
Radiation	Cu Kα (λ = 1.54184)
2Θ range for data collection/°	9.844 to 146.864
Index ranges	$-6 \leq h \leq 6$, $-14 \leq k \leq 16$, $-15 \leq l \leq 15$
Reflections collected	5335
Independent reflections	1633 [R_{int} = 0.0274, R_{sigma} = 0.0236]
Data/restraints/parameters	1633/0/93
Goodness-of-fit on F^2	1.103
Final R indexes [I>=2σ (I)]	R_1 = 0.0252, wR_2 = 0.0647
Final R indexes [all data]	R_1 = 0.0263, wR_2 = 0.0654
Largest diff. peak/hole / e Å^{-3}	0.39/-0.55

D. Characterization data for all prepared compounds

5-ethyl-2-methylhept-3-yne-2,5-diol (**1b**)

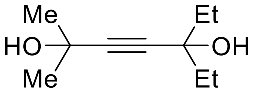

Yellow liquid (153 mg, 90% yield); R_f = 0.3 (Hexane/EtOAc = 5:1); ^{1}H NMR (400 MHz, $CDCl_3$) δ 2.55 (s, 2H), 1.65 – 1.59 (m, 4H), 1.49 (s, 6H), 0.99 (t, J = 7.5 Hz, 6H). ^{13}C NMR (100 MHz, $CDCl_3$) δ 89.2, 84.3, 71.9, 65.0, 34.1 (2C), 31.4 (2C), 8.5 (2C). ESI-HRMS (*m/z*) [M+Na]$^+$ calcd for $C_{10}H_{18}NaO_2^+$,193.1199, found: 193.1198.

2-methyl-5-propyloct-3-yne-2,5-diol (**1c**)

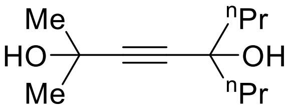

Yellow liquid (182 mg, 92% yield); R_f = 0.3 (Hexane/EtOAc = 5:1); ^{1}H NMR (400 MHz, $CDCl_3$) δ 2.01 (s, 2H), 1.66 – 1.39 (m, 14H), 0.94 (t, J = 7.2 Hz, 6H). ^{13}C NMR (100 MHz, $CDCl_3$) δ 88.9, 84.9, 70.9, 65.1, 44.2 (2C), 31.5 (2C), 17.5 (2C), 14.2 (2C). ESI-HRMS (*m/z*) [M+K]$^+$ calcd for $C_{12}H_{22}KO_2^+$, 237.1251, found: 237.1250.

1,1'-(ethyne-1,2-diyl)bis(cyclohexan-1-ol) (**1l**)

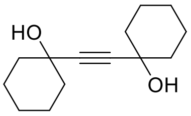

White solid (204 mg, 92% yield); MP: 106-108 ℃; R_f = 0.3 (Hexane/EtOAc = 5:1); ^{1}H NMR (400 MHz, $CDCl_3$) δ 3.66 (s, 2H), 1.92 – 1.86 (m, 4H), 1.67 – 1.62 (m, 4H), 1.57 – 1.41 (m, 10H), 1.23 – 1.10 (m, 2H). ^{13}C NMR (100 MHz, $CDCl_3$) δ 87.8 (2C), 68.5 (2C), 39.8 (4C), 25.1 (2C), 23.4 (4C). ESI-HRMS (*m/z*) [M+Na]$^+$ calcd for $C_{14}H_{22}NaO_2^+$, 245.1512, found: 245.1510.

1-((1-hydroxycyclohexyl)ethynyl)-4-propylcyclohexan-1-ol (**1m**)

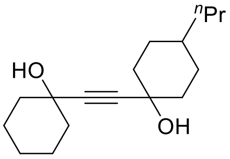

White solid (240 mg, 91% yield); MP: 112-114 ℃; R_f = 0.3 (Hexane/EtOAc = 5:1); ^{1}H NMR (400 MHz, $CDCl_3$) δ 2.46 (s, 2H), 1.93 – 1.85 (m, 4H), 1.76 – 1.43 (m, 11H), 1.37 – 1.13 (m, 8H), 0.86 (t, J = 7.2 Hz, 3H). ^{13}C NMR (100 MHz, $CDCl_3$) δ 89.4, 86.0, 68.5, 66.6, 39.9 (2C), 38.2 (3C), 35.3, 27.4 (2C), 25.1, 23.4 (2C), 20.0, 14.2. ESI-HRMS (*m/z*) [M+Na]$^+$ calcd for $C_{17}H_{28}NaO_2^+$, 287.1982, found: 287.1981.

4-(tert-butyl)-1-((1-hydroxycyclohexyl)ethynyl)cyclohexan-1-ol (**1n**)

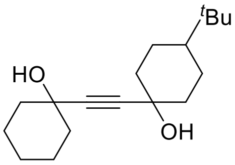

White solid (248 mg, 89% yield); MP: 151-153 °C; R_f = 0.3 (Hexane/EtOAc = 5:1); ^{1}H NMR (400 MHz, $CDCl_3$) δ 2.34 (s, 2H), 2.15 – 1.07 (m, 19H), 0.86 (s, 9H). ^{13}C NMR (100 MHz, $CDCl_3$) δ 88.7, 87.4, 69.4, 68.7, 46.9, 40.3 (2C), 40.0 (2C), 32.2, 27.5 (3C), 25.1, 24.8 (2C), 23.5 (2C). ESI-HRMS (*m/z*) $[M+Na]^+$ calcd for $C_{18}H_{30}NaO_2^+$, 301.2138, found: 301.2138.

2-methyl-5-phenyloct-3-yne-2,5-diol (**1r**)

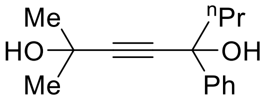

Yellow liquid (125 mg, 54% yield); R_f = 0.3 (Hexane/EtOAc = 5:1); ^{1}H NMR (400 MHz, $CDCl_3$) δ 7.62 – 7.57 (m, 2H), 7.36 – 7.26 (m, 3H), 2.52 (s, 2H), 1.95 – 1.76 (m, 2H), 1.57 (s, 6H), 1.38 – 1.22 (m, 2H), 0.88 (t, *J* = 7.4 Hz, 3H). ^{13}C NMR (100 MHz, $CDCl_3$) δ 144.7, 128.1 (2C), 127.5, 125.3 (2C), 90.7, 84.5, 73.1, 65.2, 47.5, 31.4 (2C), 18.0, 13.9. ESI-HRMS (*m/z*) $[M+Na]^+$ calcd for $C_{15}H_{20}NaO_2^+$, 255.1356, found: 255.1354.

3,6-dimethylthieno[3,2-b]thiophene (**2a**) [2]

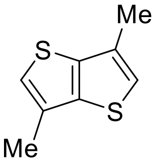

Yellow solid (68 mg, 81% yield); R_f = 0.7 (Hexane); ^{1}H NMR (500 MHz, $CDCl_3$) δ 6.96 (s, 2H), 2.36 (s, 6H). ^{13}C NMR (125 MHz, $CDCl_3$) δ 140.0 (2C), 130.3 (2C), 121.8 (2C), 14.6 (2C). ESI-HRMS (*m/z*) $[M+H]^+$ calcd for $C_8H_9S_2^+$, 169.0140, found: 169.0138.

3-ethyl-2,6-dimethylthieno[3,2-b]thiophene (**2b**)

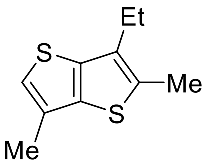

Yellow liquid (71 mg, 72% yield); R_f = 0.7 (Hexane); ^{1}H NMR (400 MHz, $CDCl_3$) δ 6.87 (s, 1H), 2.69 (q, *J* = 7.6 Hz, 2H), 2.48 (s, 3H), 2.34 (s, 3H), 1.27 (t, *J* = 7.6 Hz, 3H). ^{13}C NMR (100 MHz, $CDCl_3$) δ 139.3, 136.1, 133.9, 132.2, 129.9, 119.7, 20.9, 14.7, 14.1, 13.4. ESI-HRMS (*m/z*) $[M]^+$ calcd for $C_{10}H_{12}S_2^+$, 196.0374, found: 196.0368.

2-ethyl-6-methyl-3-propylthieno[3,2-b]thiophene (**2c**)

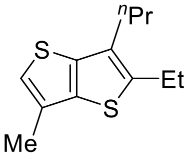

Brown liquid (66 mg, 59% yield); R_f = 0.7 (Hexane); ^{1}H NMR (400 MHz, $CDCl_3$) δ 6.87 (s, 1H), 2.86 (q, *J* = 7.5 Hz, 2H), 2.70 – 2.62 (m, 2H), 2.34 (s, 3H), 1.82 – 1.66 (m, 2H), 1.32 (t, *J* = 7.5 Hz, 3H),

0.98 (t, J = 7.4 Hz, 3H). ^{13}C NMR (100 MHz, $CDCl_3$) δ 142.5, 130.0, 129.9, 119.1, 29.6, 22.4, 22.3, 16.4, 14.7, 14.0. ESI-HRMS (*m/z*) $[M]^+$ calcd for $C_{12}H_{16}S_2^+$, 224.0688, found: 224.0679.

2,3,5,6-tetramethylthieno[3,2-b]thiophene (**2d**)

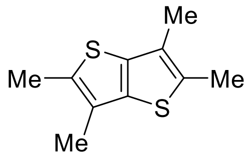

Yellow solid (77 mg, 79% yield); R_f = 0.7 (Hexane); MP: 136-137 ℃. ^{1}H NMR (500 MHz, $CDCl_3$) δ 2.45 (s, 6H), 2.21 (s, 6H). ^{13}C NMR (125 MHz, $CDCl_3$) δ 136.1 (2C), 132.0 (2C), 125.3 (2C), 14.0 (2C), 12.5 (2C). ESI-HRMS (*m/z*) $[M+K]^+$ calcd for $C_{10}H_{12}KS_2^+$, 235.0012, found: 235.0022.

2-ethyl-5,6-dimethyl-3-propylthieno[3,2-b]thiophene (**2e**)

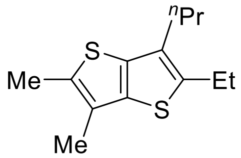

Brown liquid (87 mg, 73% yield); R_f = 0.7 (Hexane); ^{1}H NMR (400 MHz, $CDCl_3$) δ 2.83 (q, J = 7.5 Hz, 2H), 2.62 – 2.59 (m, 2H), 2.43 (s, 3H), 2.20 (s, 3H), 1.70 (q, J = 7.5 Hz, 2H), 1.30 (t, J = 7.5 Hz, 3H), 0.96 (t, J = 7.3 Hz, 3H). ^{13}C NMR (100 MHz, $CDCl_3$) δ 140.3, 136.7, 135.1, 132.2, 129.5, 125.38, 29.7, 22.3, 22.2, 16.5, 14.0, 14.0, 12.5. ESI-HRMS (*m/z*) $[M+H]^+$ calcd for $C_{13}H_{19}S_2^+$, 239.0923, found: 239.0921.

2,5-diethyl-3,6-dimethylthieno[3,2-b]thiophene (**2f**)

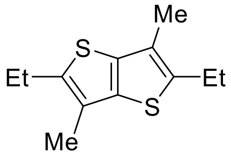

Yellow solid (83 mg, 74% yield); R_f = 0.7 (Hexane); MP: 79-81 ℃.^{1}H NMR (500 MHz, $CDCl_3$) δ 2.83 (q, J = 7.5 Hz, 4H), 2.23 (s, 6H), 1.30 (t, J = 7.6 Hz, 6H). ^{13}C NMR (125 MHz, $CDCl_3$) δ 139.9 (2C), 136.2 (2C), 124.5 (2C), 22.3 (2C), 16.0 (2C), 12.4 (2C). ESI-HRMS (*m/z*) $[M+H]^+$ calcd for $C_{12}H_{17}S_2^+$, 225.0766, found: 225.0766.

2,6-diethyl-5-methyl-3-propylthieno[3,2-b]thiophene (**2g**)

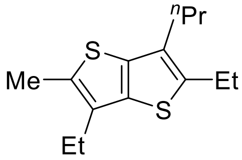

Brown liquid (79 mg, 63% yield); R_f = 0.7 (Hexane); ^{1}H NMR (400 MHz, $CDCl_3$) δ 2.83 (q, J = 7.5 Hz, 2H), 2.68 – 2.58 (m, 4H), 2.44 (s, 3H), 1.75 – 1.63 (m, 2H), 1.31 – 1.23 (m, 6H), 0.96 (t, J = 7.4 Hz, 3H). ^{13}C NMR (100 MHz, $CDCl_3$) δ 140.3, 135.7, 135.5, 131.8, 131.7, 129.4, 29.7, 22.3, 22.1, 20.9, 16.4, 14.0, 13.8, 13.4. ESI-HRMS (*m/z*) $[M+H]^+$ calcd for $C_{14}H_{21}S_2^+$, 253.1079, found: 253.1077.

2,5-diisopropyl-3,6-dimethylthieno[3,2-b]thiophene (**2h**)

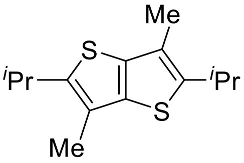

Yellow solid (93 mg, 74% yield); R_f = 0.7 (Hexane); MP: 141-142 ℃. ^{1}H NMR (500 MHz, $CDCl_3$) δ 3.35 – 3.29 (m, 2H), 2.25 (s, 3H), 1.33 (s, 6H), 1.32 (s, 6H). ^{13}C NMR (125 MHz, $CDCl_3$) δ 146.1 (2C), 135.9 (2C), 123.7 (2C), 28.9 (2C), 24.5 (4C), 12.6 (2C). ESI-HRMS (*m/z*) $[M+H]^+$ calcd for $C_{14}H_{21}S_2^+$, 253.1079, found: 253.1077.

2,5-diethyl-3,6-dipropylthieno[3,2-b]thiophene (**2i**)

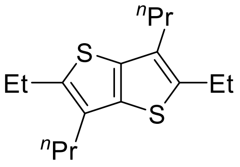

Brown liquid (101 mg, 72% yield); R_f = 0.7 (Hexane); ^{1}H NMR (500 MHz, $CDCl_3$) δ 2.83 (q, J = 7.5 Hz, 4H), 2.64 – 2.58 (m, 4H), 1.76 – 1.64 (m, 4H), 1.29 (t, J = 7.5 Hz, 6H), 0.97 (t, *J* = 7.4 Hz, 6H). ^{13}C NMR (125 MHz, $CDCl_3$) δ 140.4 (2C), 135.7 (2C), 129.5 (2C), 29.7 (2C), 22.3 (2C), 22.2 (2C), 16.5 (2C), 14.1 (2C). ESI-HRMS (*m/z*) $[M+H]^+$ calcd for $C_{16}H_{25}S_2^+$, 281.1392, found: 281.1391.

2-ethyl-5-isopropyl-6-methyl-3-propylthieno[3,2-b]thiophene (**2j**)

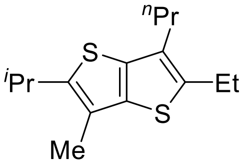

Brown liquid (92 mg, 69% yield); R_f = 0.7 (Hexane); ^{1}H NMR (500 MHz, $CDCl_3$) δ 3.36 – 3.22 (m, 1H), 2.83 (q, *J* = 7.5 Hz, 2H), 2.64 – 2.60 (m, 2H), 2.23 (s, 3H), 1.71 (q, *J* = 7.5 Hz, 2H), 1.33 – 1.27 (m, 9H), 0.98 (t, *J* = 7.3 Hz, 3H). ^{13}C NMR (125 MHz, $CDCl_3$) δ 146.1, 140.4, 136.7, 135.0, 129.8, 123.4, 29.8, 28.9, 24.5 (2C), 22.3, 22.2, 16.6, 14.1, 12.6. ESI-HRMS (*m/z*) $[M+H]^+$ calcd for $C_{15}H_{23}S_2^+$, 267.1236, found: 267.1233.

1,2,3,4,6,7,8,9-octahydrobenzo[b]benzo[4,5]thieno[2,3-d]thiophene (**2k**)

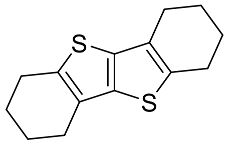

Yellow solid (77 mg, 62% yield); R_f = 0.7 (Hexane); MP: 126-128 ℃. ^{1}H NMR (400 MHz, $CDCl_3$) δ 2.88 – 2.85 (m, 4H), 2.67 – 2.63 (m, 4H), 1.97 – 1.82 (m, 8H). ^{13}C NMR (100 MHz, $CDCl_3$) δ 135.3 (2C), 134.9 (2C), 127.8 (2C), 26.0 (2C), 24.6 (2C), 23.5 (2C), 22.5 (2C). ESI-HRMS (*m/z*) $[M+H]^+$ calcd for $C_{14}H_{17}S_2^+$, 249.0766, found: 249.0767.

2-propyl-1,2,3,4,6,7,8,9-octahydrobenzo[b]benzo[4,5]thieno[2,3-d]thiophene (**2l**)

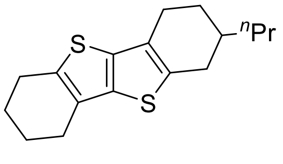

Yellow solid (103 mg, 71% yield); R_f = 0.7 (Hexane); MP: 99-101 ℃. ^{1}H NMR (400 MHz, $CDCl_3$) δ 2.95-2.90 (m, 1H), 2.86-2.82 (m, 2H), 2.72-2.66 (m, 1H), 2.64-2.28 (m, 3H), 2.52-2.44 (m, 1H), 2.00 – 1.95 (m, 1H), 1.92 – 1.83 (m, 5H),1.52-1.37 (m, 5H), 0.94

(t, J = 6.4 Hz, 3H). ^{13}C NMR (100 MHz, $CDCl_3$) δ 135.3, 135.2, 127.9, 127.8, 38.1, 34.6, 32.3, 28.9, 26.0, 24.6, 24.3, 23.5, 22.5, 20.1, 14.2. ESI-HRMS (*m/z*) $[M+H]^+$ calcd for $C_{17}H_{23}S_2^+$, 291.1235, found: 291.1233.

2-(tert-butyl)-1,2,3,4,6,7,8,9-octahydrobenzo[b]benzo[4,5]thieno[2,3-d]thiophene (**2m**)

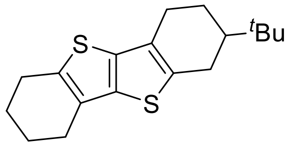

Yellow solid (112 mg, 74% yield); R_f = 0.7 (Hexane); MP: 136-138 ℃. ^{1}H NMR (400 MHz, $CDCl_3$) δ 2.92-2.84 (m, 3H), 2.78-2.72 (m, 1H), 2.65-2.54 (m, 4H), 2.09-2.05 (m, 1H), 1.92-1.85 (m, 4H), 1.64-1.56 (m, 1H), 1.49-1.38 (m, 1H), 0.98 (s, 9H). ^{13}C NMR (100 MHz, $CDCl_3$) δ 136.2, 135.3, 135.2, 134.5, 127.9, 127.9, 45.5, 32.5, 27.6, 27.3 (3C), 26.0, 25.5, 24.6, 24.1, 23.5, 22.5. ESI-HRMS (*m/z*) $[M+H]^+$ calcd for $C_{18}H_{25}S_2^+$, 305.1392, found: 305.1391.

3-methyl-6-(thiophen-2-yl)thieno[3,2-b]thiophene (**2n**)

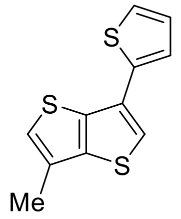

Black liquid (86 mg, 73% yield); R_f = 0.7 (Hexane); ^{1}H NMR (400 MHz, $CDCl_3$) δ 7.47 (s, 1H), 7.39 – 7.38 (m, 1H), 7.29 – 7.28 (m, 1H), 7.13 – 7.10 (m, 1H), 7.06 (s, 1H), 2.40 (s, 3H). ^{13}C NMR (100 MHz, $CDCl_3$) δ 140.8, 137.5, 136.6, 130.0, 128.7, 127.7, 124.2, 123.8, 122.6, 121.0, 14.6. ESI-HRMS (*m/z*) $[M+H]^+$ calcd for $C_{11}H_9S_3^+$, 236.9861, found: 236.9861.

2-methyl-5-(6-methylthieno[3,2-b]thiophen-3-yl)furan (**2o**)

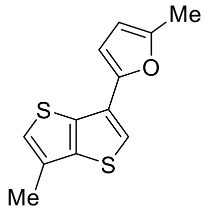

Yellow solid (66 mg, 56% yield); R_f = 0.7 (Hexane); MP: 114-116 ℃. ^{1}H NMR (400 MHz, $CDCl_3$) δ 7.49 (s, 1H), 7.04 (s, 1H), 6.51 (s, 1H), 6.09 (s, 1H), 2.39 (s, 6H). ^{13}C NMR (100 MHz, $CDCl_3$) δ 151.7, 147.8, 140.6, 129.8, 125.6, 122.5, 118.9, 107.4, 106.8, 14.6, 13.6. ESI-HRMS (*m/z*) $[M+H]^+$ calcd for $C_{12}H_{11}OS_2^+$, 235.0246, found: 235.0247.

3-methyl-6-(p-tolyl)thieno[3,2-b]thiophene (**2p**)

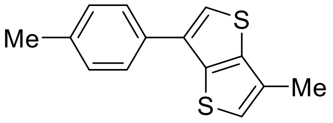

Brown solid (73 mg, 68% yield); R_f = 0.5 (Hexane); MP: 135-137 ℃. ^{1}H NMR (400 MHz, $CDCl_3$) δ 7.66 (s, 1H),

7.64 (s, 1H), 7.44 (s, 1H), 7.27 (d, J = 8.0 Hz, 2H), 7.04 (s, 1H), 2.40 (s, 6H). ^{13}C NMR (125 MHz, $CDCl_3$) δ 140.9, 137.4, 137.1, 135.1, 132.0, 130.0, 129.6 (2C), 126.3 (2C), 122.2, 121.1, 21.2, 14.6. ESI-HRMS (*m/z*) $[M+H]^+$ calcd for $C_{14}H_{13}S_2^+$, 213.0732, found: 213.0733.

2-ethyl-6-methyl-3-phenylthieno[3,2-b]thiophene (**2q**)

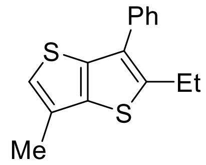

Yellow liquid (72 mg, 56% yield); R_f = 0.5 (Hexane); ^{1}H NMR (400 MHz, $CDCl_3$) δ 7.56 – 7.34 (m, 5H), 6.90 (s, 1H), 2.97 (q, J = 7.5 Hz, 2H), 2.38 (s, 3H), 1.33 (t, J = 7.5 Hz, 3H). ^{13}C NMR (100 MHz, $CDCl_3$) δ 144.1, 139.7, 136.3, 135.3, 130.8, 130.0, 128.7 (2C), 128.6 (2C), 127.4, 120.4, 23.0, 16.7, 14.7. ESI-HRMS (*m/z*) $[M+H]^+$ calcd for $C_{15}H_{15}S_2^+$, 259.0610, found: 259.0609.

3-(4-methoxyphenyl)-6-phenylthieno[3,2-b]thiophene (**2r**)

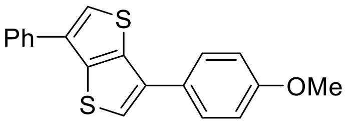

Yellow solid (111 mg, 69% yield); R_f = 0.5 (Hexane); MP: 143-145 ℃. ^{1}H NMR (400 MHz, $CDCl_3$) δ 7.89 – 7.33 (m, 9H), 7.05 – 6.99 (m, 2H), 3.88 (s, 3H). ^{13}C NMR (100 MHz, $CDCl_3$) δ 159.2, 138.2, 138.1, 134.9, 134.6, 134.5, 128.9 (2C), 127.72 (2C), 127.70, 127.4, 126.5 (2C), 122.2, 120.9, 114.4 (2C), 55.3. ESI-HRMS (*m/z*) $[M+H]^+$ calcd for $C_{19}H_{15}OS_2^+$, 323.0559, found: 323.0556.

3-benzyl-6-ethyl-2-phenylthieno[3,2-b]thiophene (**2s**)

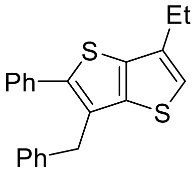

Yellow liquid (132 mg, 79% yield); R_f = 0.5 (Hexane); ^{1}H NMR (400 MHz, $CDCl_3$) δ 7.52 – 7.13 (m, 10H), 6.89 (s, 1H), 4.12 (s, 2H), 2.84 – 2.77 (m, 2H), 1.28 – 1.24 (m, 3H). ^{13}C NMR (125 MHz, $CDCl_3$) δ 149.4, 139.1, 139.1, 138.94, 136.1, 134.7, 129.3 (2C), 128.6 (2C), 128.6 (2C), 128.4 (2C), 128.2, 127.6, 126.3, 115.6, 34.2, 24.3, 15.7. ESI-HRMS (*m/z*) $[M+H]^+$ calcd for $C_{21}H_{19}S_2^+$, 335.0923, found: 335.0919.

3,6-dibenzyl-2-phenylthieno[3,2-b]thiophene (**2t**)

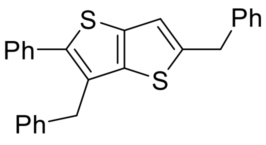

Yellow liquid (145 mg, 77% yield); R_f = 0.5 (Hexane); ^{1}H NMR (500 MHz, $CDCl_3$) δ 7.51 – 7.27 (m, 9H), 7.25 – 7.15 (m, 6H), 6.91 (s, 1H), 4.14 (s, 2H), 4.11 (s, 2H). ^{13}C NMR (125 MHz, $CDCl_3$) δ 145.9, 140.1, 139.7, 139.5, 138.8, 136.1, 133.6, 134.6, 129.3

(2C), 128.6 (2C), 128.6 (2C), 128.5 (2C), 128.4 (2C), 128.2, 127.7 (2C), 126.6, 126.3, 117.5, 37.1, 34.1. ESI-HRMS (*m/z*) $[M]^+$ calcd for $C_{26}H_{20}S_2^+$, 396.1001, found: 396.0998.

3,6-dimethylselenopheno[3,2-b]selenophene (**3a**)

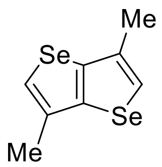

Yellow solid (87 mg, 69% yield); R_f = 0.7 (Hexane); MP: 96-98 ℃. ^{1}H NMR (400 MHz, $CDCl_3$) δ 7.47 (s, 2H), 2.37 (s, 6H). ^{13}C NMR (100 MHz, $CDCl_3$) δ 141.6 (2C), 135.2 (2C), 123.6 (2C), 17.2 (2C). ESI-HRMS (*m/z*) $[M+H]^+$ calcd for $C_8H_9Se_2^+$, 252.9148, found: 252.9158.

2,3,5,6-tetramethylselenopheno[3,2-b]selenophene (**3b**)

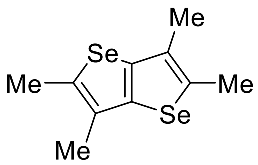

Brown solid (85 mg, 64% yield); R_f = 0.7 (Hexane); MP: 101-103 ℃. ^{1}H NMR (400 MHz, $CDCl_3$) δ 2.51 (s, 6H), 2.16 (s, 6H). ^{13}C NMR (100 MHz, $CDCl_3$) δ 137.6 (2C), 135.8 (2C), 130.2 (2C), 16.2 (2C), 14.5 (2C). ESI-HRMS (*m/z*) $[M+H]^+$ calcd for $C_{10}H_{13}Se_2^+$, 280.9461, found: 280.9459.

2,5-diiodo-3,6-dimethylthieno[3,2-b]thiophene (**4a**) [3]

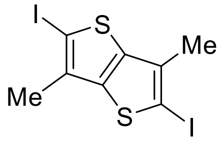

White solid (512 mg, 61% yield); R_f = 0.7 (Hexane); ^{1}H NMR (400 MHz, $CDCl_3$) δ 2.26 (s, 1H). ^{13}C NMR (100 MHz, $CDCl_3$) δ 140.9 (2C), 134.2 (2C), 76.0 (2C), 16.9 (2C).

((3,6-dimethylthieno[3,2-b]thiophene-2,5-diyl)bis(ethyne-2,1-diyl))bis(trimethylsilane) (**4b**)

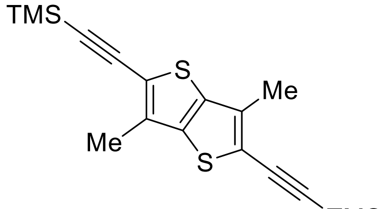

Yellow solid (67 mg, 78% yield); MP:157-158 ℃. R_f = 0.7 (Hexane); ^{1}H NMR (400 MHz, $CDCl_3$) δ 2.35 (s, 6H), 0.27 (s, 18H). ^{13}C NMR (100 MHz, $CDCl_3$) δ 138.0 (2C), 135.7 (2C), 121.2 (2C), 103.5 (2C), 97.6 (2C), 14.0 (2C), 0.0 (6C). ESI-HRMS (*m/z*) $[M+Na]^+$ calcd for $C_{18}H_{24}NaS_2Si_2^+$, 383.0750, found: 383.0752.

2,5-diethynyl-3,6-dimethylthieno[3,2-b]thiophene (**4c**)

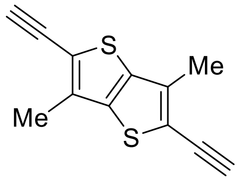

White solid (46 mg, 92% yield); R_f = 0.5 (Hexane); MP:123-124 ℃. ^{1}H NMR (400 MHz, $CDCl_3$) δ 3.62 (s, 2H), 2.39 (s, 6H). ^{13}C NMR (100 MHz, $CDCl_3$) δ 138.1 (2C), 136.2 (2C), 120.2 (2C), 85.5 (4C), 13.9 (2C). ESI-HRMS (*m/z*) $[M+Na]^+$ calcd for $C_{24}H_{16}NaS_2^+$, 238.9960, found: 238.9951.

3,6-dimethyl-2,5-bis(phenylethynyl)thieno[3,2-b]thiophene (**4d**)

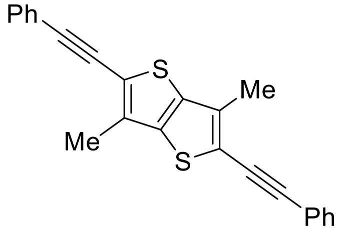

Yellow solid (49 mg, 56% yield); R_f = 0.7 (Hexane); MP:137-139 ℃. ^{1}H NMR (400 MHz, $CDCl_3$) δ 7.57 – 7.52 (m, 4H), 7.38 – 7.34 (m, 6H), 2.46 (s, 6H). ^{13}C NMR (100 MHz, $CDCl_3$) δ 138.5 (2C), 134.9 (2C), 131.3 (4C), 128.4 (2C), 128.4 (4C), 122.9 (2C), 121.0 (2C), 97.5 (2C), 82.9 (2C), 14.1 (2C). ESI-HRMS (*m/z*) $[M+Na]^+$ calcd for $C_{24}H_{16}NaS_2^+$, 391.0586, found: 391.0580.

3,6-dimethyl-2,5-diphenylthieno[3,2-b]thiophene (**4e**)

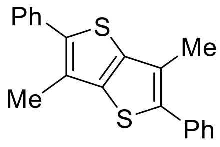

Yellow solid (67 mg, 68% yield); R_f = 0.7 (Hexane); MP:168-170 ℃. ^{1}H NMR (400 MHz, $CDCl_3$) δ 7.53 (d, J = 7.3 Hz, 4H), 7.45 (t, J = 7.6 Hz, 4H), 7.36 (d, J = 7.9 Hz, 2H), 2.44 (s, 6H). ^{13}C NMR (100 MHz, $CDCl_3$) δ 139.1 (2C), 138.1 (2C), 135.1 (2C), 129.1 (4C), 128.6 (4C), 127.4 (2C), 125.8 (2C), 14.0 (2C). ESI-HRMS (*m/z*) $[M+H]^+$ calcd for $C_{20}H_{17}S_2^+$, 321.0766, found: 321.0761.

2,5-diiodo-2,5-dimethylhex-3-yne (**5b**)

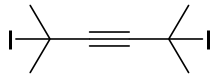

Yellow liquid (141 mg, 78% yield); R_f = 0.6 (Hexane/EtOAc = 30:1); ^{1}H NMR (400 MHz, $CDCl_3$) δ 1.36 (s, 12H). ^{13}C NMR (100 MHz, $CDCl_3$) δ 113.0 (2C), 91.2 (2C), 28.9 (42C). ESI-HRMS (*m/z*) $[M+Na]^+$ calcd for $C_8H_{12}NaI_2^+$, 384.8921, found: 384.8926.

2,4-diphenylthiophene (**5d**) [4]

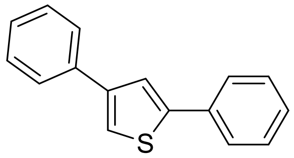

Yellow solid (20.6 mg, 35% yield); R_f = 0.5 (Hexane); ^{1}H NMR (400 MHz, $CDCl_3$) δ 7.67 – 7.62 (m, 4H), 7.60 (s, 1H), 7.44 – 7.39 (m, 5H), 7.33-7.31(m, 2H); ^{13}C NMR (100

MHz, $CDCl_3$) δ 145.0, 143.1, 135.9, 134.3, 128.9 (2C), 128.8 (2C), 127.7, 127.3, 126.3 (2C), 125.8 (2C), 122.3, 119.7. ESI-HRMS (*m/z*) $[M+H]^+$ calcd for $C_{16}H_{13}S^+$, 237.0732, found: 237.0734.

3,6-dimethylthieno[3,2-b]thiophene-2,5-d2 (**2a-D**)

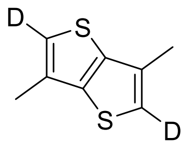

Yellow solid (68 mg, 81% yield); R_f = 0.7 (Hexane); ^1H NMR (400 MHz, $CDCl_3$) δ 6.96 (s, 1.68H), 2.36 (s, 6H). ^{13}C NMR (100 MHz, $CDCl_3$) δ 140.0 (2C), 130.3 (2C), 121.8 (2C), 14.6 (2C). ^2H NMR (77 MHz, CH_2Cl_2) δ 6.96 (s, 2D).

References:

[1] Princival, J. L.; Ferreira, J. G. $CeCl_3$-mediated Addition of Acetylenic Bis-lithium Salts to Aldehydes and Ketones: an Efficient route to Bis-substituted Alkyne Diols, *Tetrahedron Letters*, **2017**, *58*, 3525-3528.

[2] Kong, H.; Jung, Y. K.; Cho, N. S.; Kang, I.; Park, J.; Cho, S.; Shim, H. New Semiconducting Polymers Containing 3,6-Dimethyl (thieno[3,2-b]-thiophene or Selenopheno[3,2-b]selenophene) for Organic Thin-Film Transistors, *Chem Mater.*, **2009**, *21*, 2650–2660.

[3] Sato, M.; Kubota, Y.; Tanemura, A.; Maruyama, G.; Fujihara, T.; Nakayama, J.; Takayanagi, T.; Takahashi, K.; Unoura K. Synthesis and Some Properties of Bis(ruthenocenyl)thiophene Derivatives Possible Spin-Coupling in the Two-Electron Oxidized Species of Dinuclear Ruthenocenes Bridged by Thiophene Derivatives. *Eur. J. Inorg. Chem.*, **2006**, *22*, 4577–4588.

[4] Huang, G.; Li, J.; Li, J.; Li, J.; Sun, M.; Zhou, P.; Chen, L.; Huang, Y.; Jiang, S.; Yibiao Li, Y. Access to Substituted Thiophenes through Xanthate Mediated Vinyl C(sp^2)-Br Bond Cleavage and Heterocyclization of Bromoenynes, *J. Org. Chem*, **2020**, *85*, 13037-13049.

E. ^{1}H NMR and ^{13}C NMR spectra for products

^{1}H NMR (400 MHz, $CDCl_3$) spectrum of compound 1a

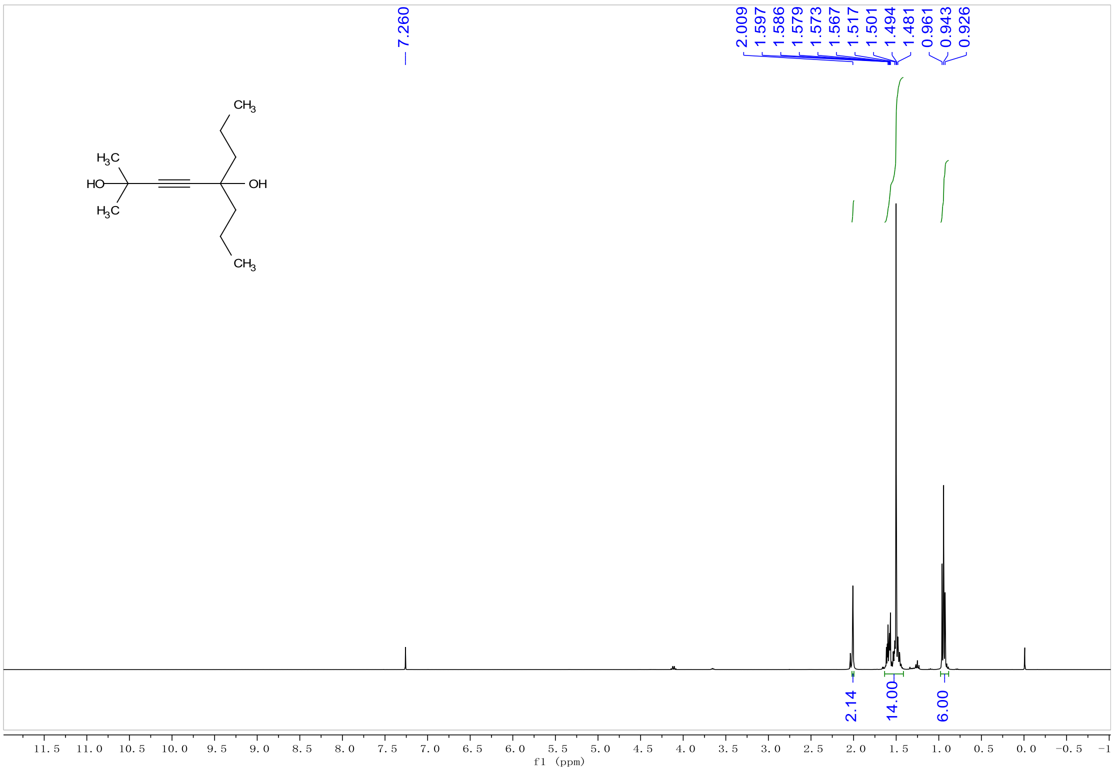

^{13}C NMR (100 MHz, $CDCl_3$) spectrum of compound 1a

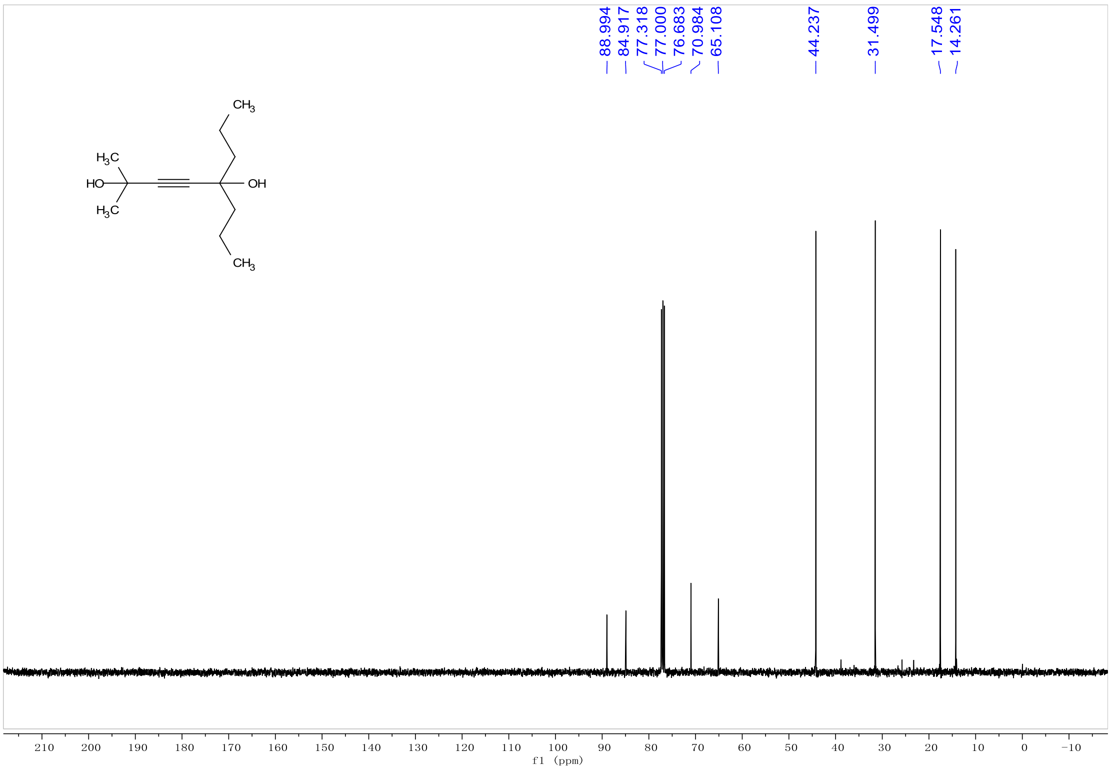

^{1}H NMR (400 MHz, $CDCl_3$) spectrum of compound 1b

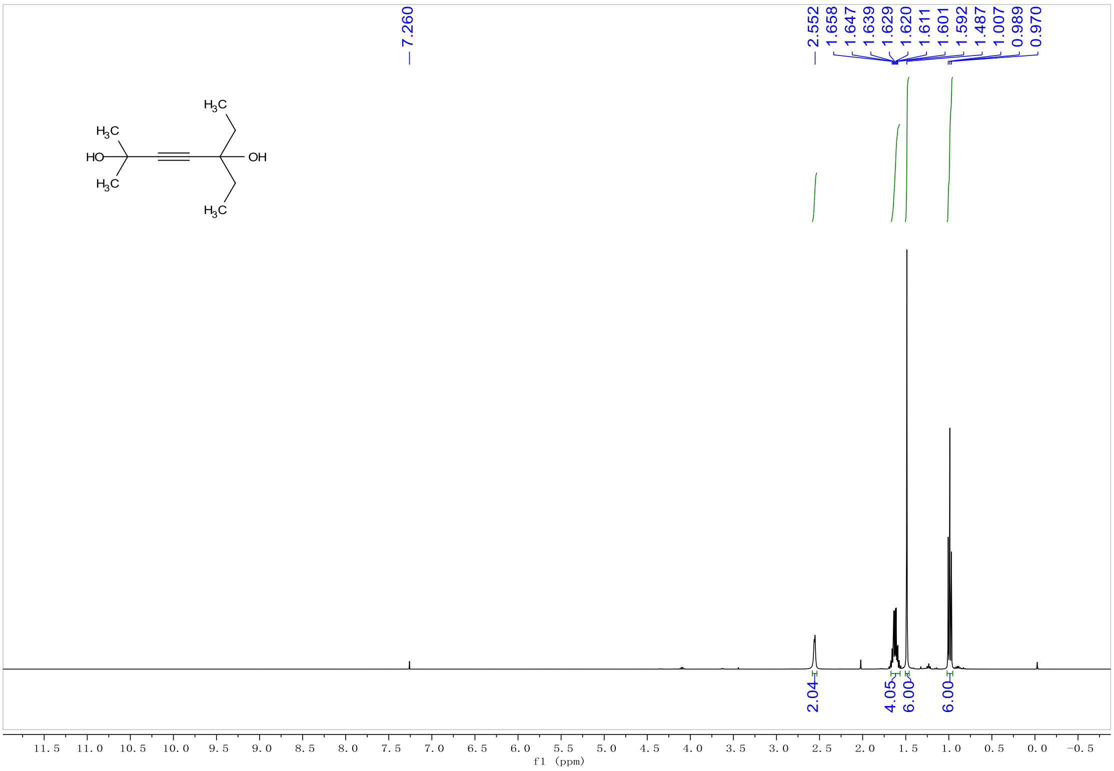

^{13}C NMR (100 MHz, $CDCl_3$) spectrum of compound 1b

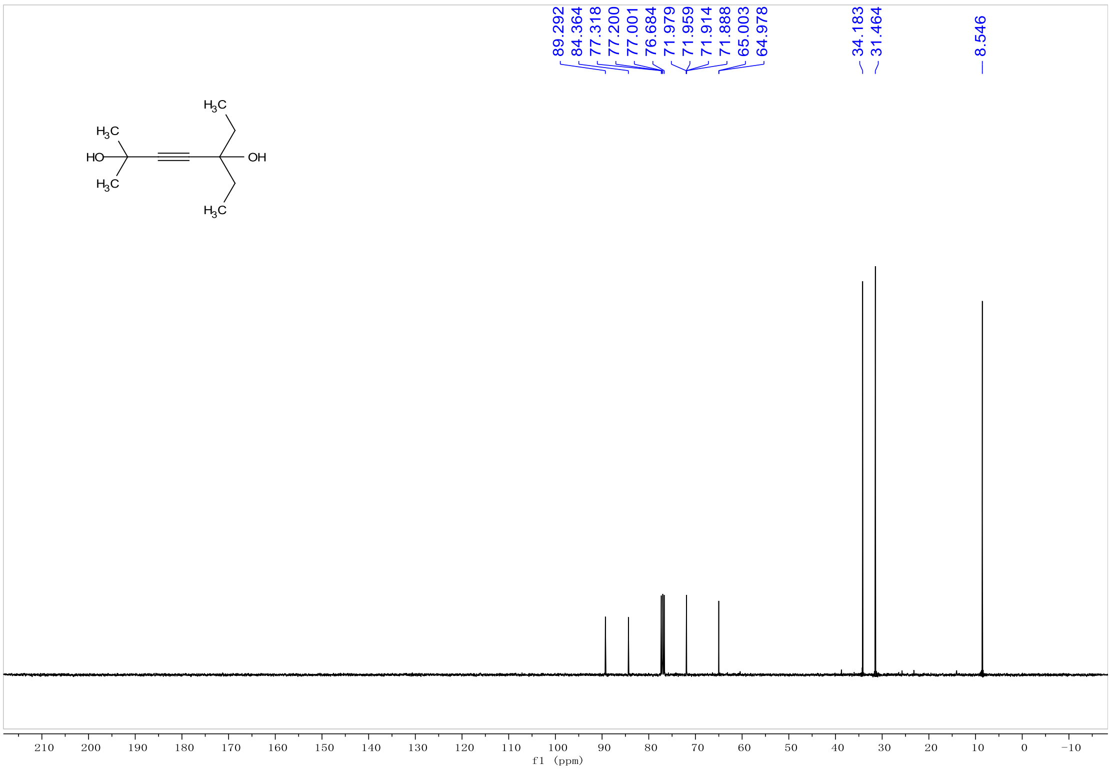

^{1}H NMR (400 MHz, $CDCl_3$) spectrum of compound 1c

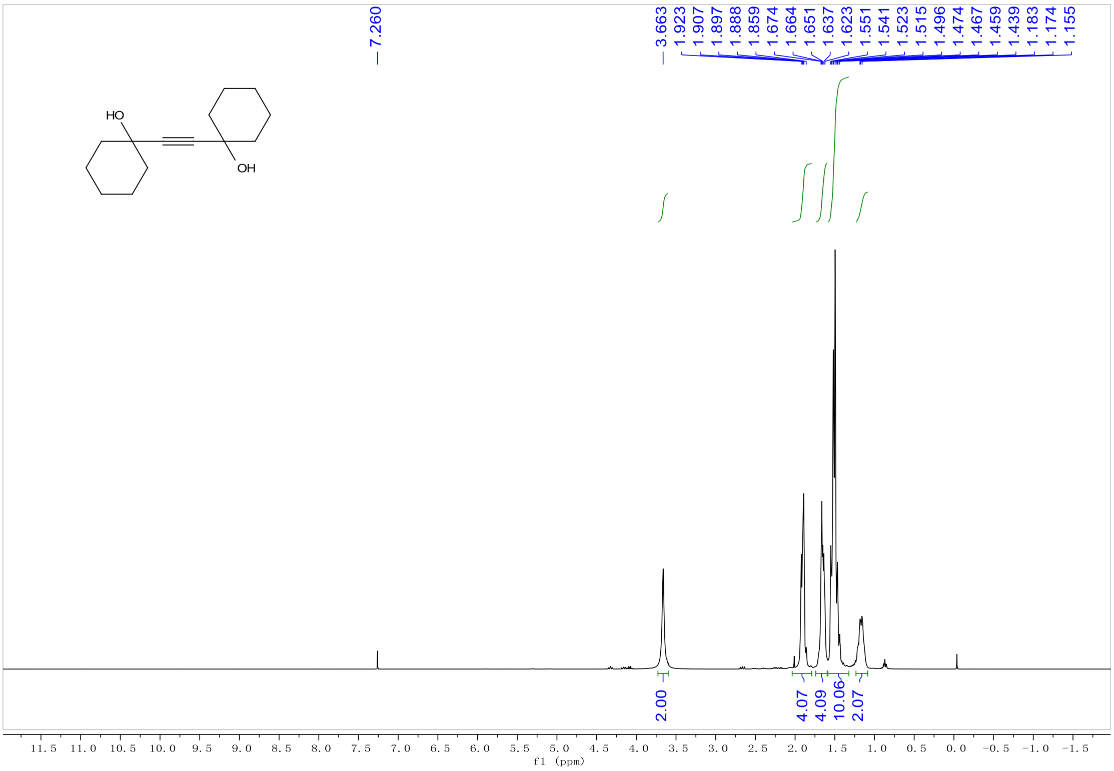

^{13}C NMR (100 MHz, $CDCl_3$) spectrum of compound 1c

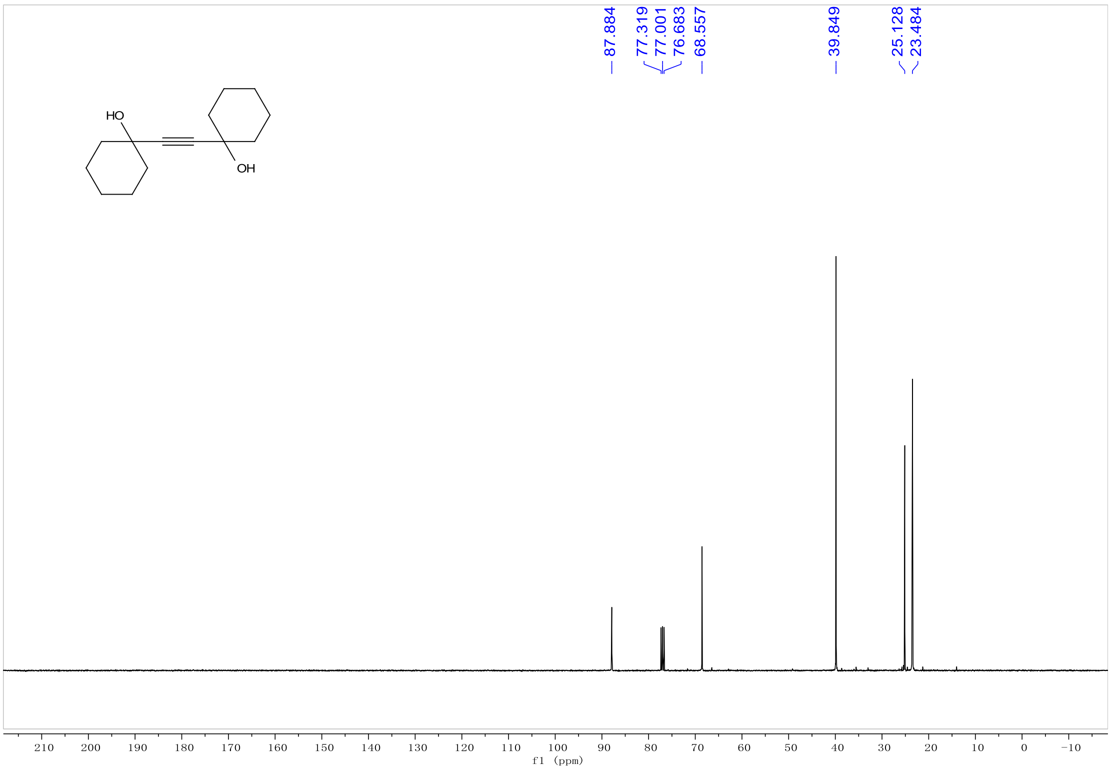

^{1}H NMR (400 MHz, $CDCl_3$) spectrum of compound 1d

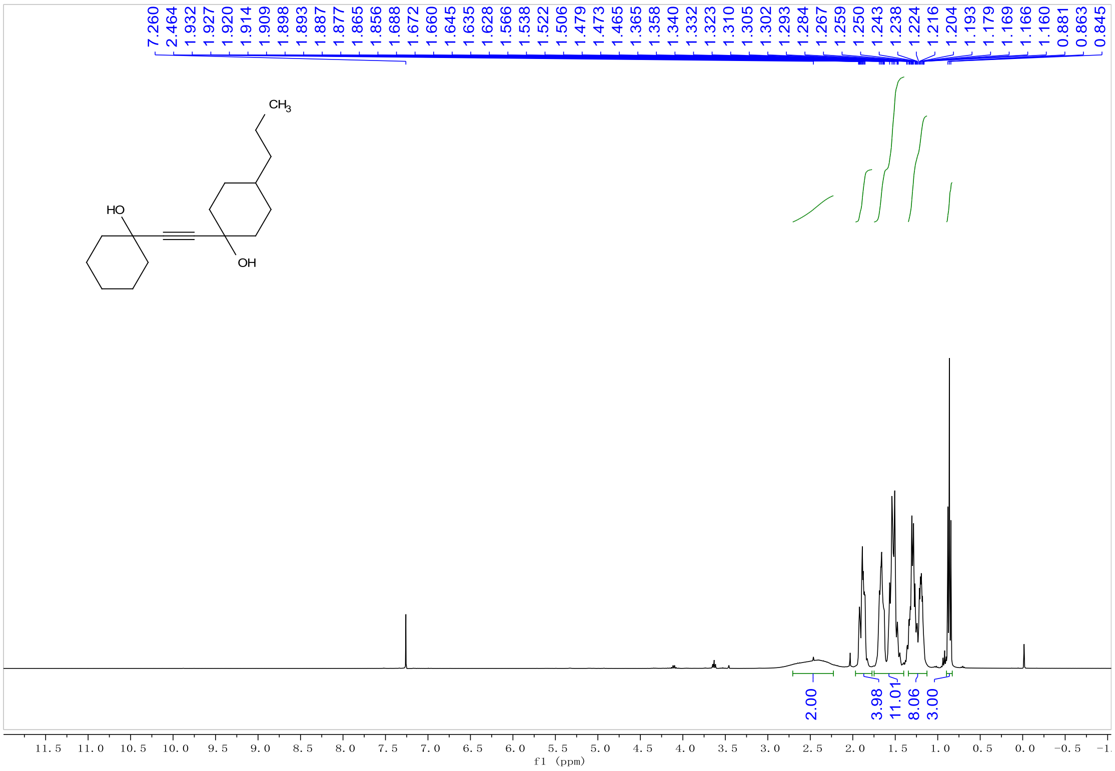

^{13}C NMR (100 MHz, $CDCl_3$) spectrum of compound 1d

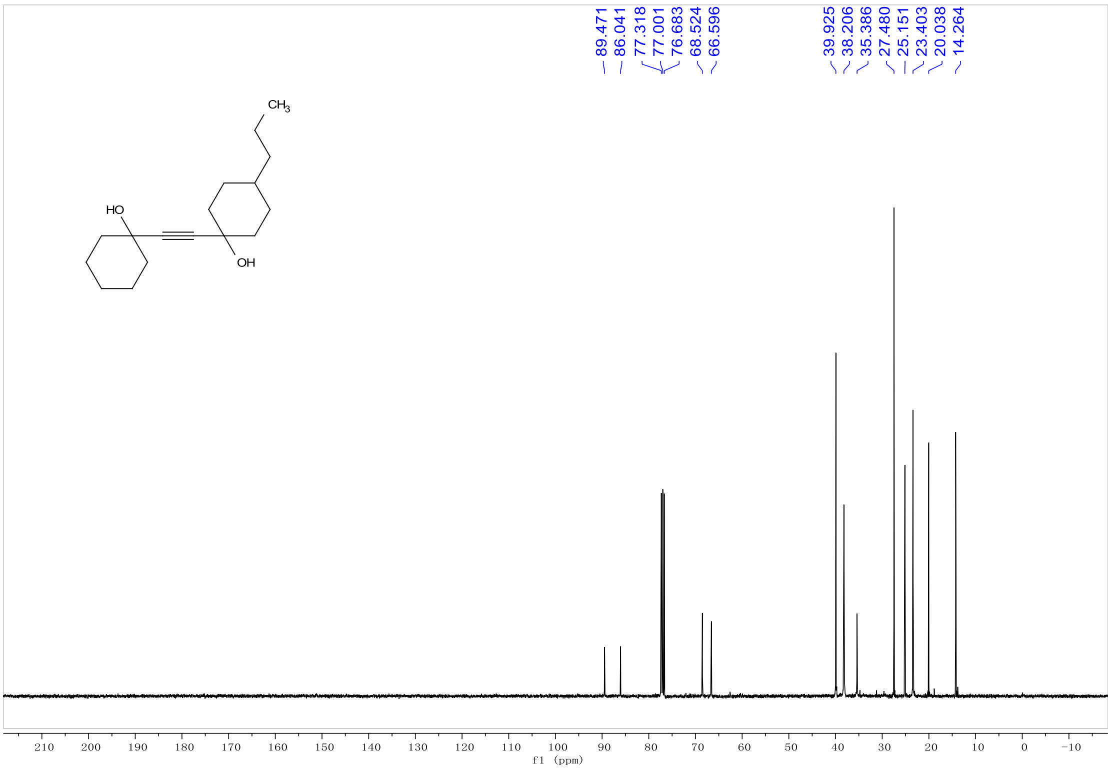

^{1}H NMR (400 MHz, $CDCl_3$) spectrum of compound 1e

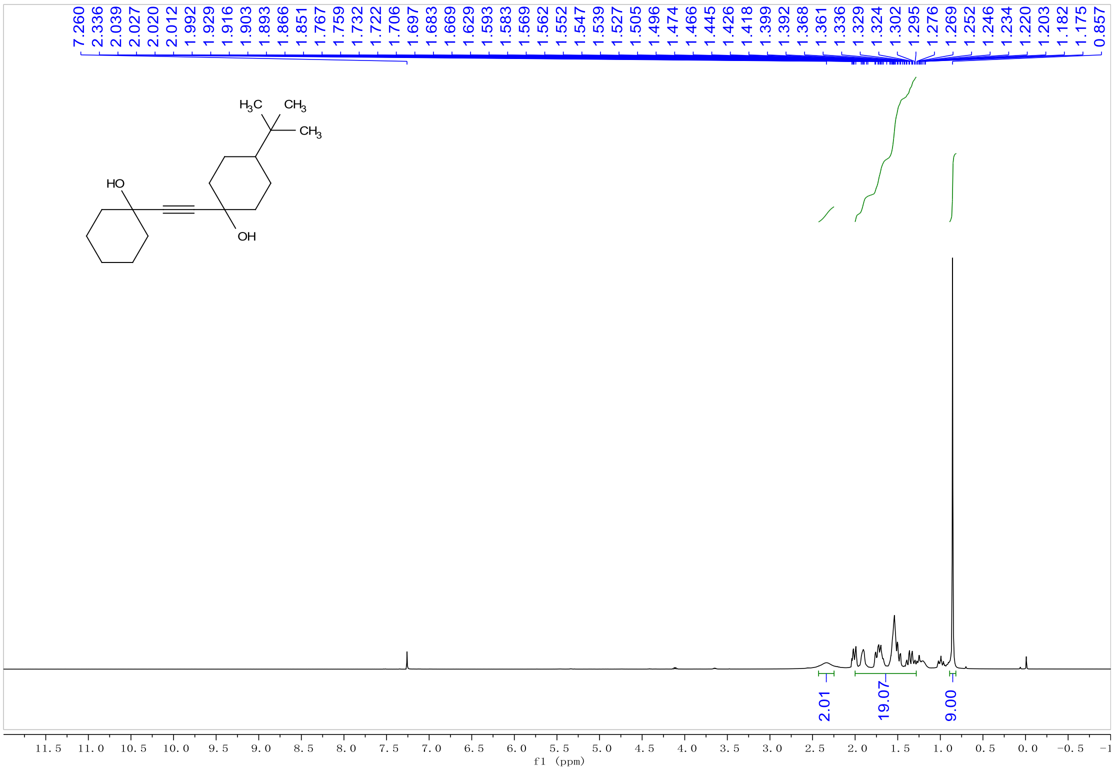

^{13}C NMR (100 MHz, $CDCl_3$) spectrum of compound 1e

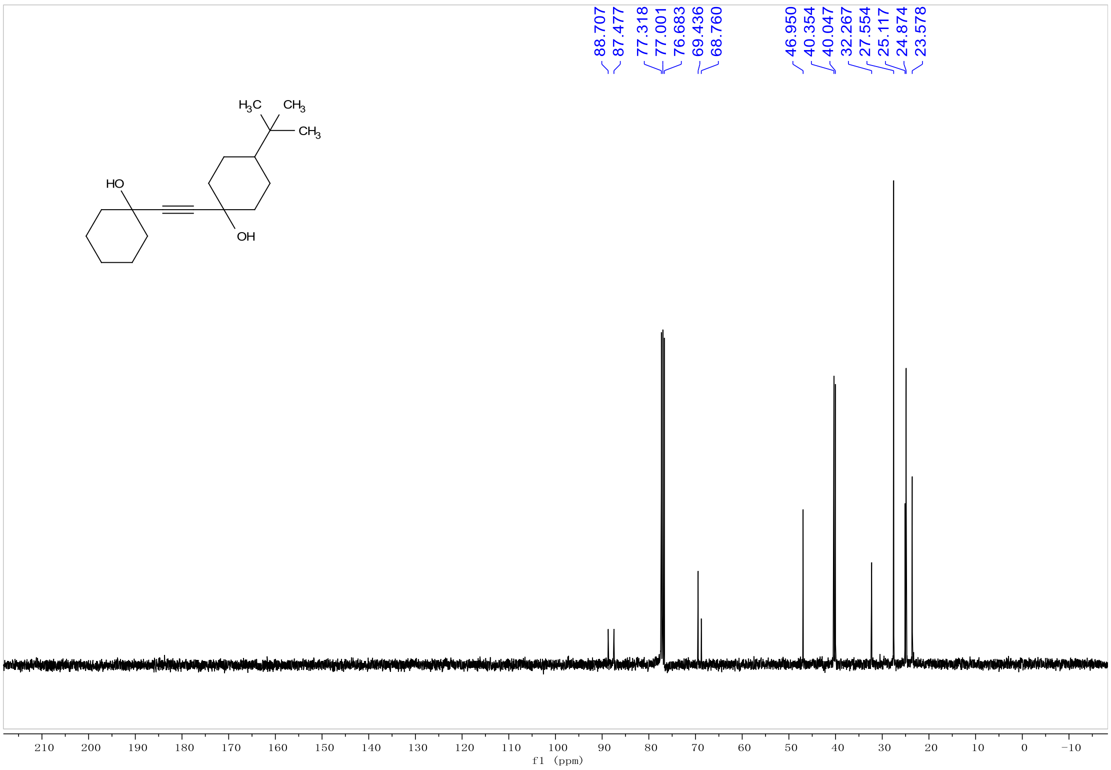

^{1}H NMR (400 MHz, $CDCl_3$) spectrum of compound 1f

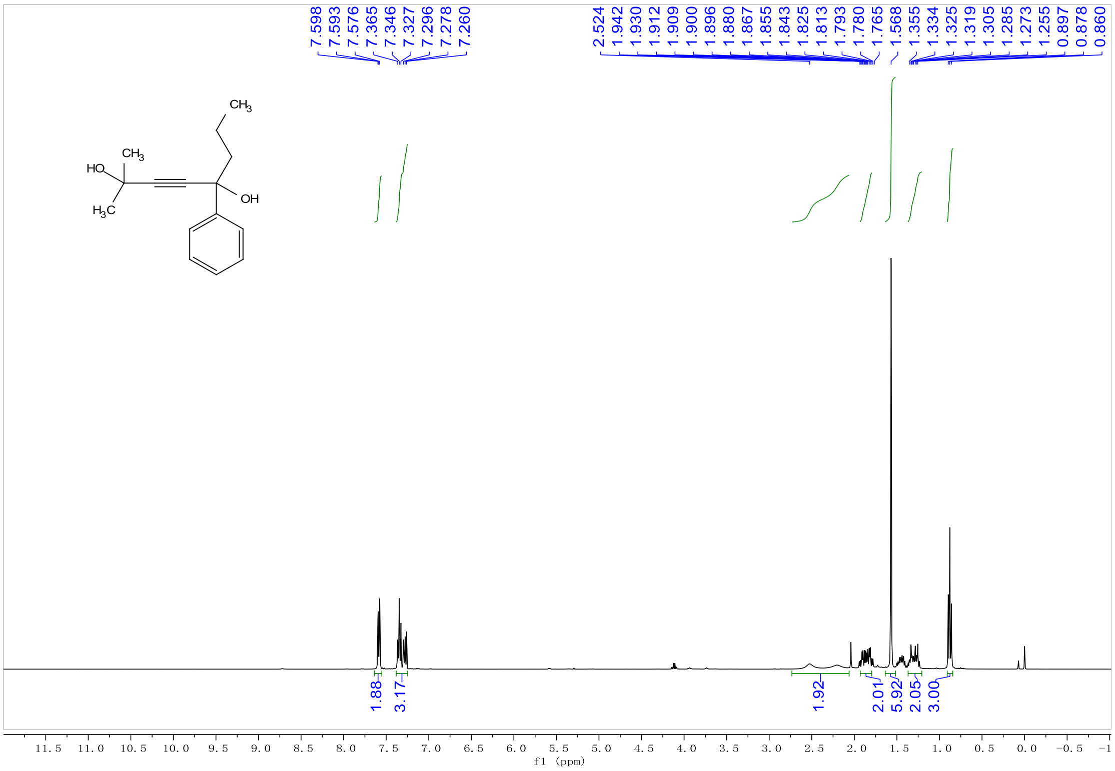

^{13}C NMR (100 MHz, $CDCl_3$) spectrum of compound 1f

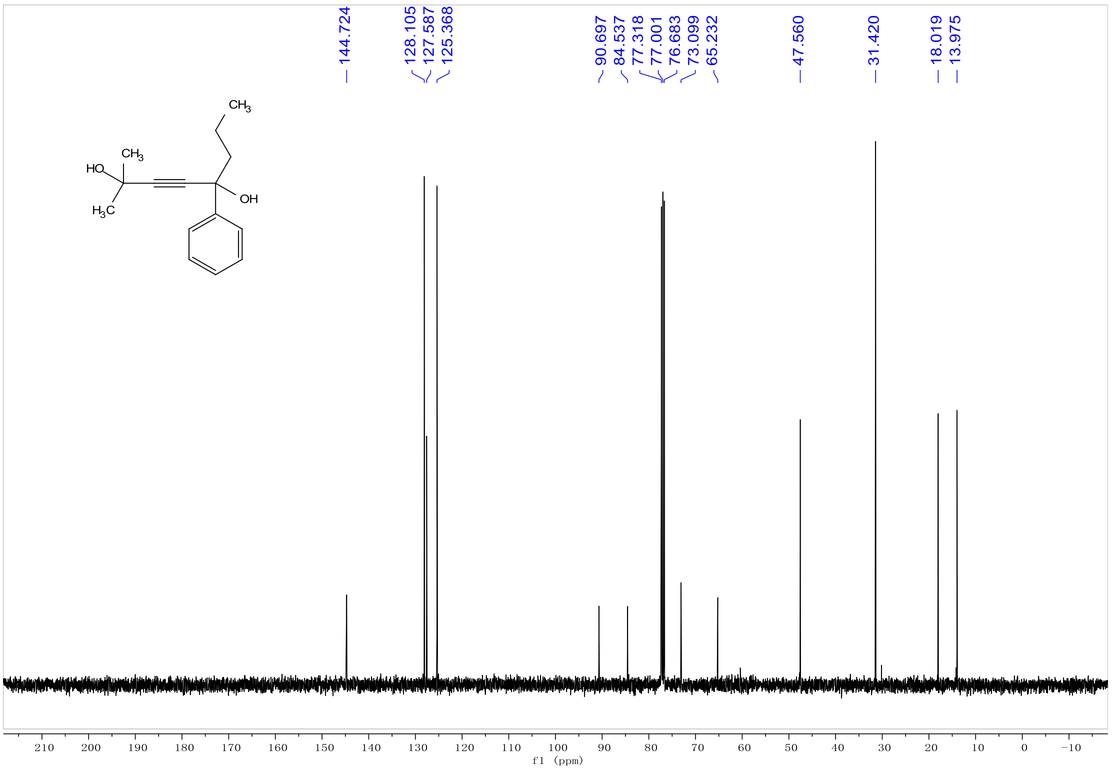

^{1}H NMR (500 MHz, $CDCl_3$) spectrum of compound 2a

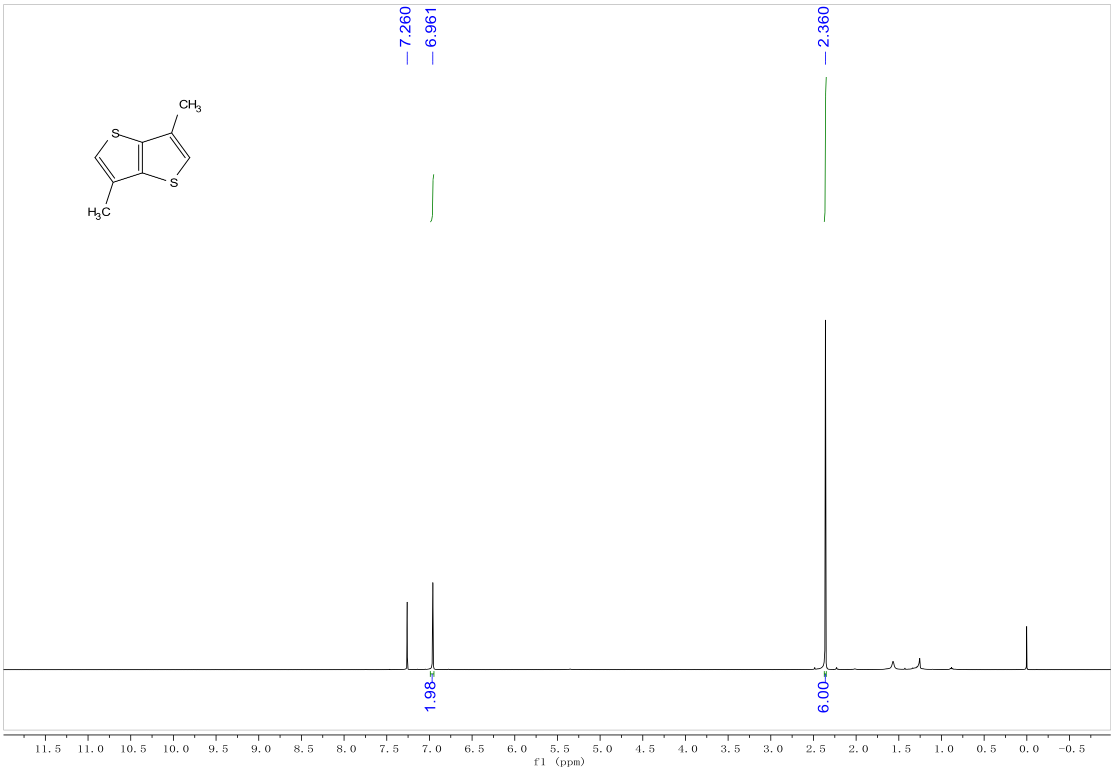

^{13}C NMR (125 MHz, $CDCl_3$) spectrum of compound 2a

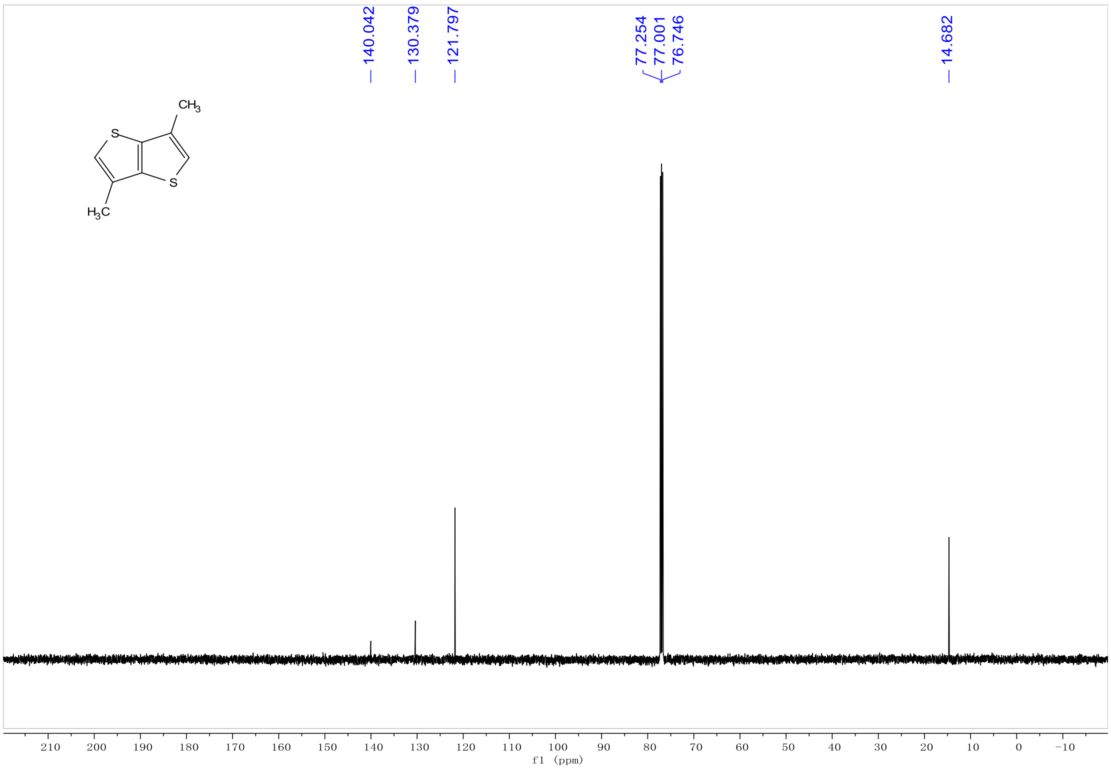

^{1}H NMR (400 MHz, $CDCl_3$) spectrum of compound 2b

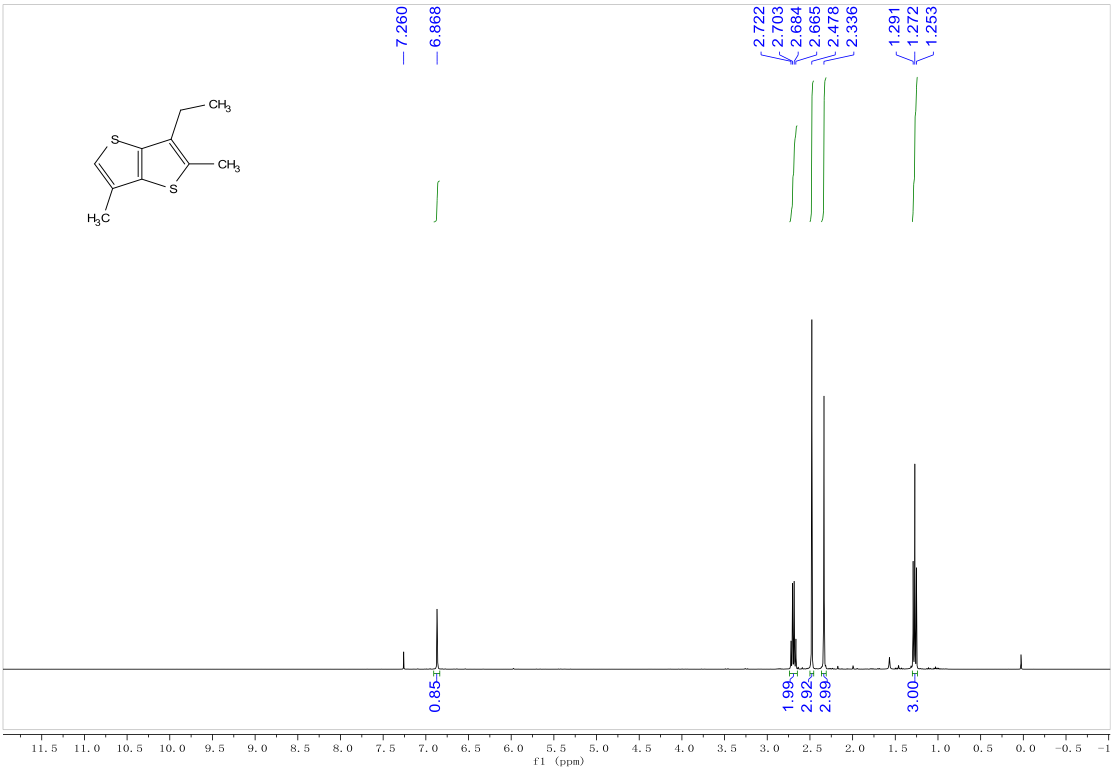

^{13}C NMR (100 MHz, $CDCl_3$) spectrum of compound 2b

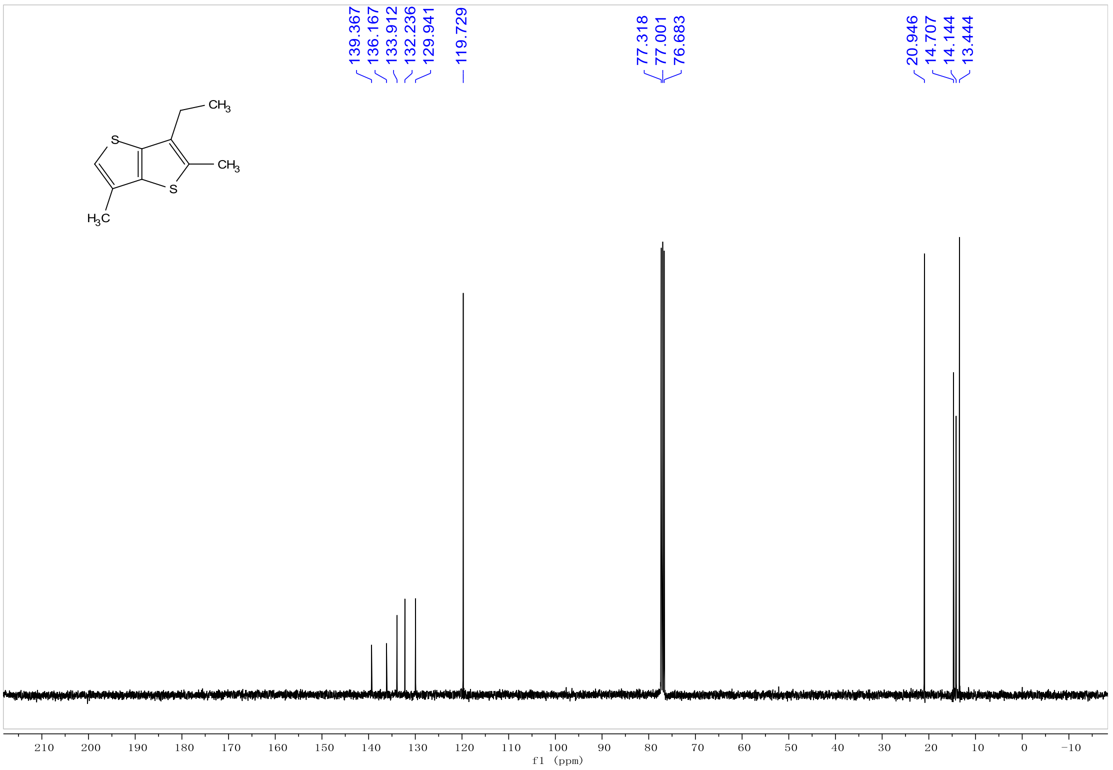

^{1}H NMR (400 MHz, $CDCl_3$) spectrum of compound 2c

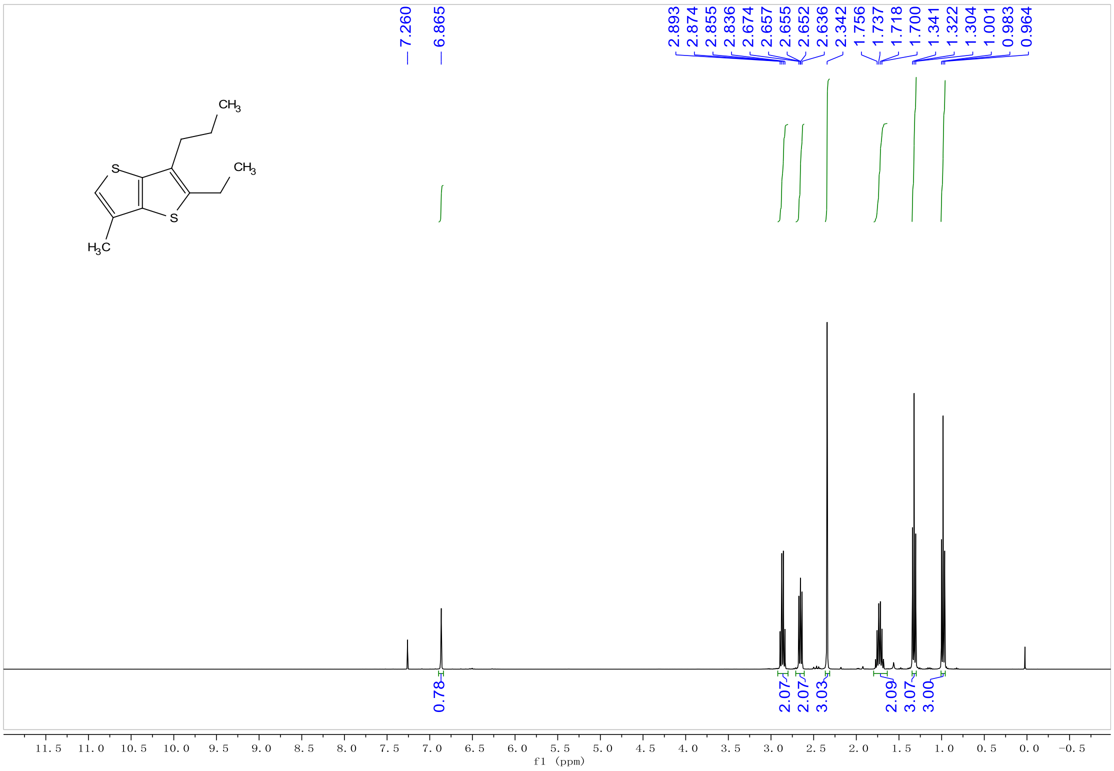

^{13}C NMR (100 MHz, $CDCl_3$) spectrum of compound 2c

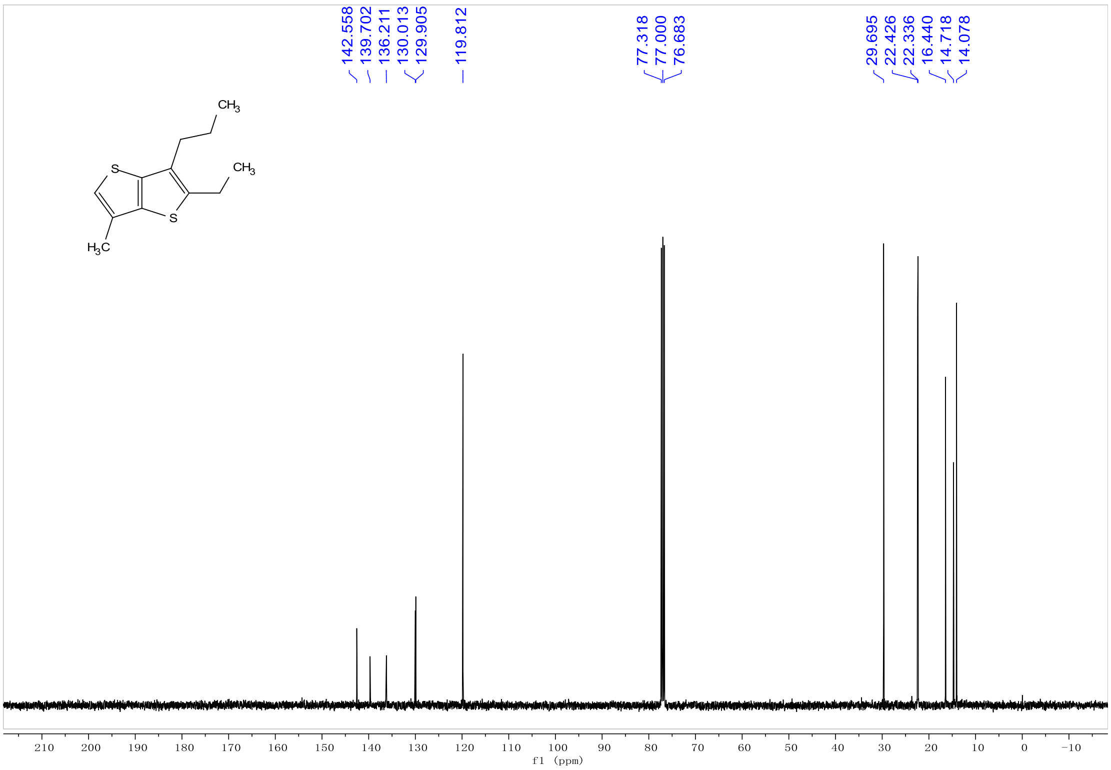

^1H NMR (500 MHz, $CDCl_3$) spectrum of compound 2d

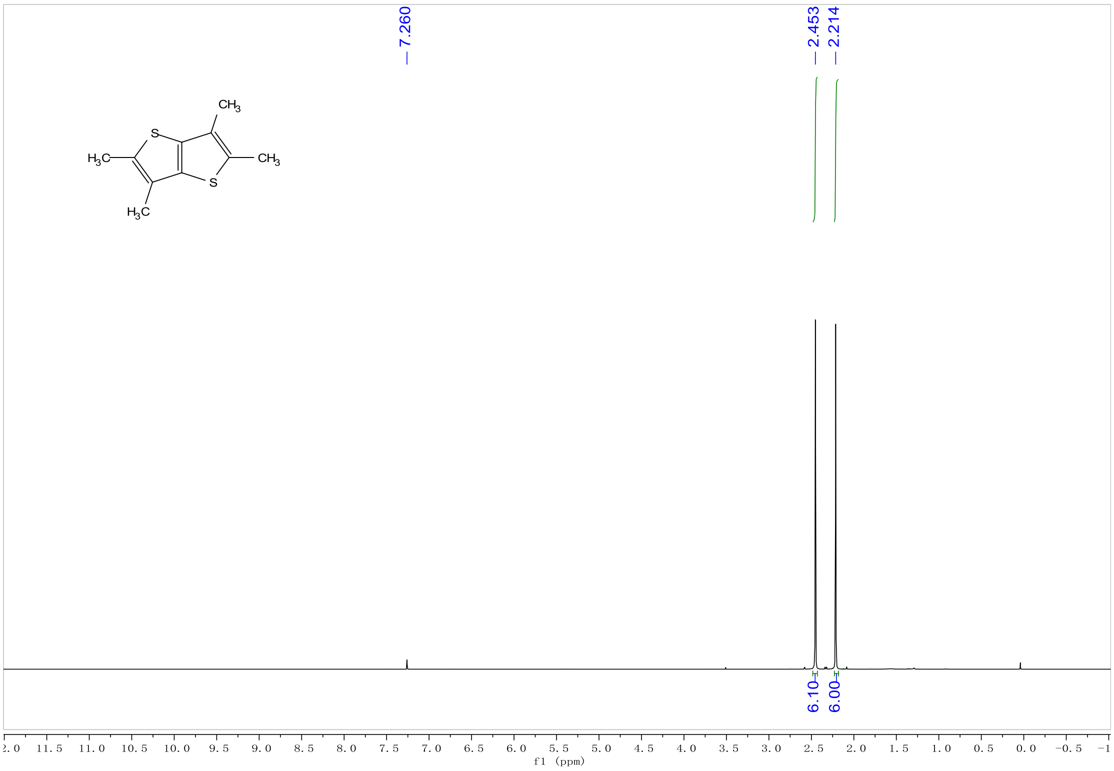

^{13}C NMR (125 MHz, $CDCl_3$) spectrum of compound 2d

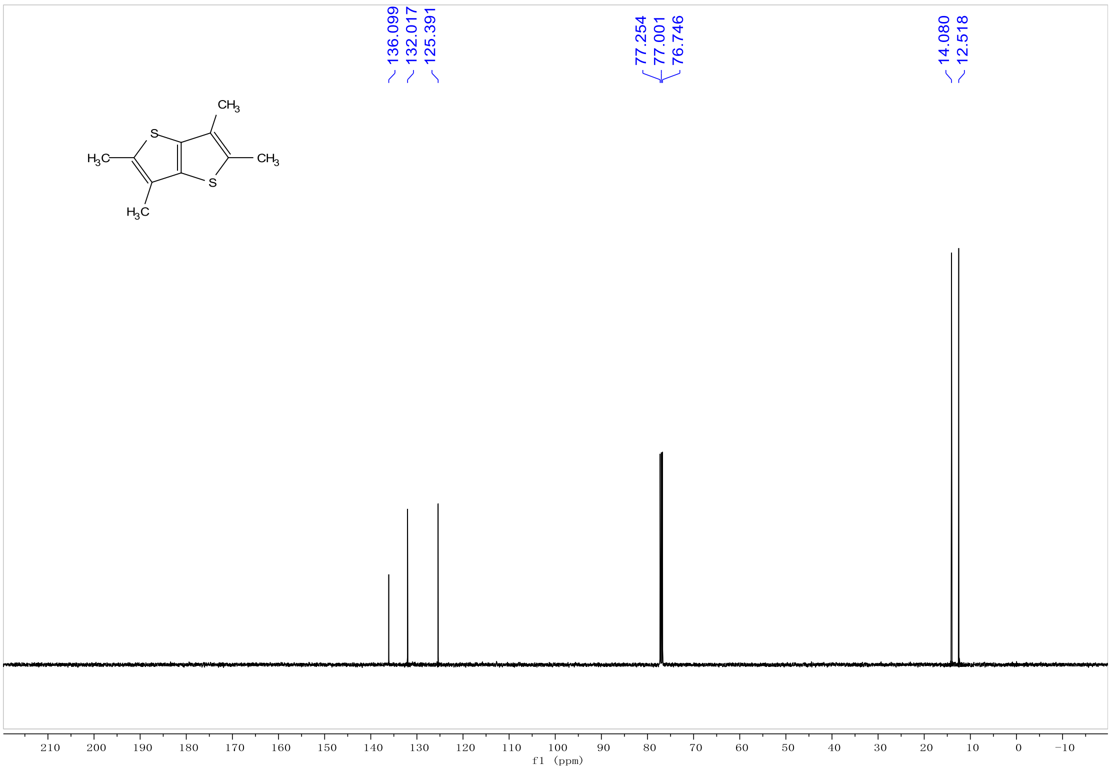

^{1}H NMR (400 MHz, $CDCl_3$) spectrum of compound 2e

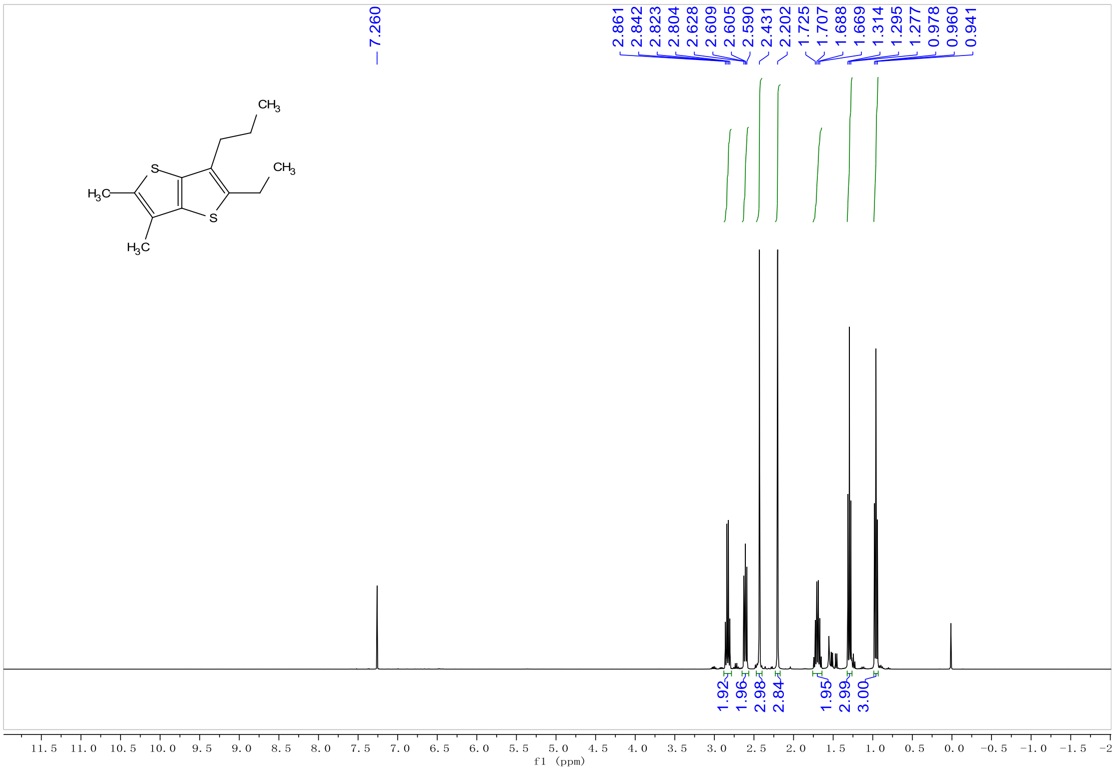

^{13}C NMR (100 MHz, $CDCl_3$) spectrum of compound 2e

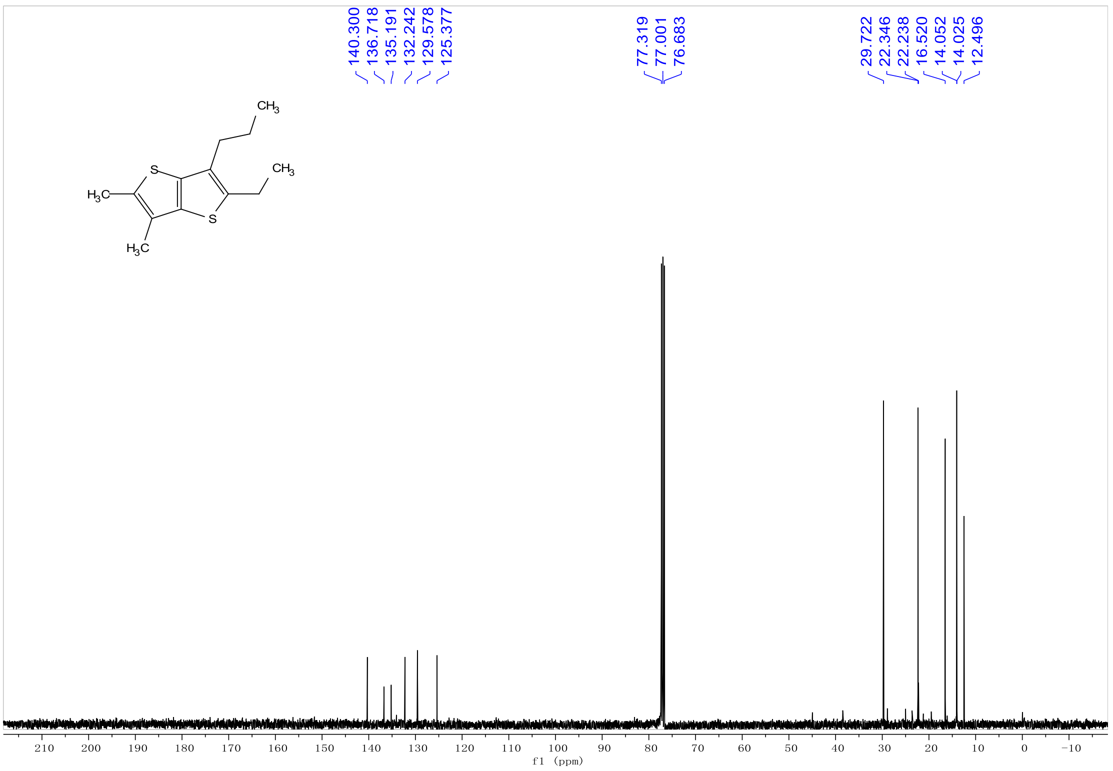

^{1}H NMR (500 MHz, $CDCl_3$) spectrum of compound 2f

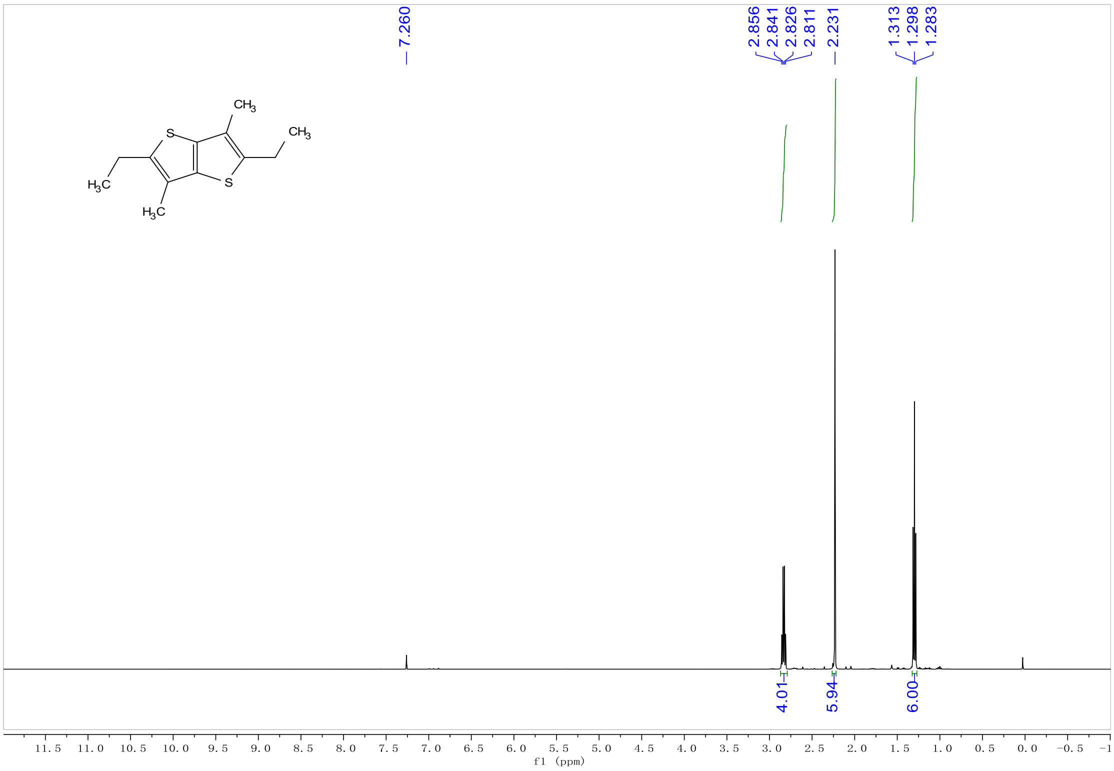

^{13}C NMR (125 MHz, $CDCl_3$) spectrum of compound 2f

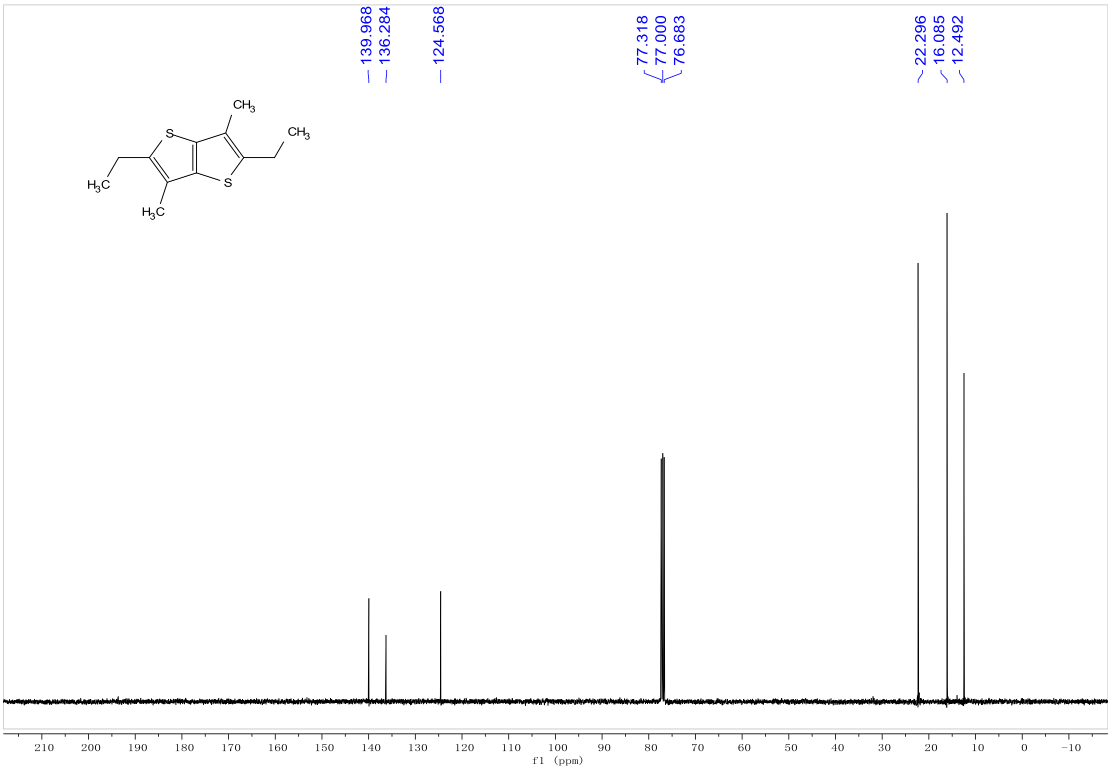

^{1}H NMR (400 MHz, $CDCl_3$) spectrum of compound 2g

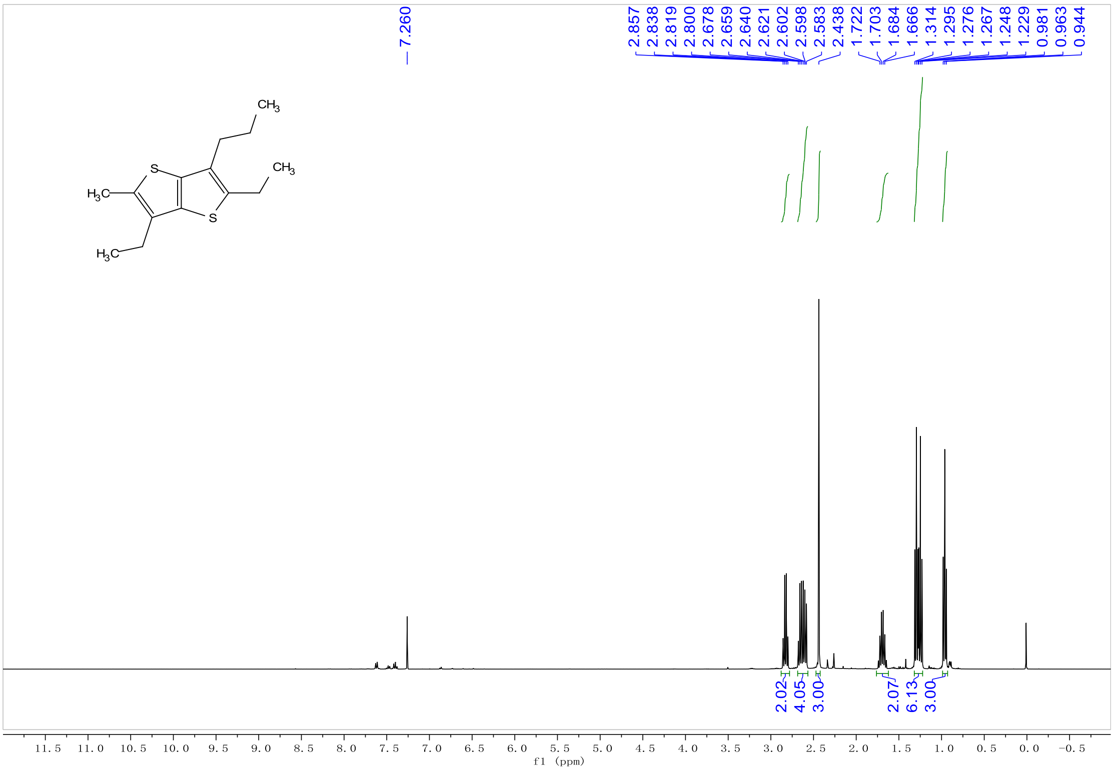

^{13}C NMR (100 MHz, $CDCl_3$) spectrum of compound 2g

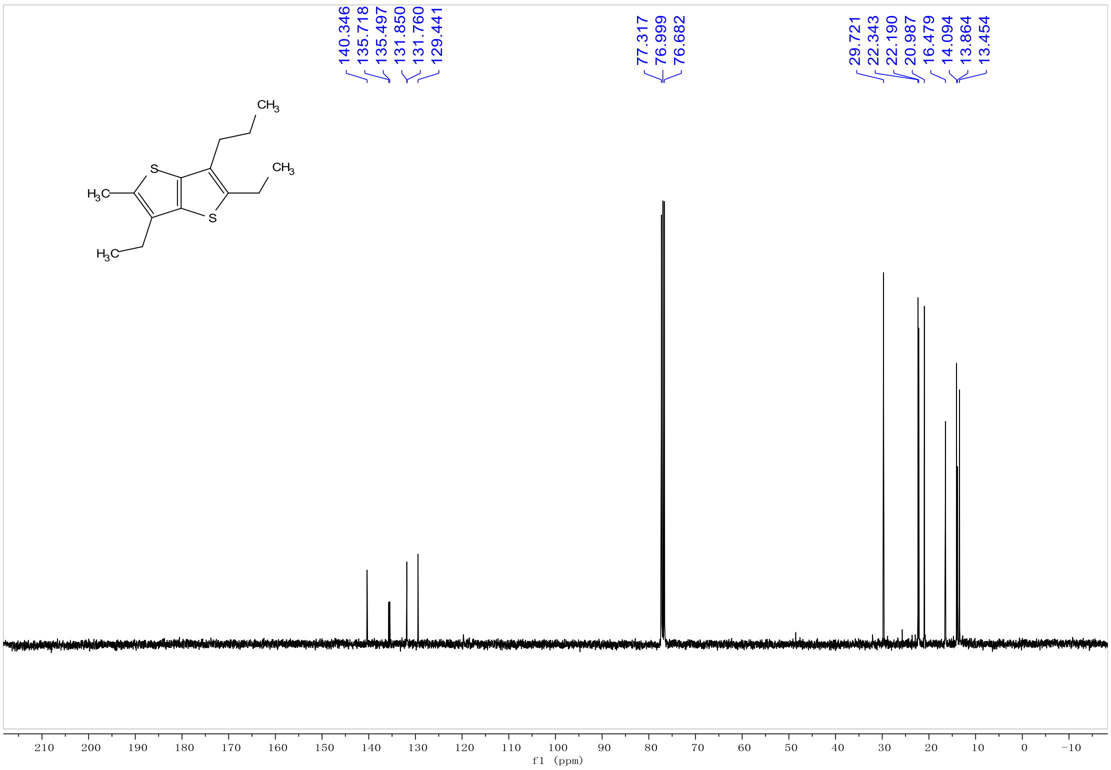

^{1}H NMR (500 MHz, $CDCl_3$) spectrum of compound 2h

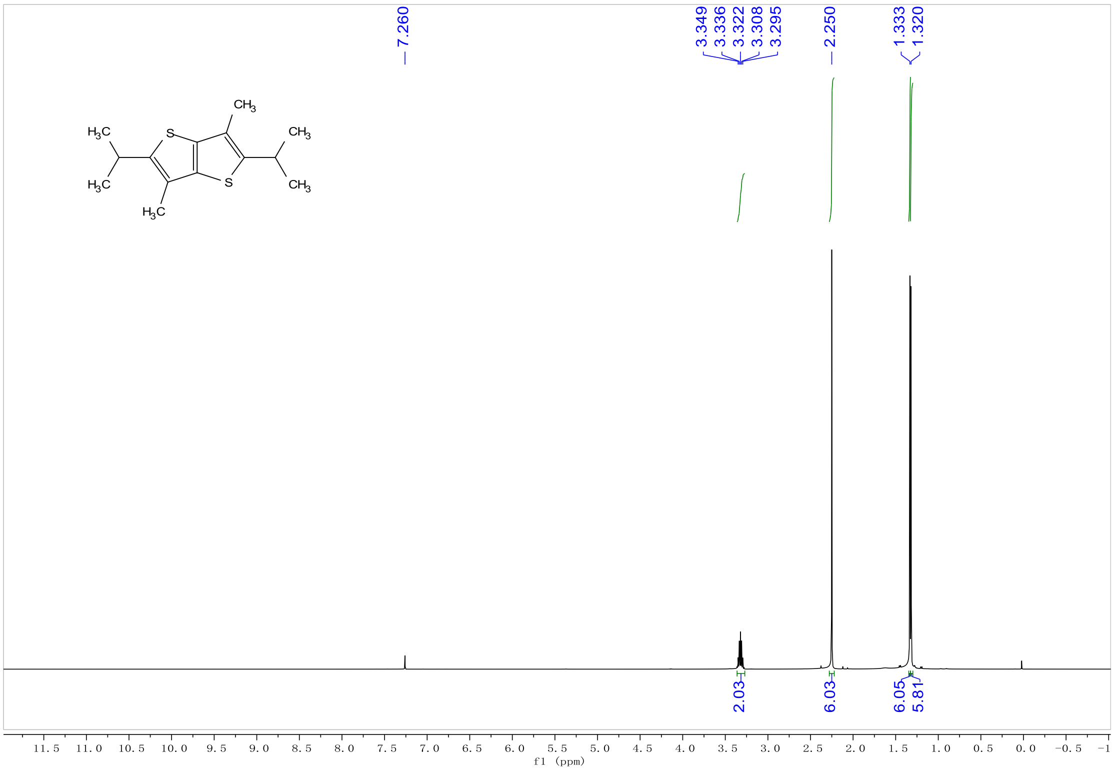

^{13}C NMR (125 MHz, $CDCl_3$) spectrum of compound 2h

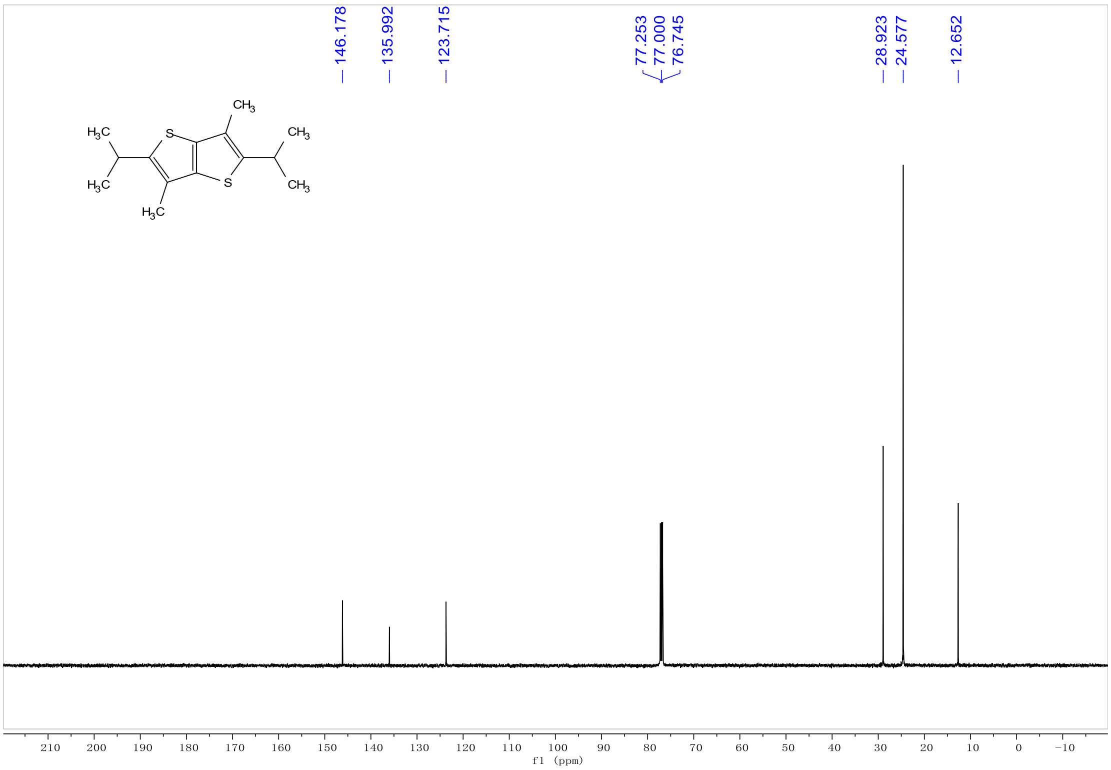

^{1}H NMR (500 MHz, $CDCl_3$) spectrum of compound 2i

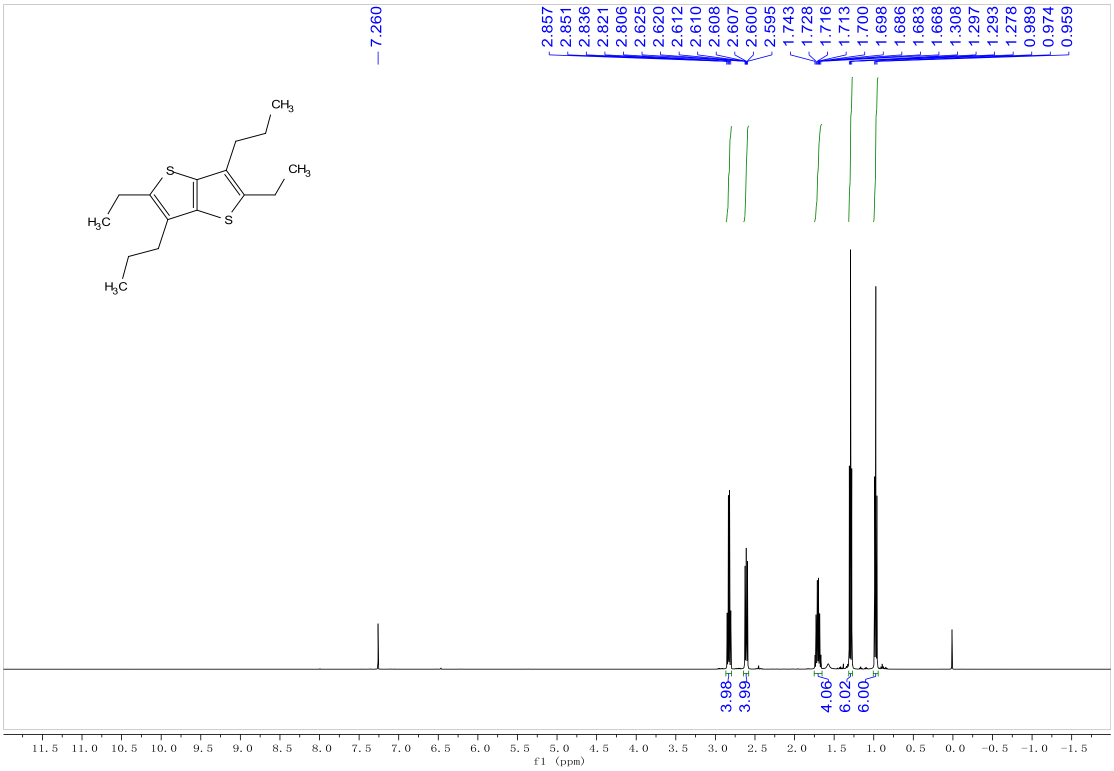

^{13}C NMR (125 MHz, $CDCl_3$) spectrum of compound 2i

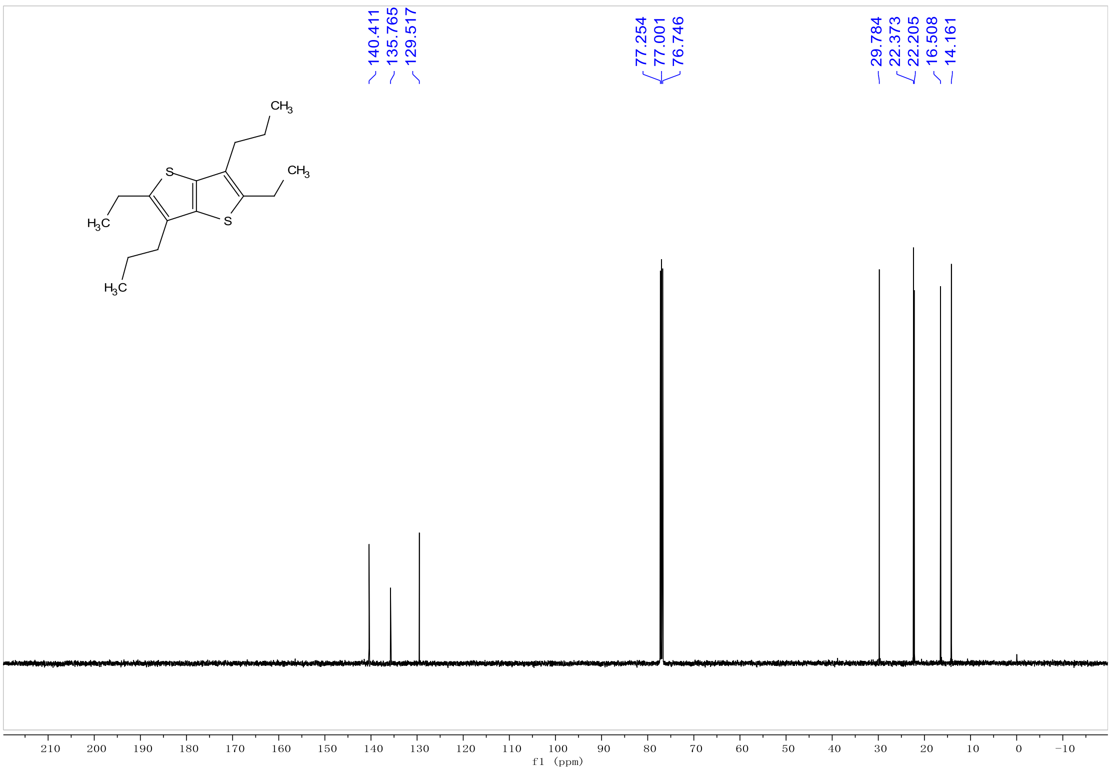

^{1}H NMR (500 MHz, $CDCl_3$) spectrum of compound 2j

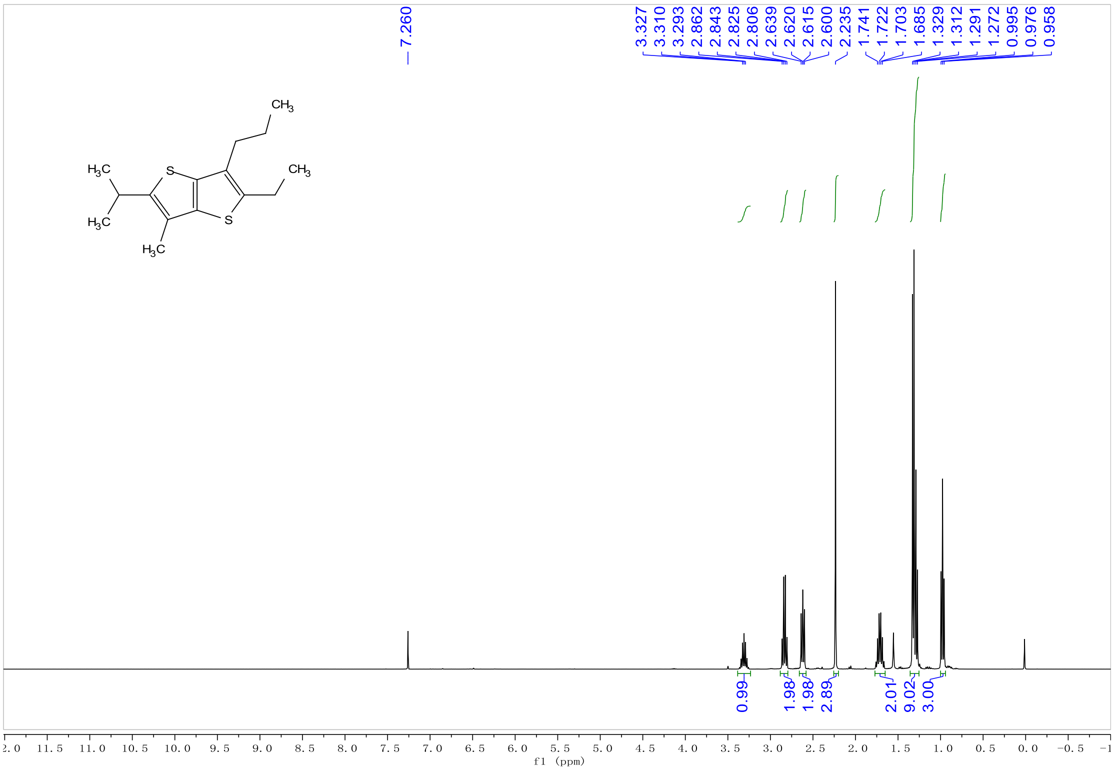

^{13}C NMR (125 MHz, $CDCl_3$) spectrum of compound 2j

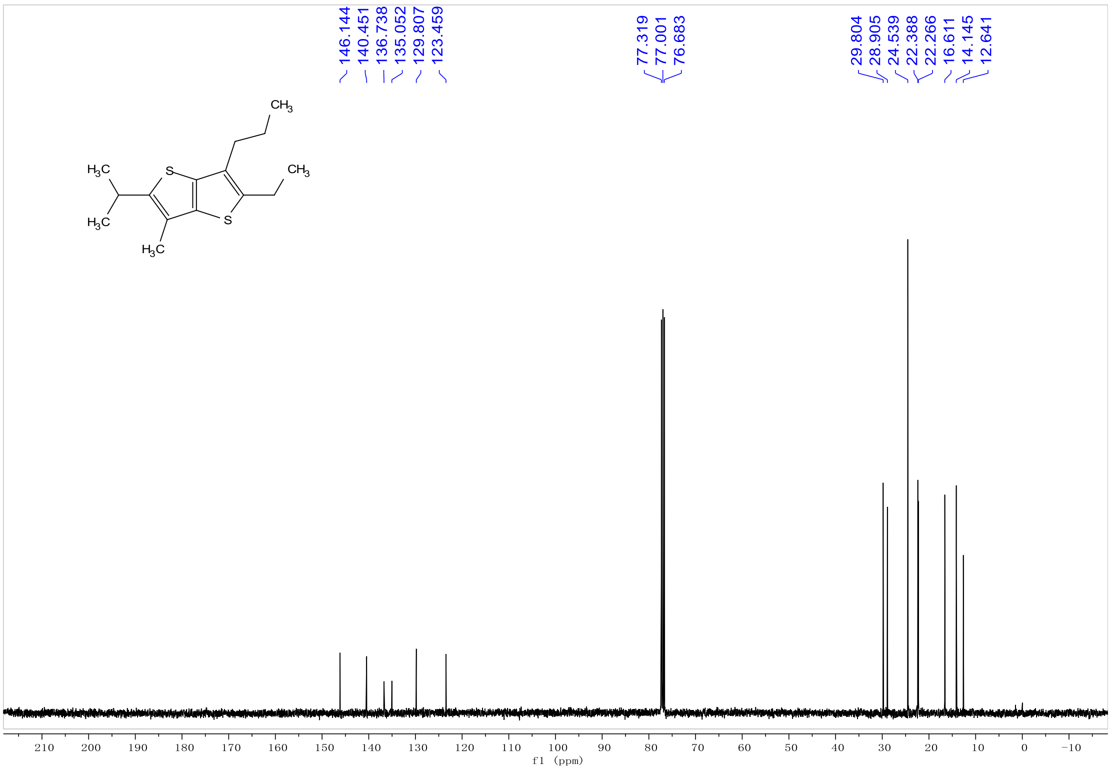

^{1}H NMR (400 MHz, $CDCl_3$) spectrum of compound 2k

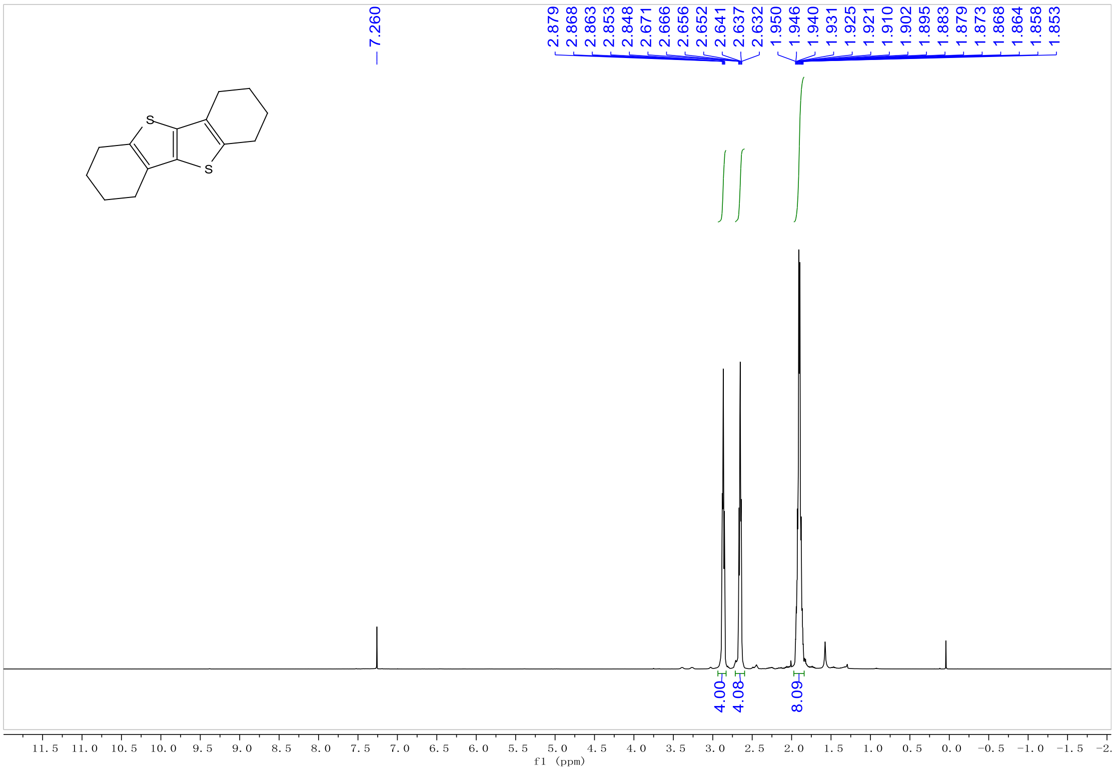

^{13}C NMR (100 MHz, $CDCl_3$) spectrum of compound 2k

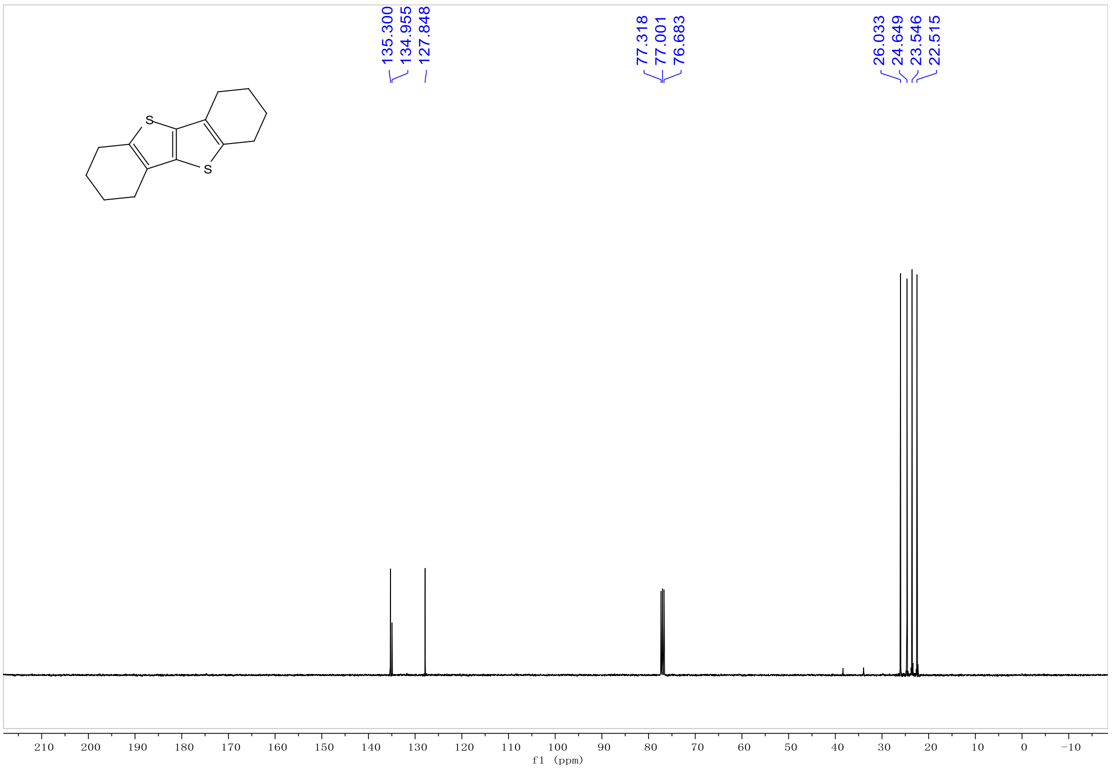

^{1}H NMR (400 MHz, $CDCl_3$) spectrum of compound 2l

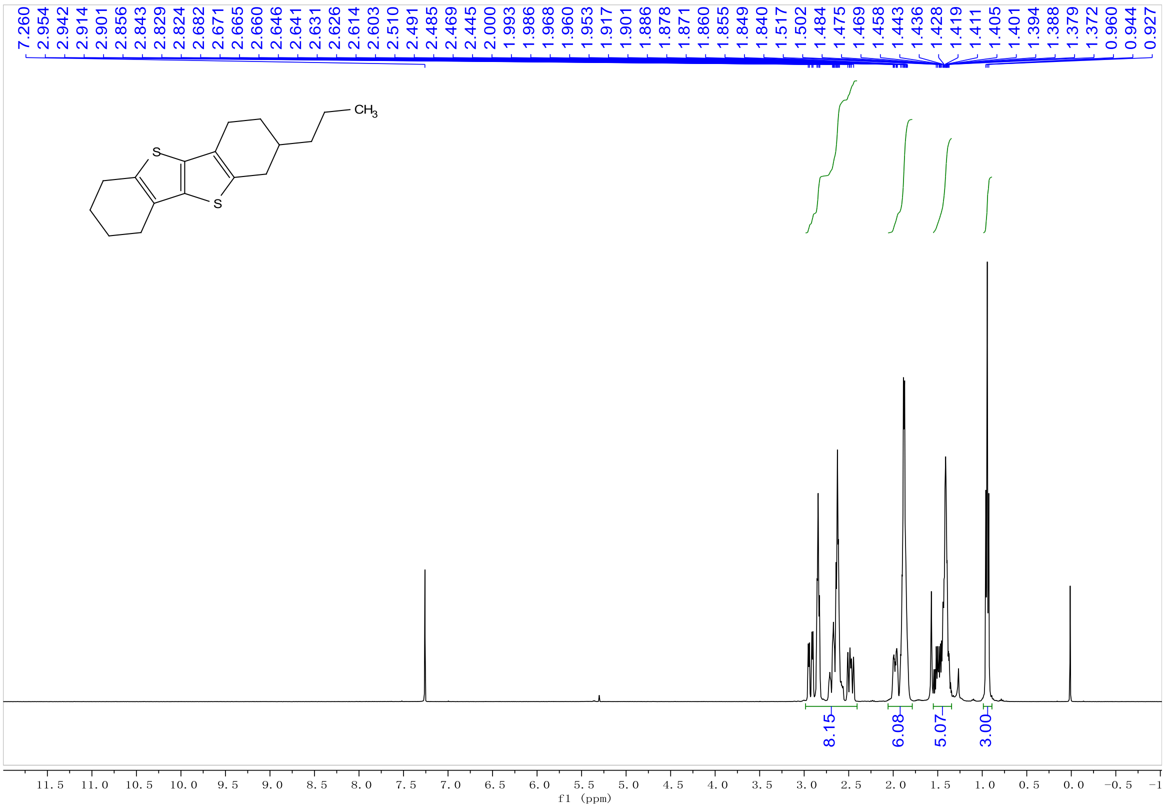

^{13}C NMR (100 MHz, $CDCl_3$) spectrum of compound 2l

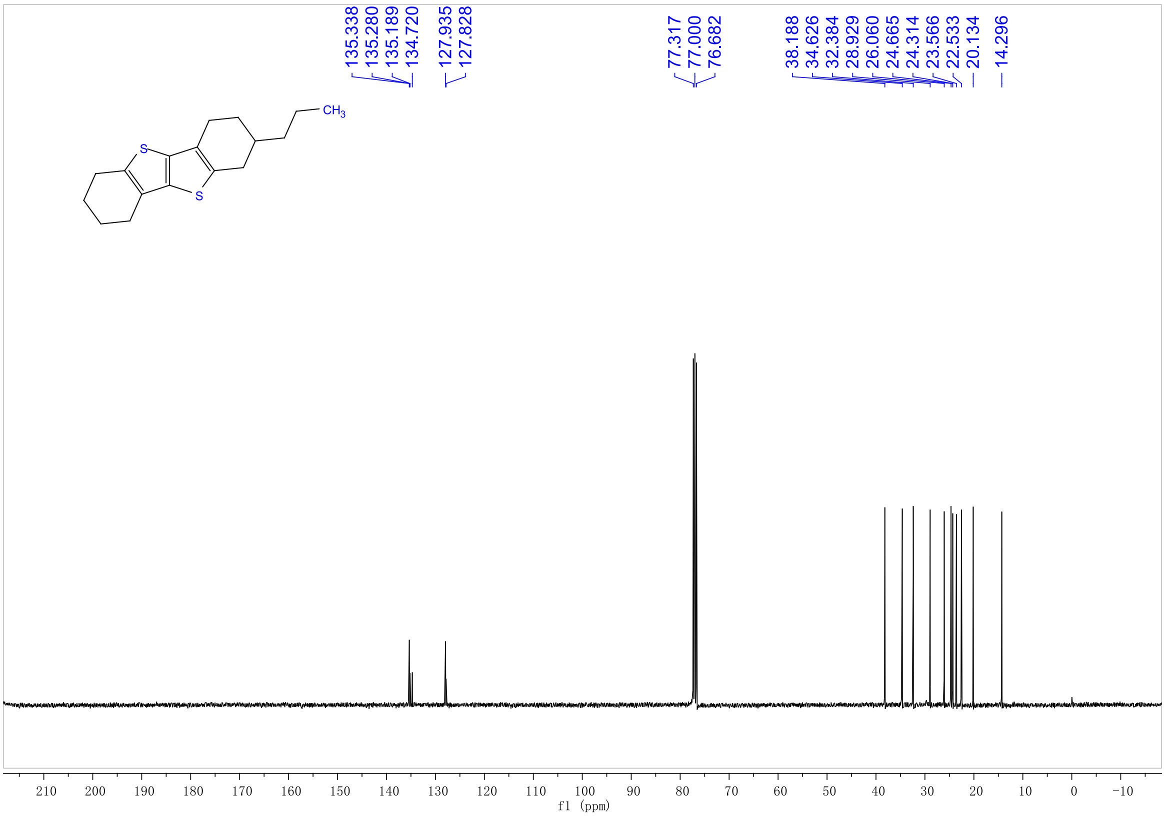

^{1}H NMR (400 MHz, $CDCl_3$) spectrum of compound 2m

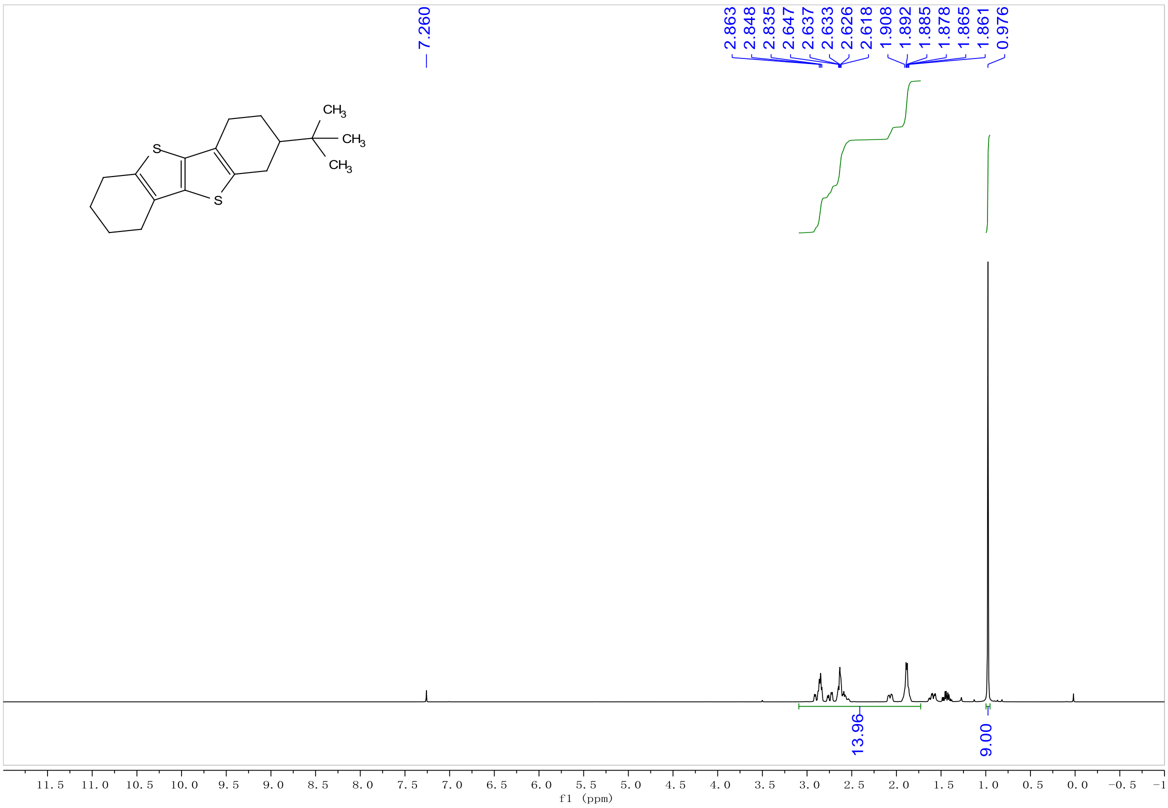

^{13}C NMR (100 MHz, $CDCl_3$) spectrum of compound 2m

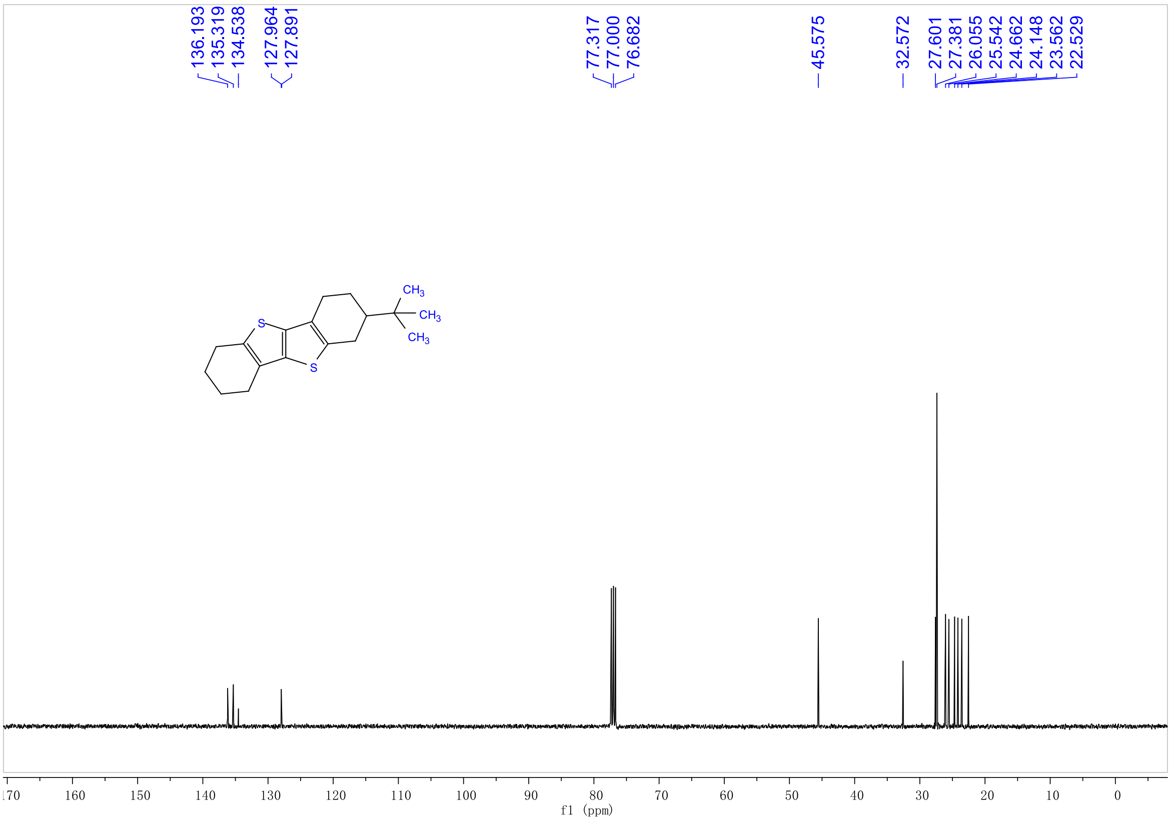

^{1}H NMR (400 MHz, $CDCl_3$) spectrum of compound 2n

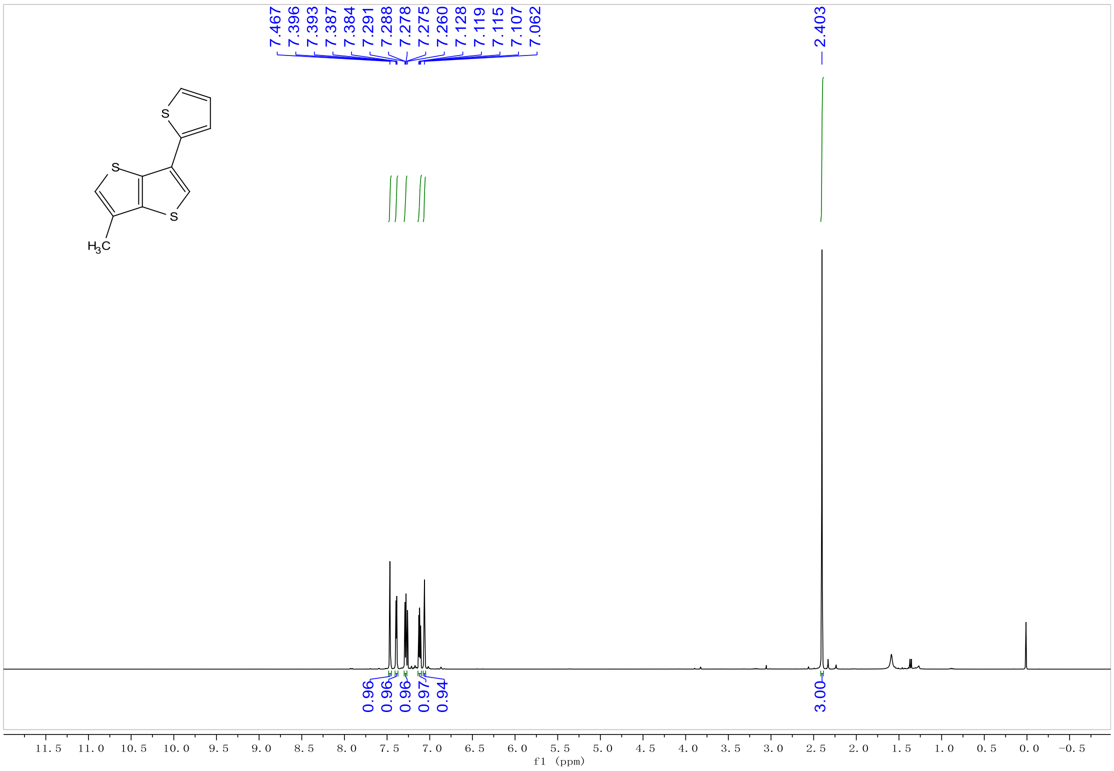

^{13}C NMR (100 MHz, $CDCl_3$) spectrum of compound 2n

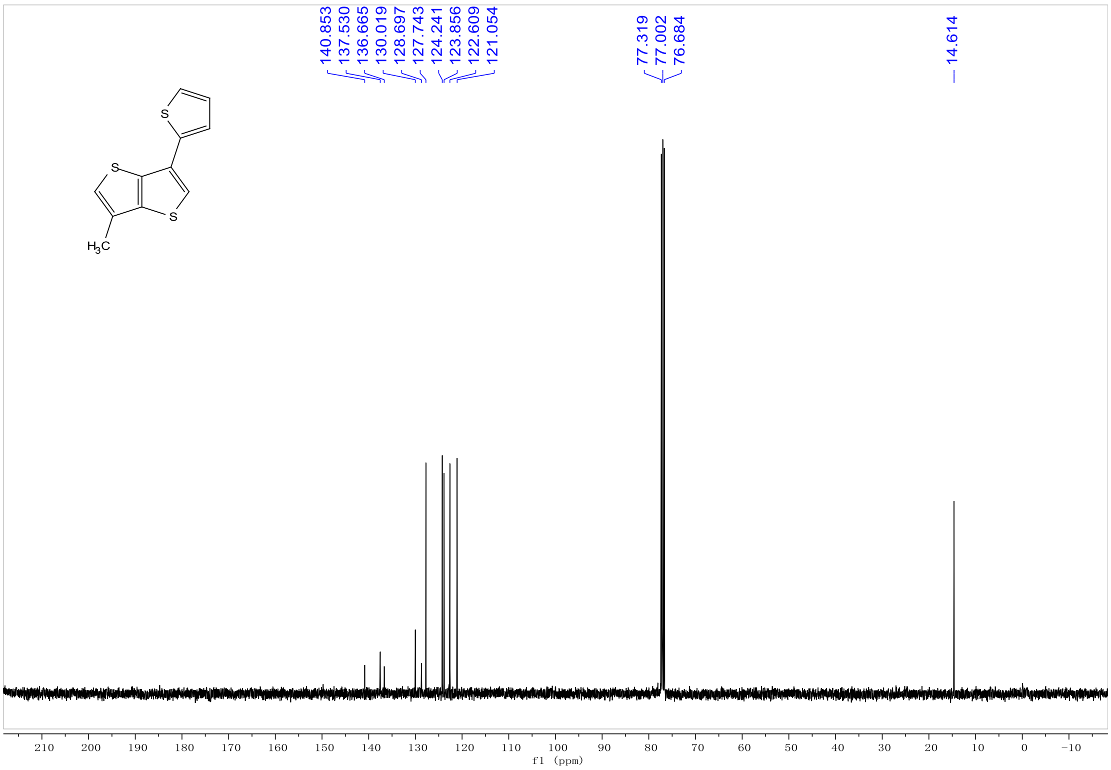

^{1}H NMR (400 MHz, $CDCl_3$) spectrum of compound 2o

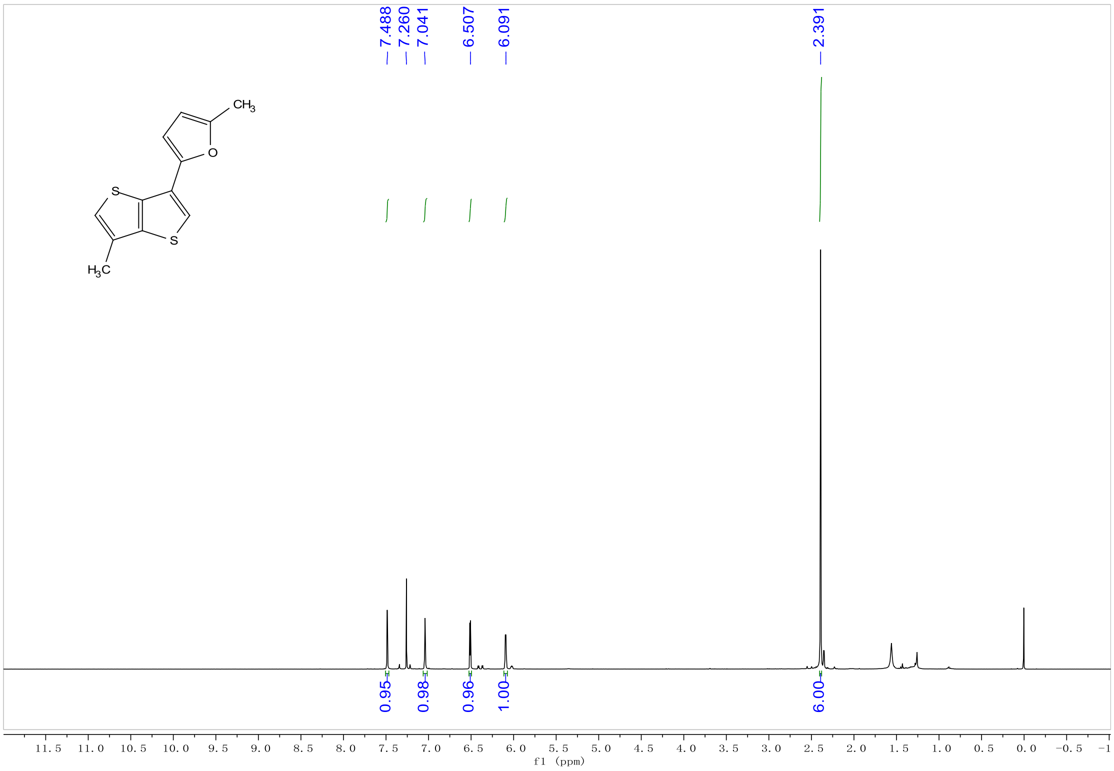

^{13}C NMR (100 MHz, $CDCl_3$) spectrum of compound 2o

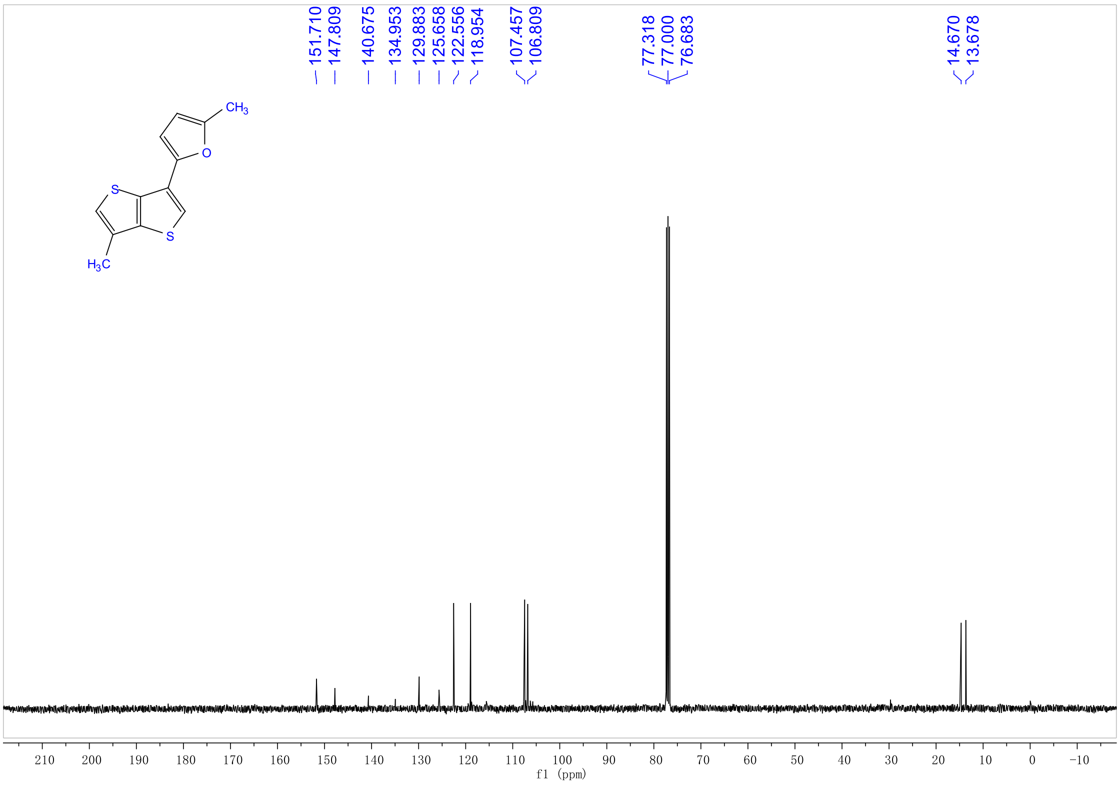

^{1}H NMR (400 MHz, $CDCl_3$) spectrum of compound 2p

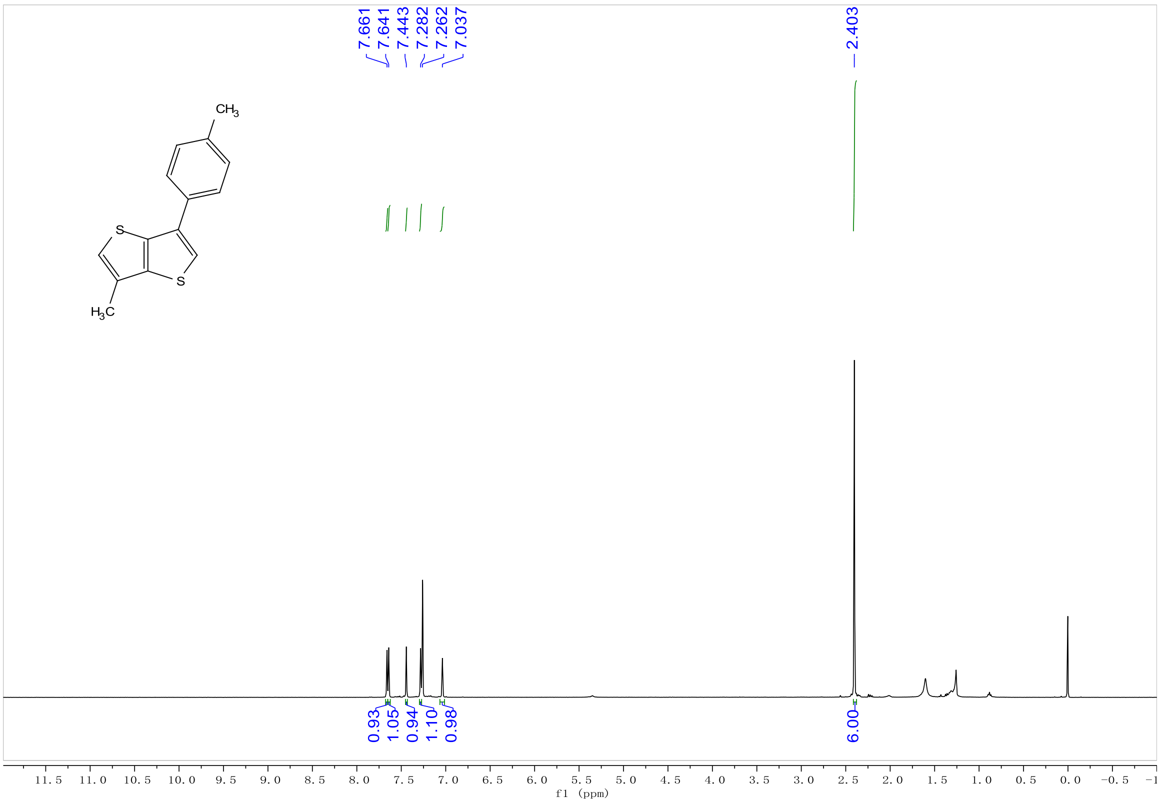

^{13}C NMR (100 MHz, $CDCl_3$) spectrum of compound 2p

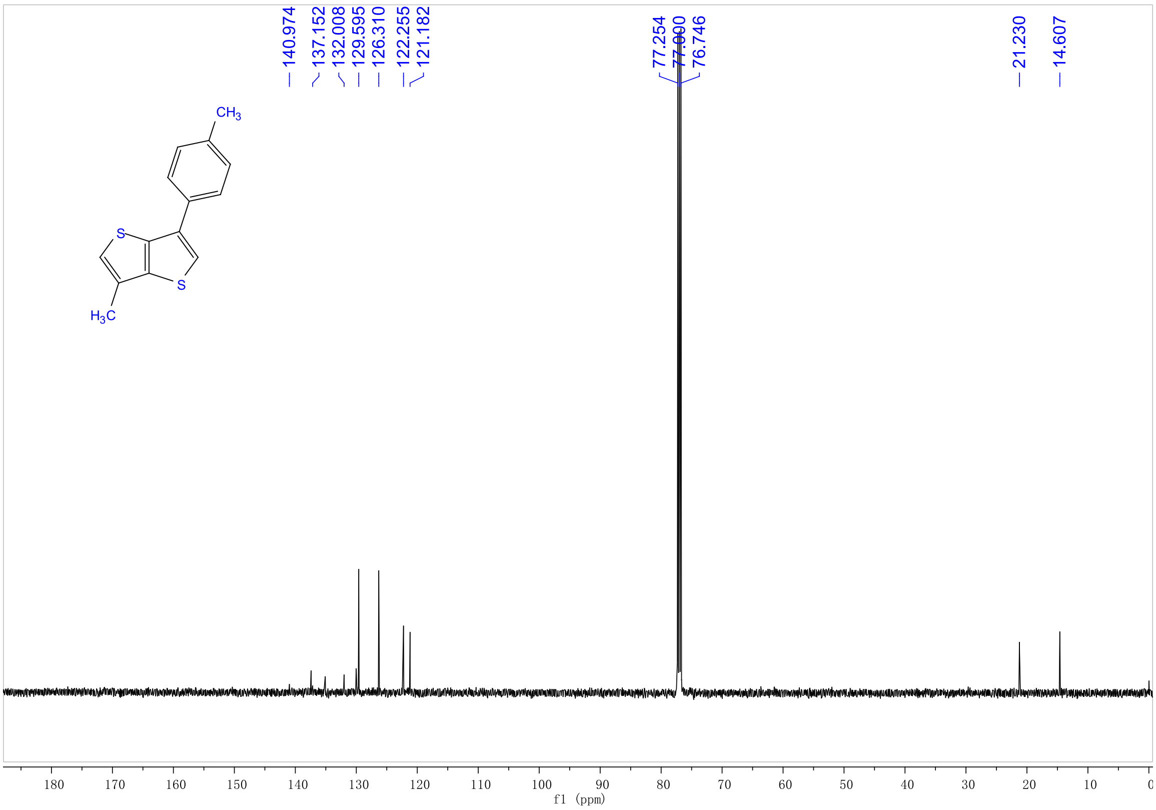

^{1}H NMR (400 MHz, $CDCl_3$) spectrum of compound 2q

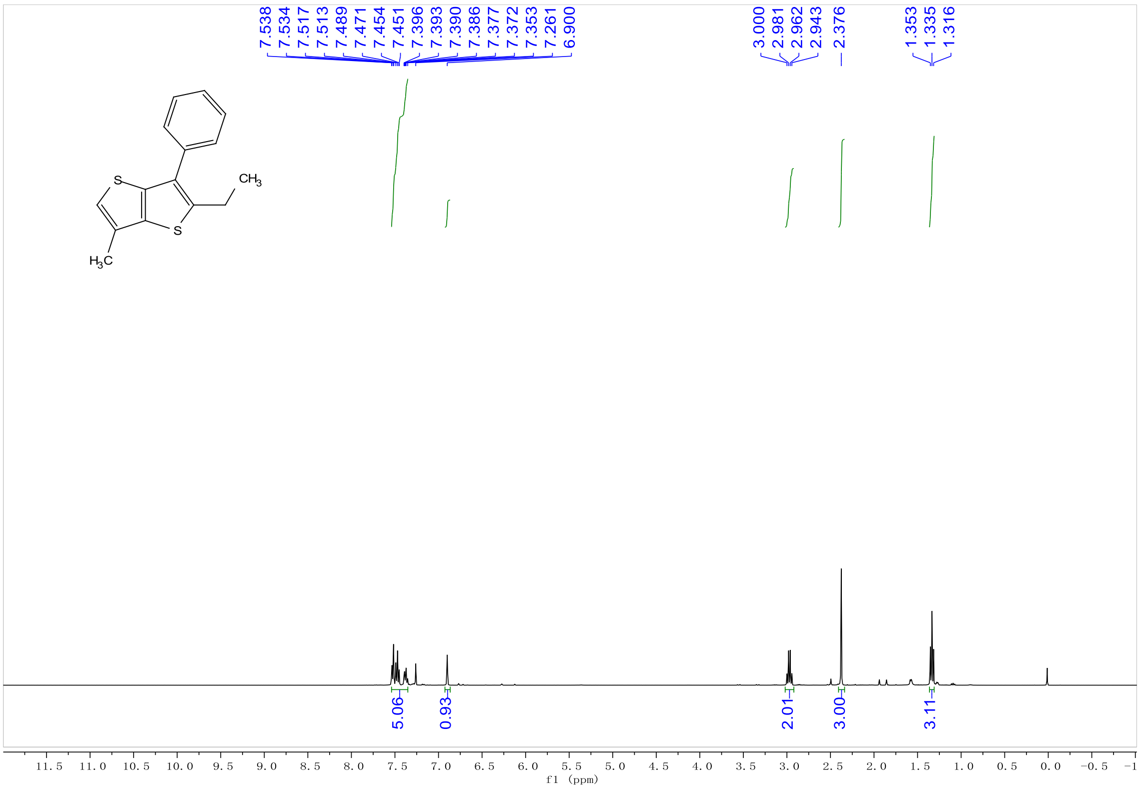

^{13}C NMR (100 MHz, $CDCl_3$) spectrum of compound 2q

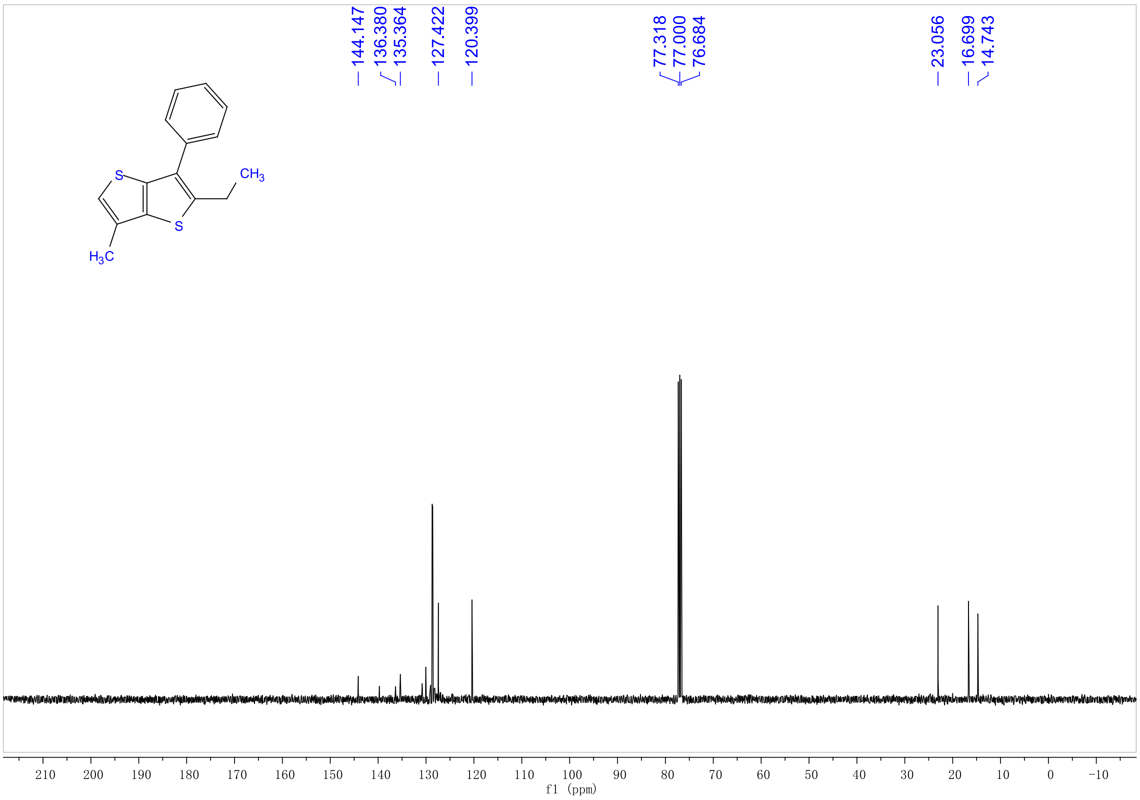

^{1}H NMR (400 MHz, $CDCl_3$) spectrum of compound 2r

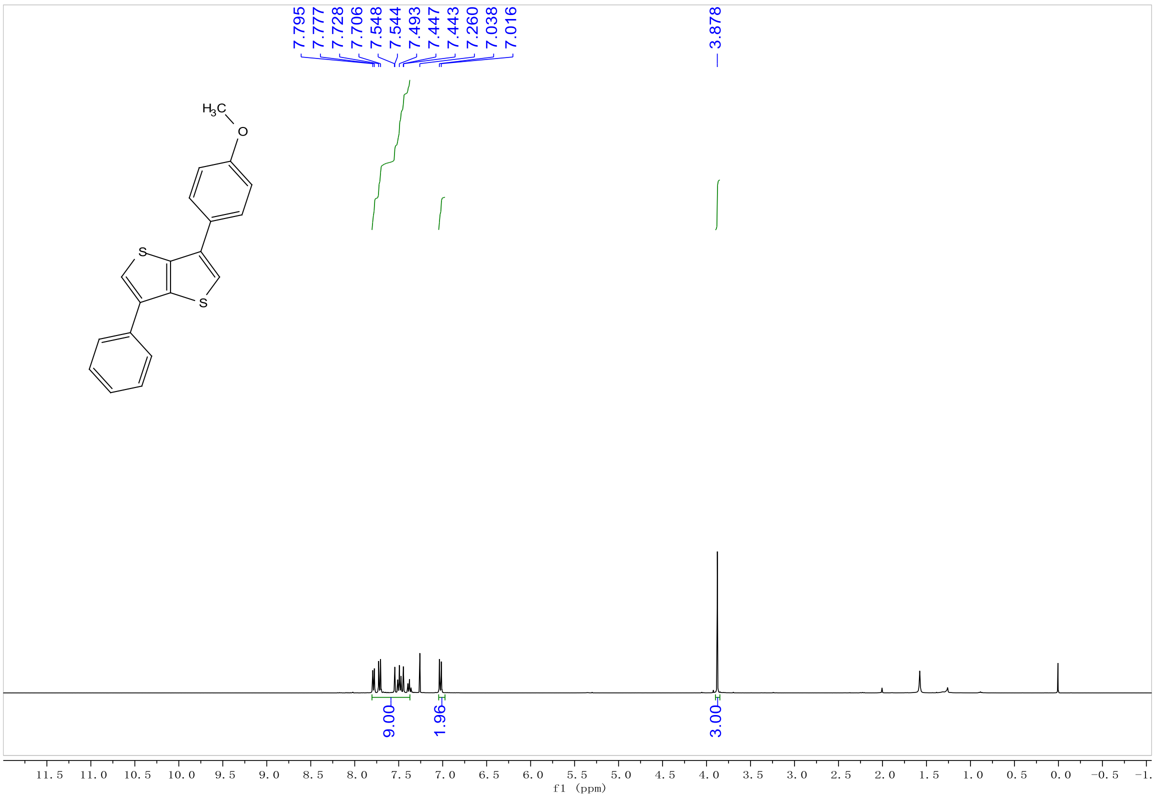

^{13}C NMR (125 MHz, $CDCl_3$) spectrum of compound 2r

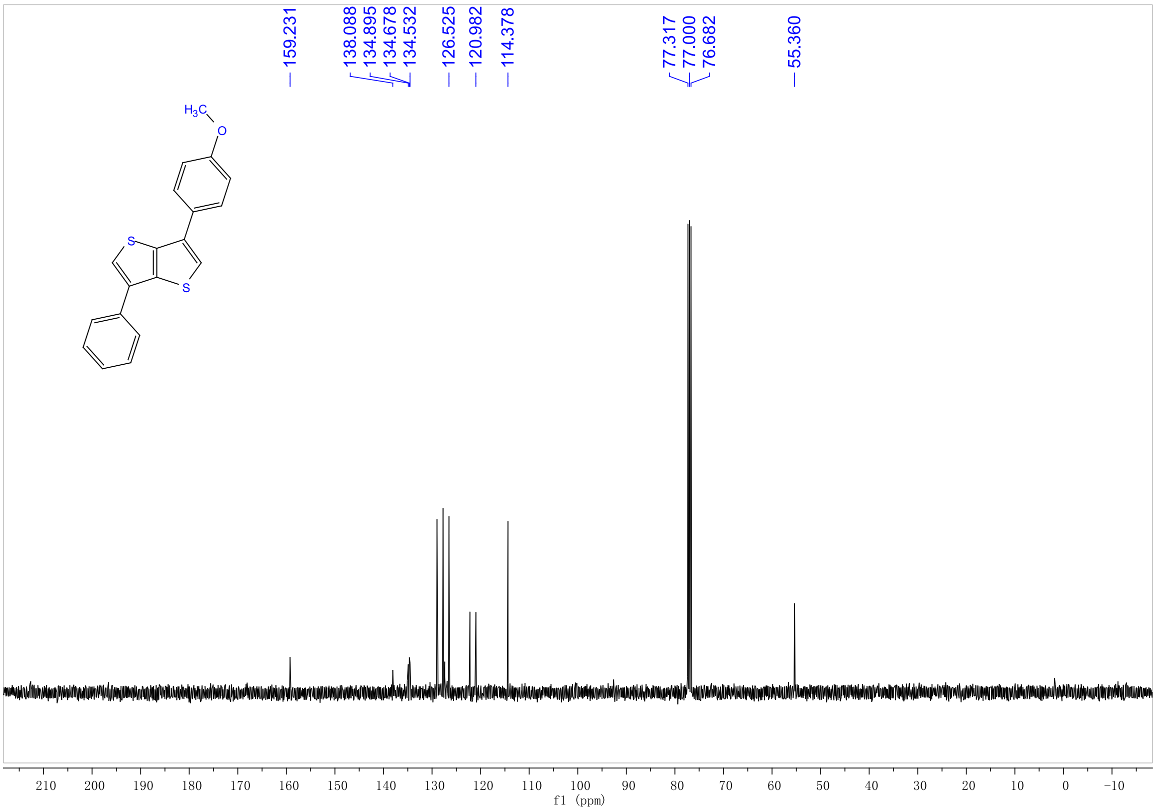

^{1}H NMR (400 MHz, $CDCl_3$) spectrum of compound 2s

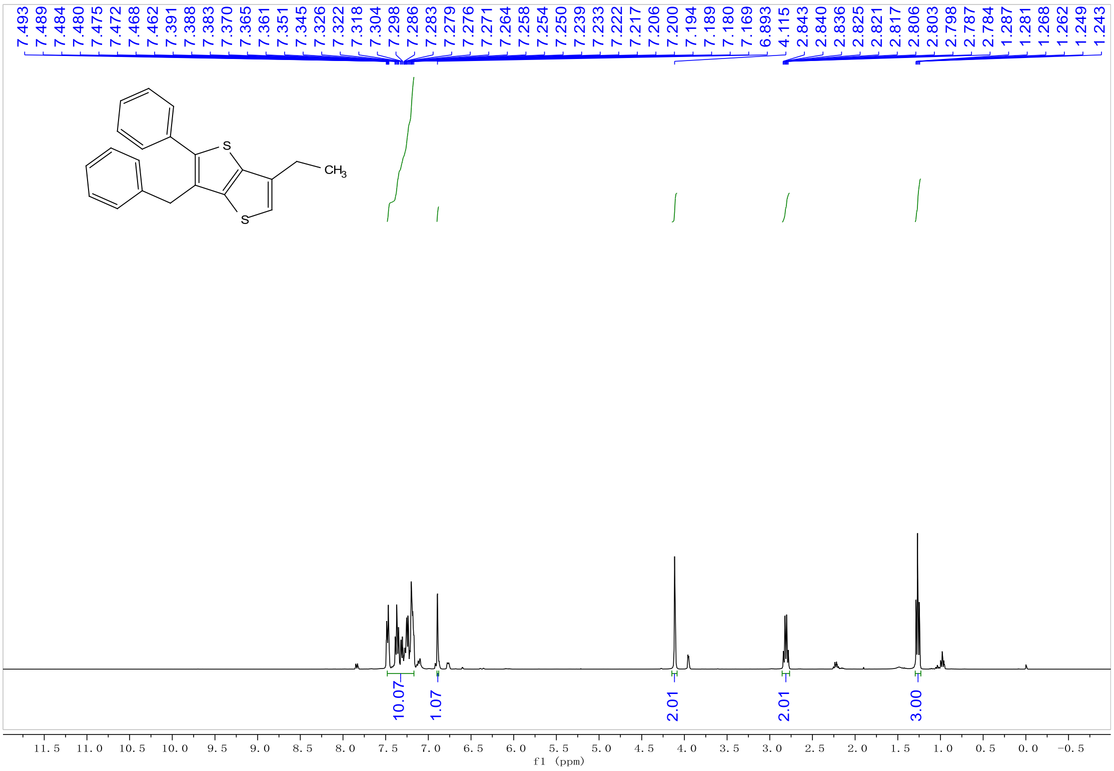

^{13}C NMR (125 MHz, $CDCl_3$) spectrum of compound 2s

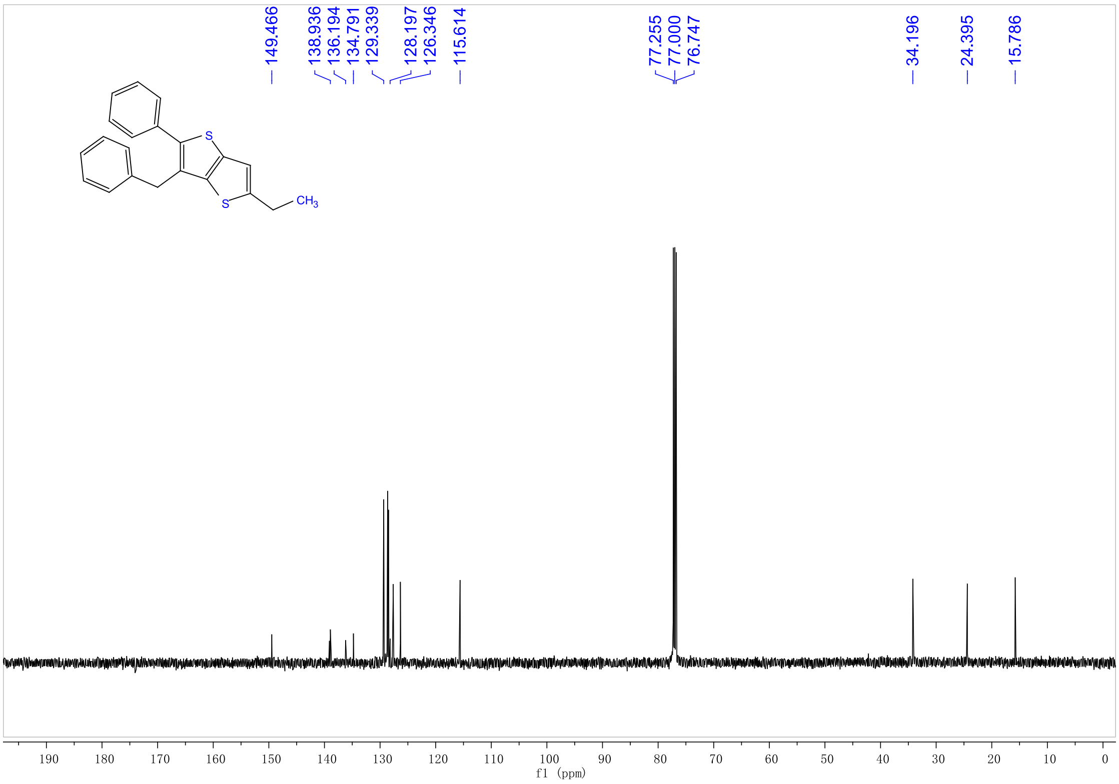

^{1}H NMR (500 MHz, $CDCl_3$) spectrum of compound 2t

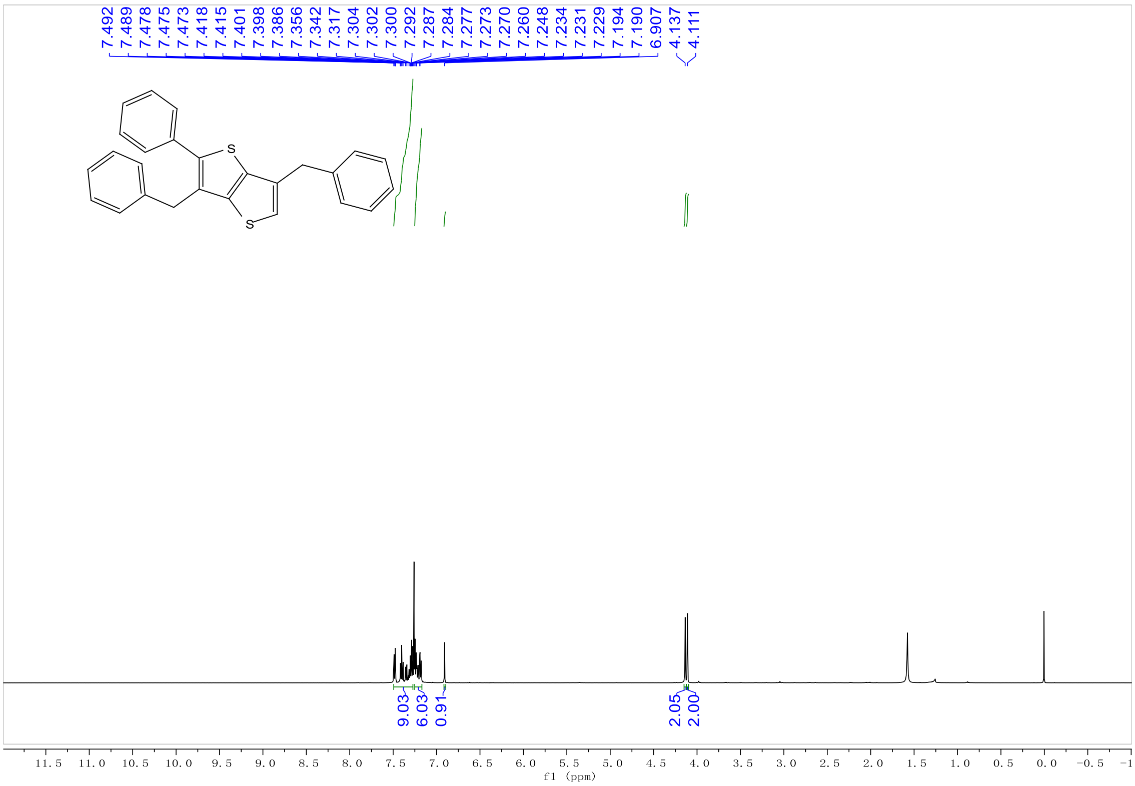

^{13}C NMR (125 MHz, $CDCl_3$) spectrum of compound 2t

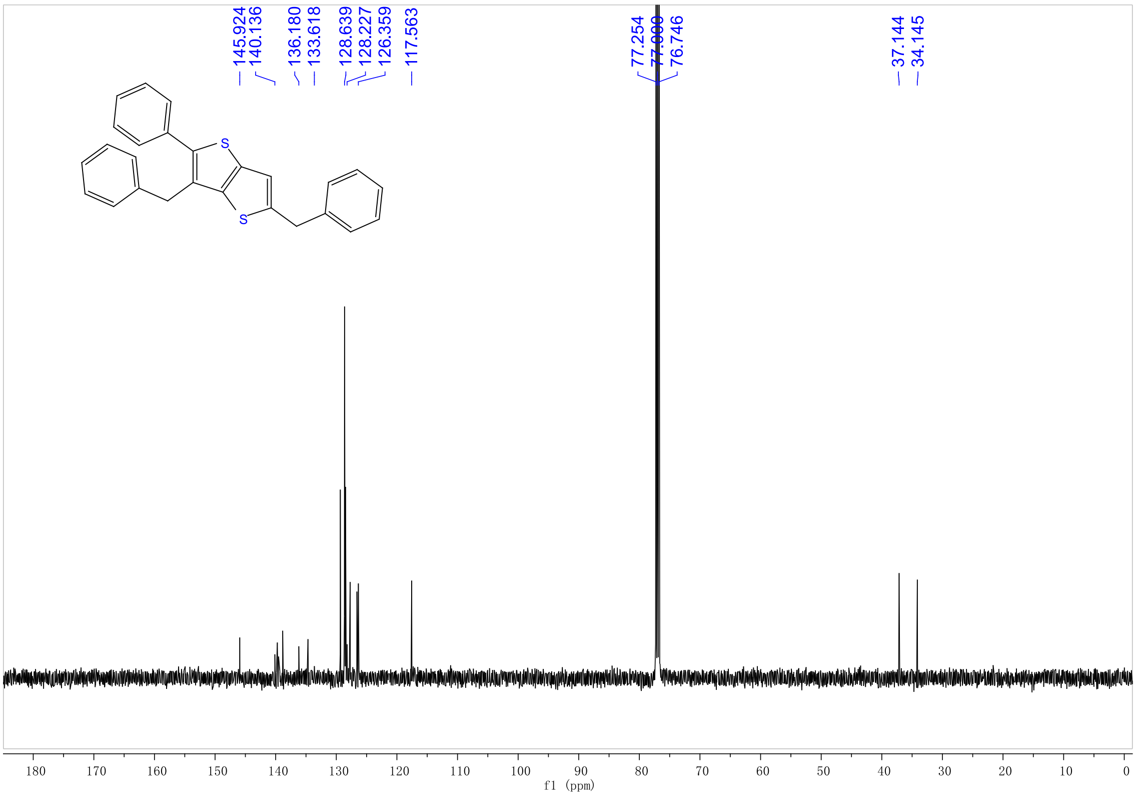

^1H NMR (400 MHz, $CDCl_3$) spectrum of compound 3a

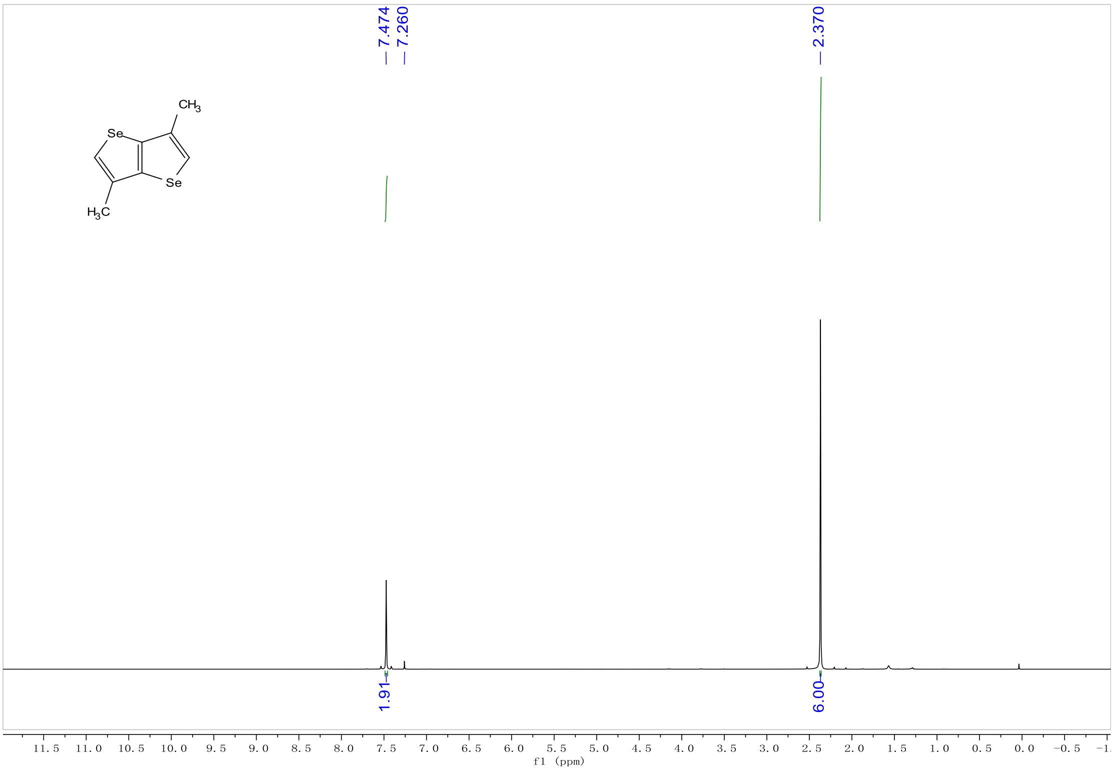

^{13}C NMR (100 MHz, $CDCl_3$) spectrum of compound 3a

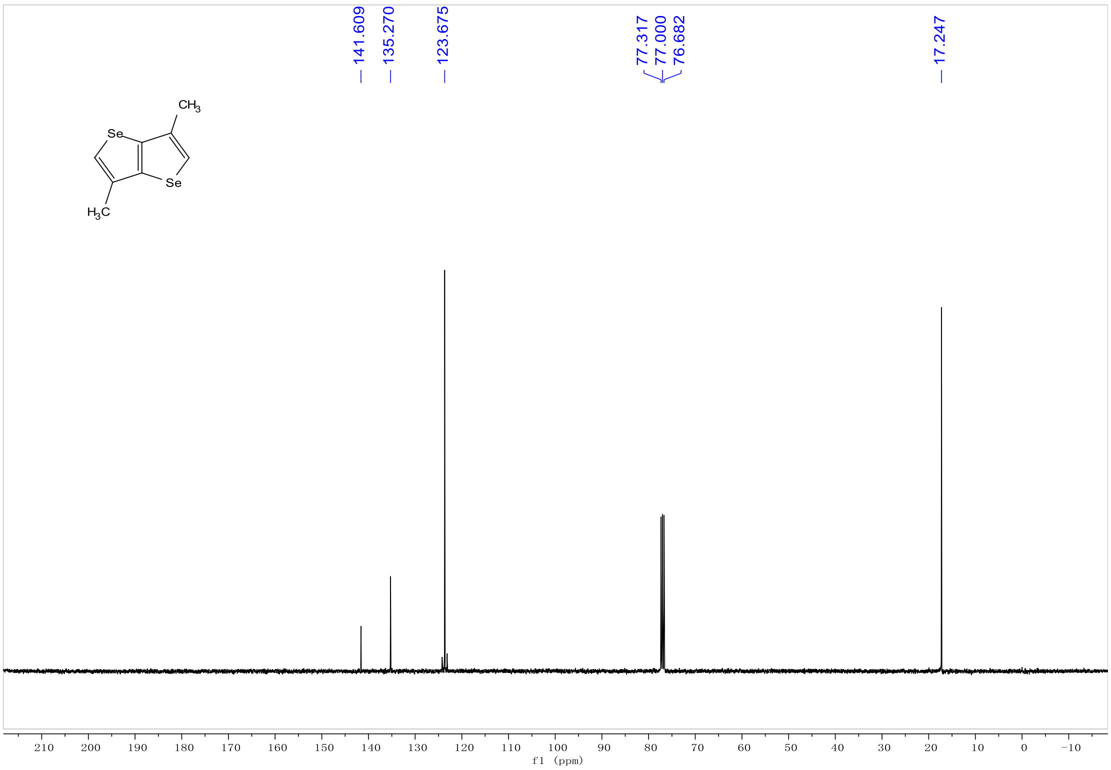

^{1}H NMR (400 MHz, $CDCl_3$) spectrum of compound 3b

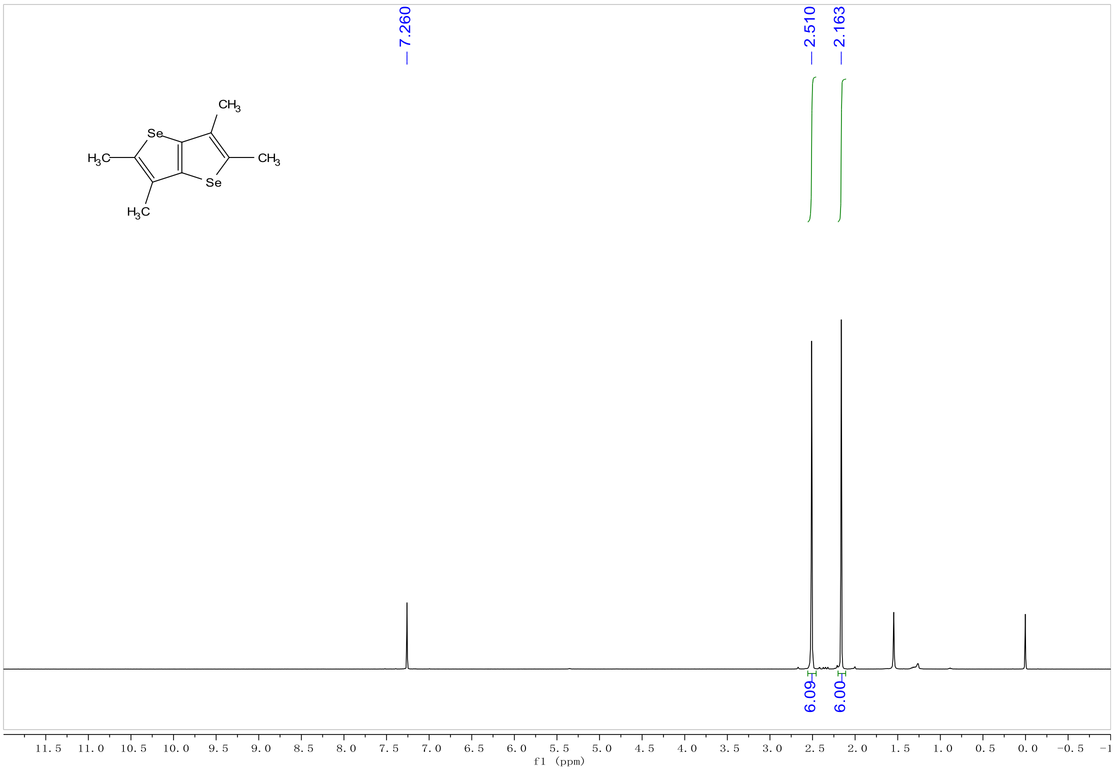

^{13}C NMR (100 MHz, $CDCl_3$) spectrum of compound 3b

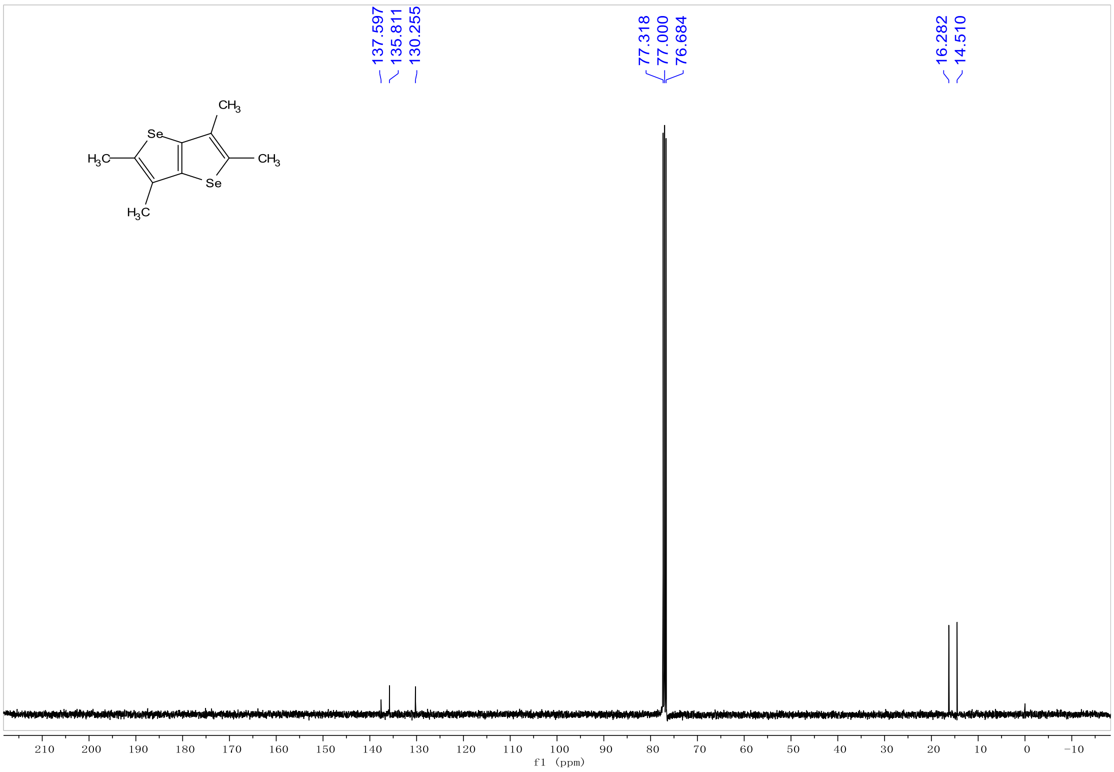

^{1}H NMR (400 MHz, $CDCl_3$) spectrum of compound 4a

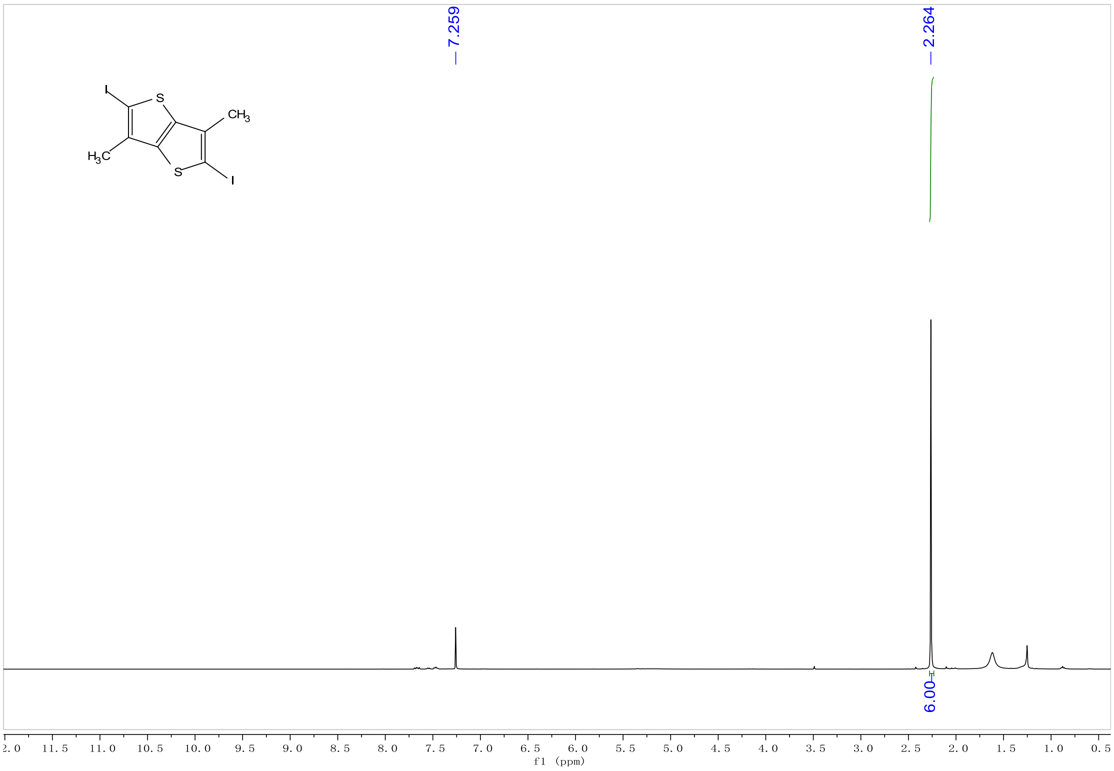

^{13}C NMR (100 MHz, $CDCl_3$) spectrum of compound 4a

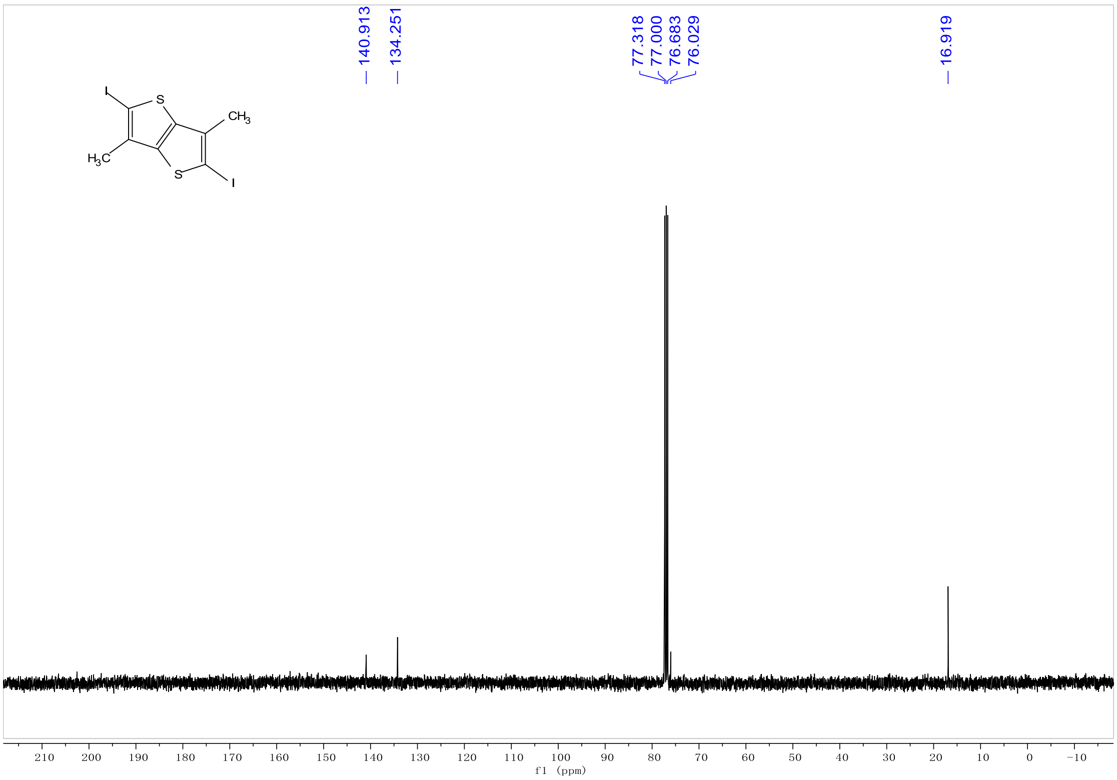

^{1}H NMR (400 MHz, $CDCl_3$) spectrum of compound 4b

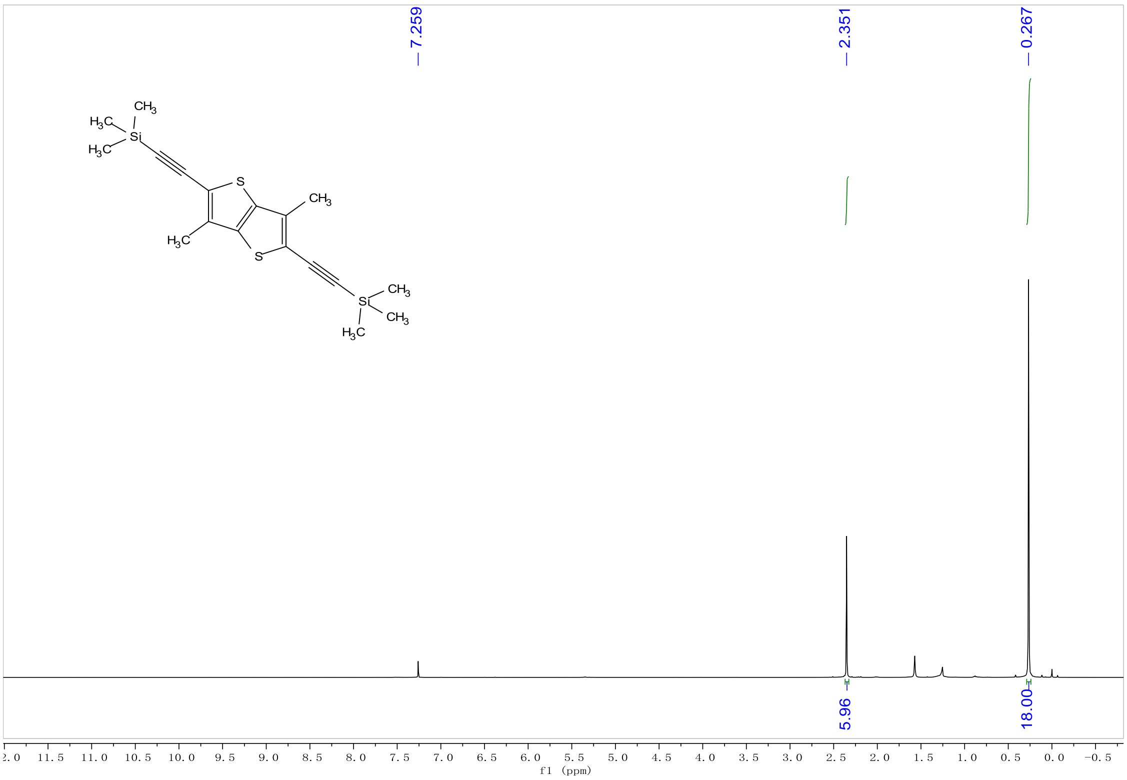

^{13}C NMR (100 MHz, $CDCl_3$) spectrum of compound 4b

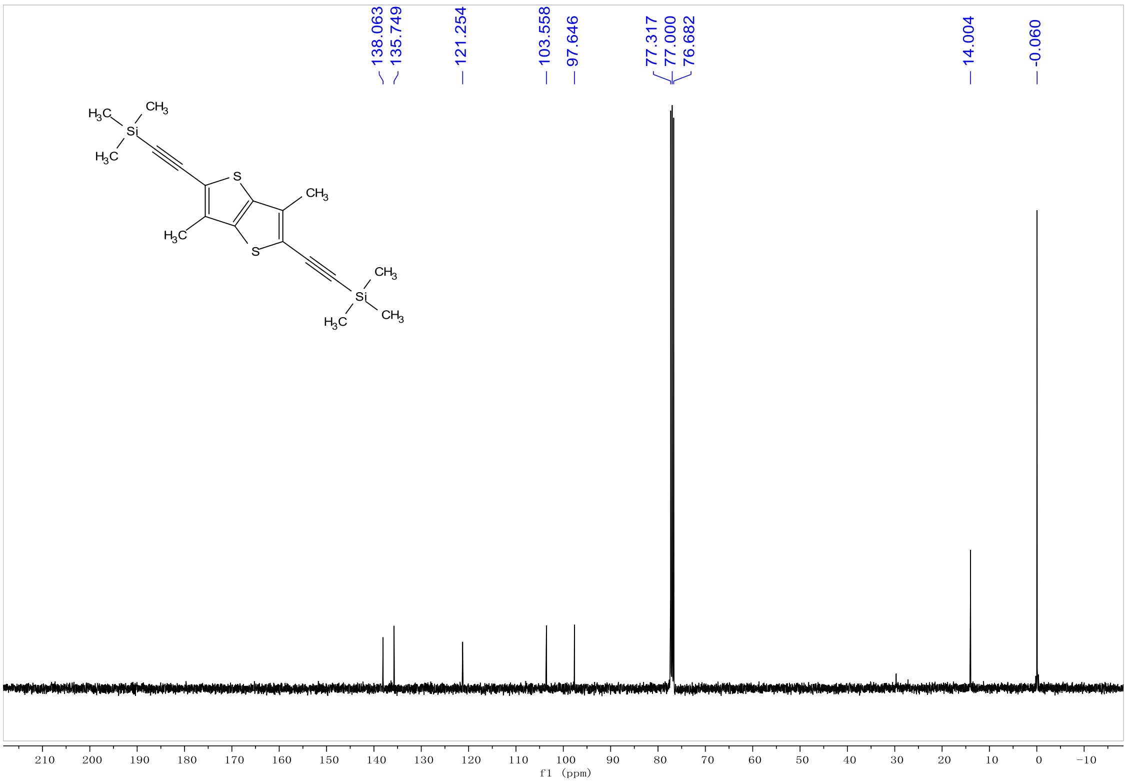

^{1}H NMR (400 MHz, $CDCl_3$) spectrum of compound 4c

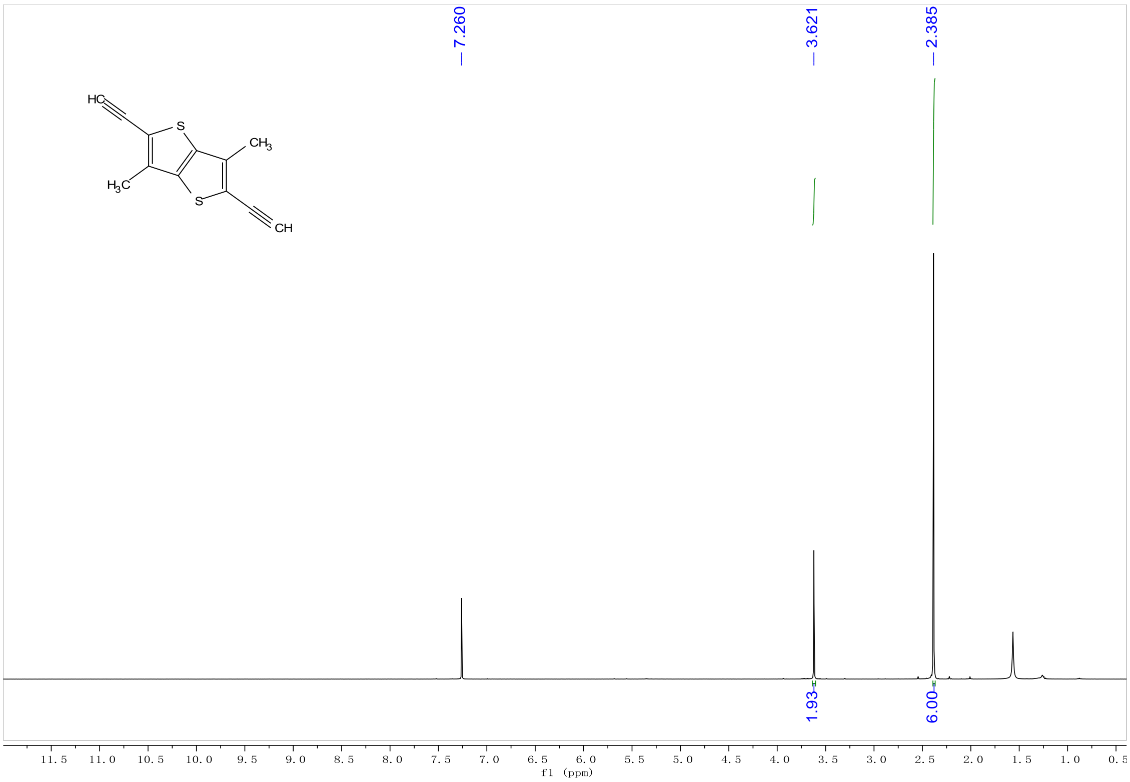

^{13}C NMR (100 MHz, $CDCl_3$) spectrum of compound 4c

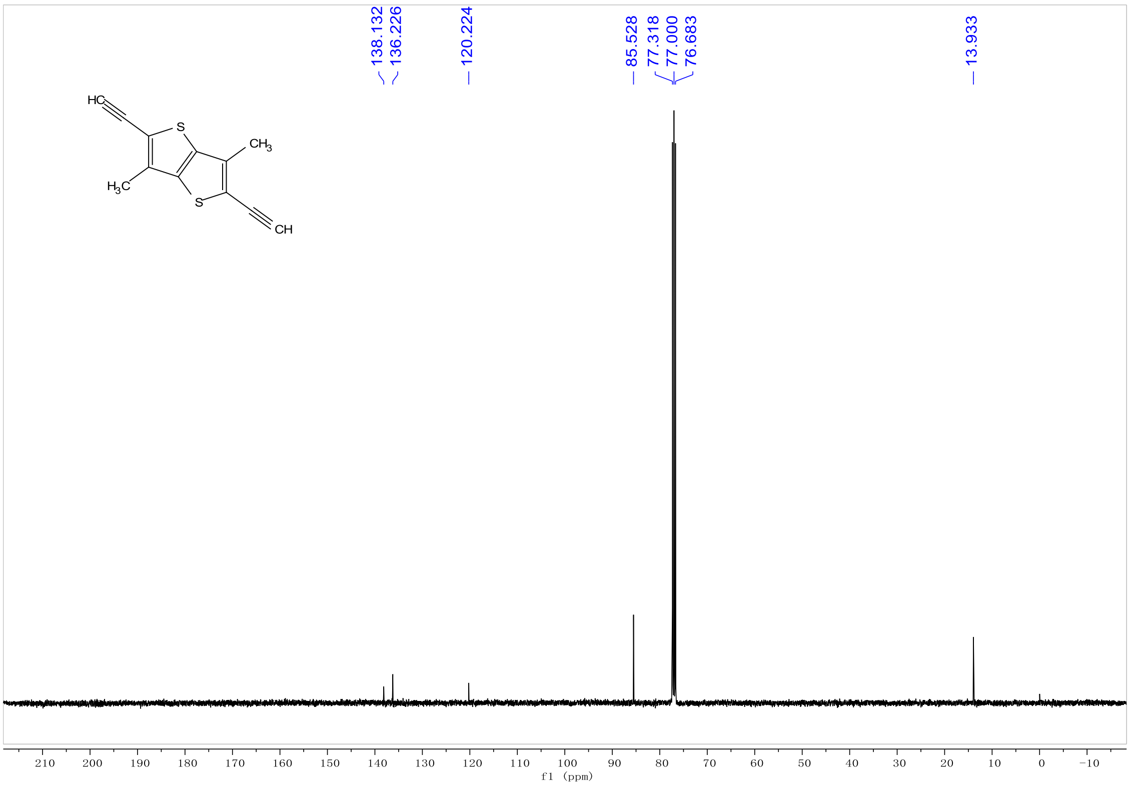

^{1}H NMR (400 MHz, $CDCl_3$) spectrum of compound 4d

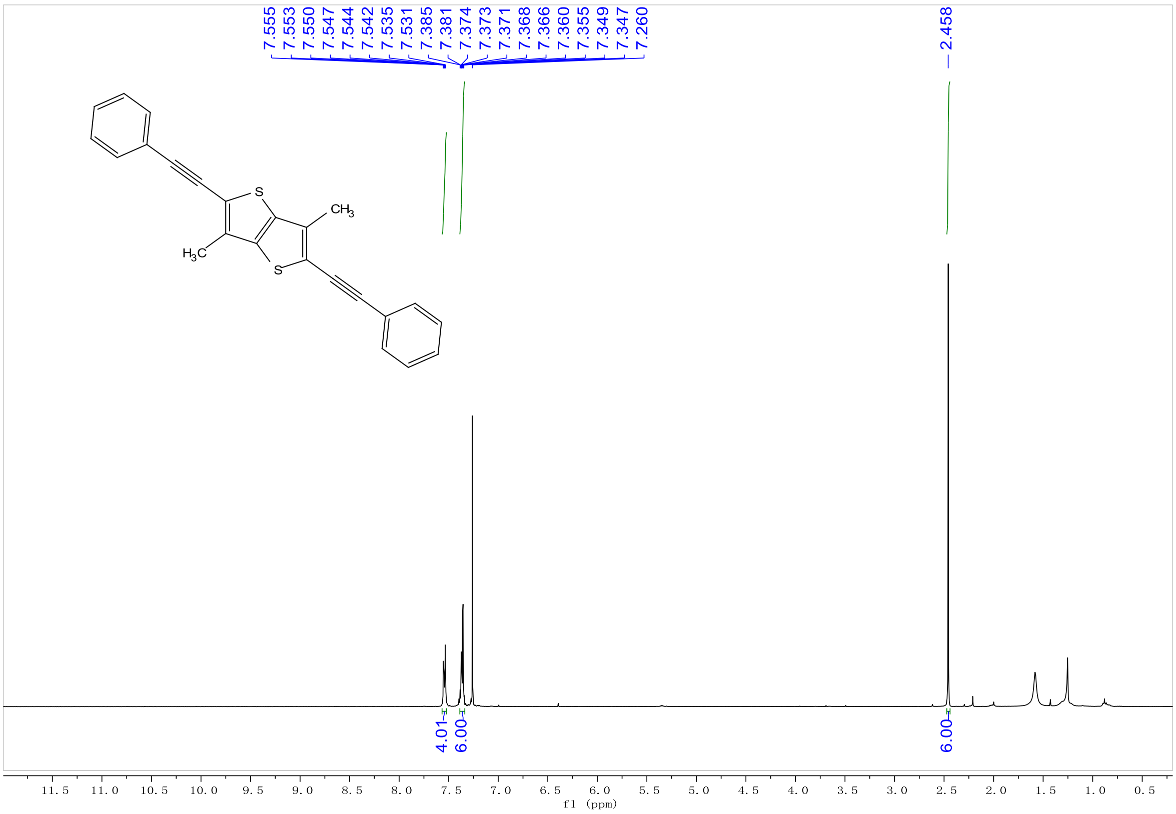

^{13}C NMR (100 MHz, $CDCl_3$) spectrum of compound 4d

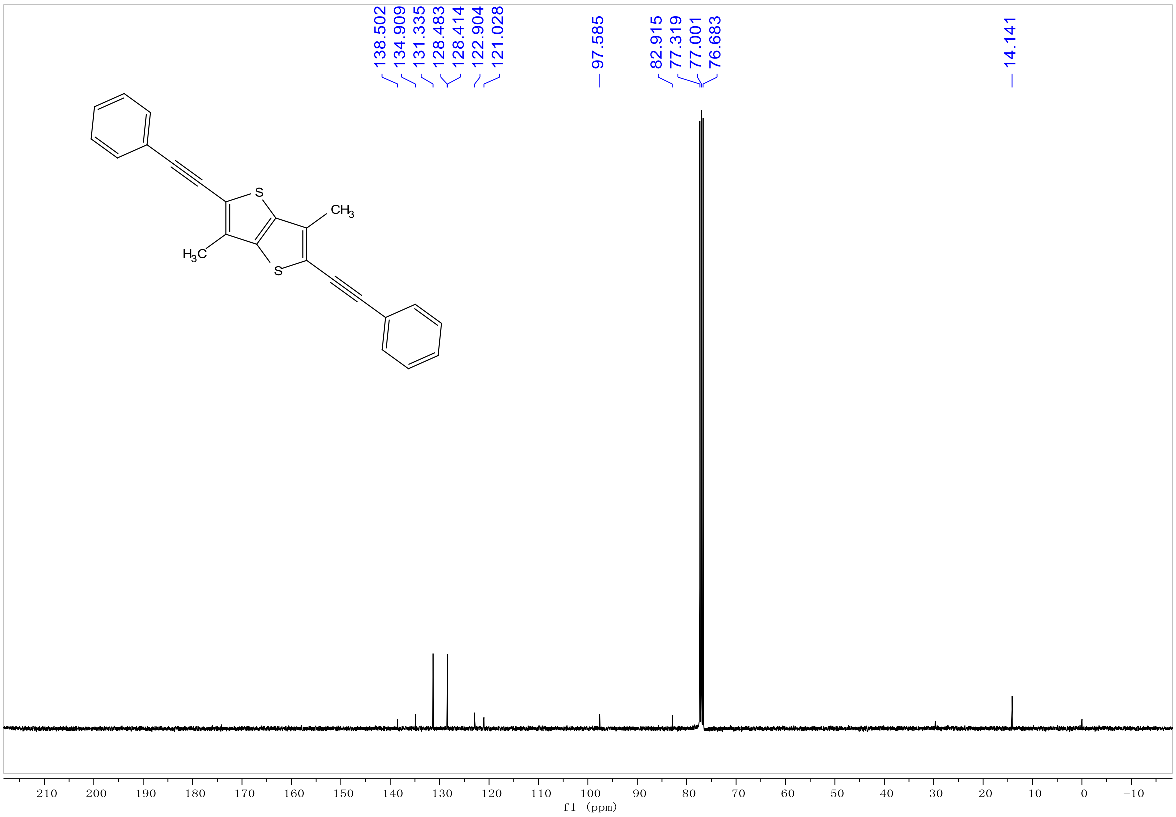

^{1}H NMR (400 MHz, $CDCl_3$) spectrum of compound 4e

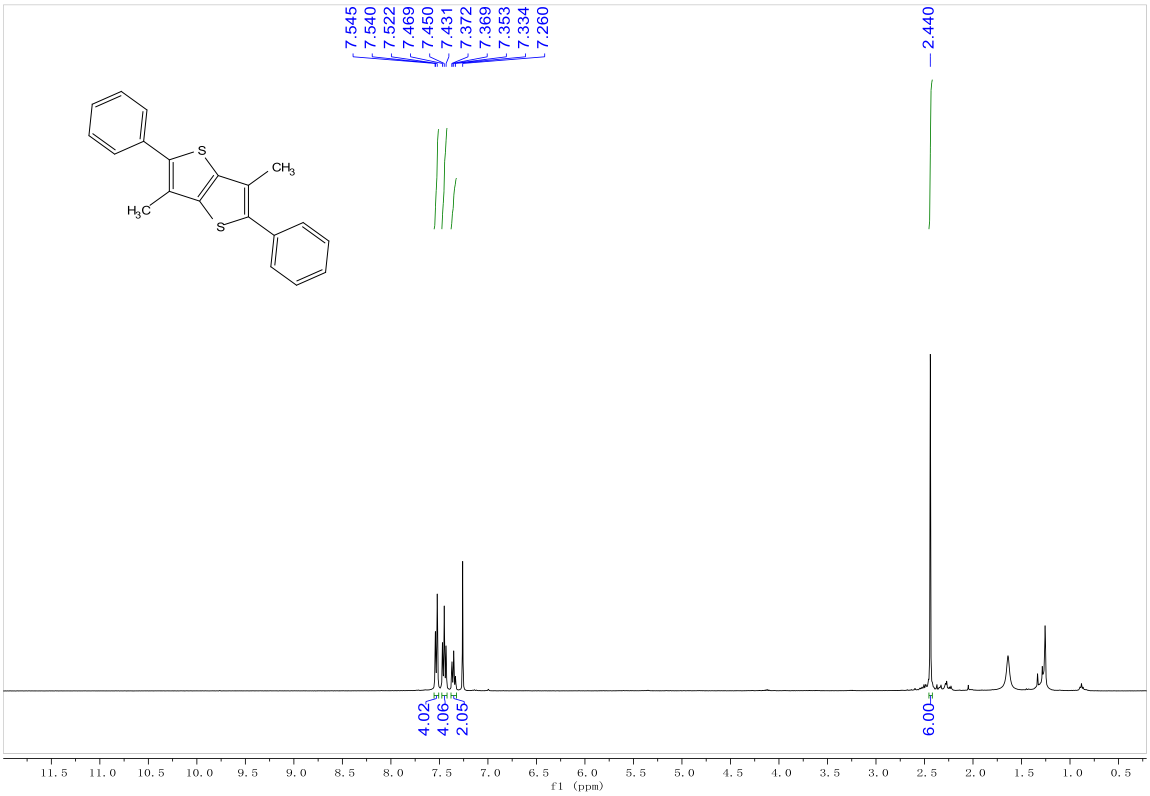

^{13}C NMR (100 MHz, $CDCl_3$) spectrum of compound 4e

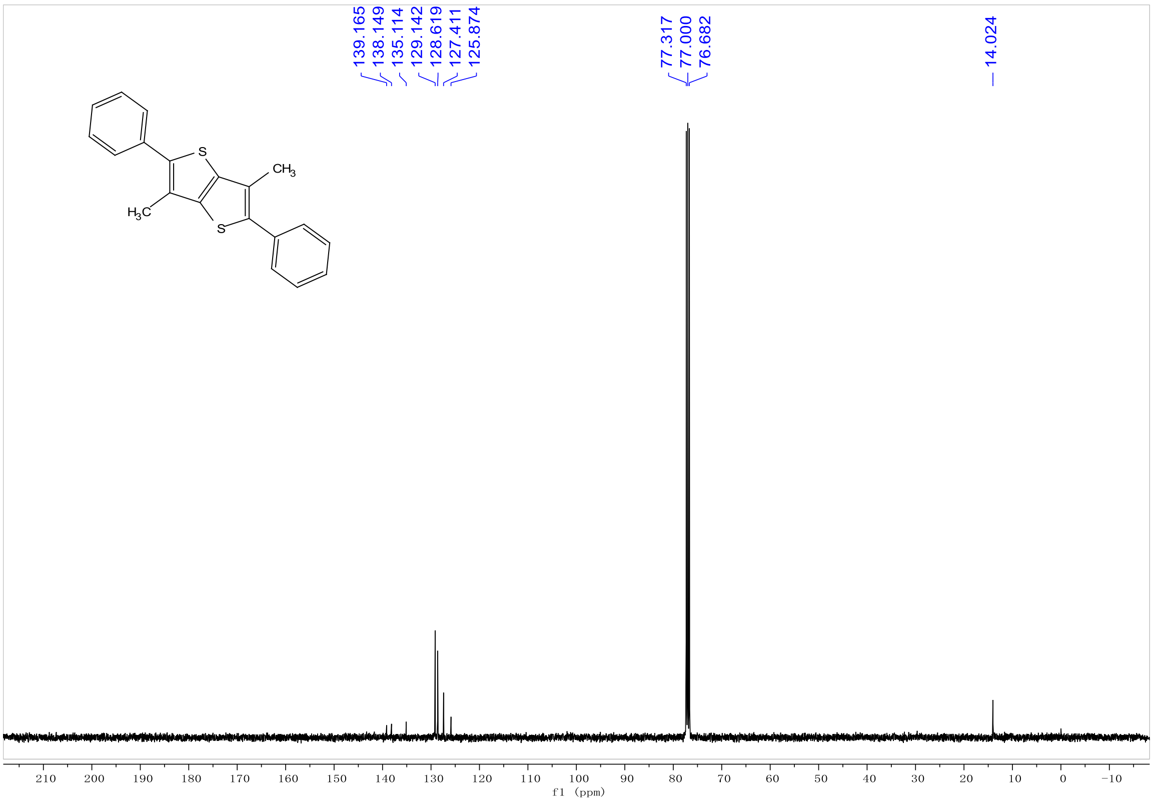

^{1}H NMR (400 MHz, $CDCl_3$) spectrum of compound 5b

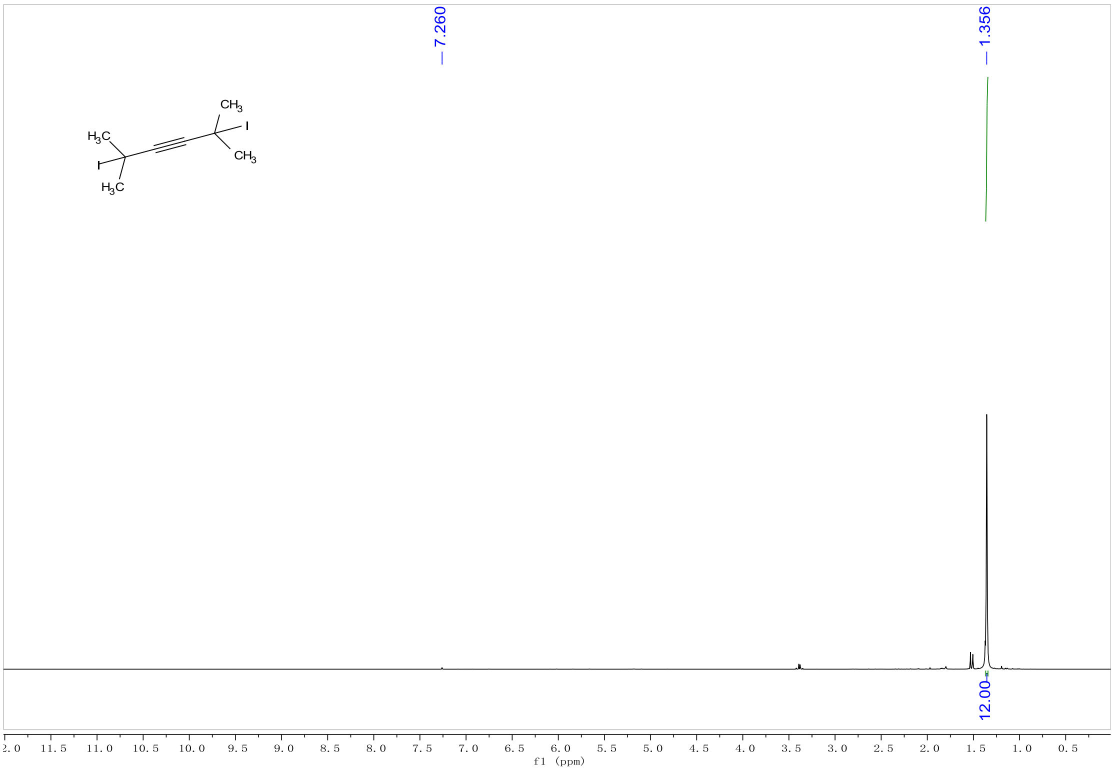

^{13}C NMR (100 MHz, $CDCl_3$) spectrum of compound 5b

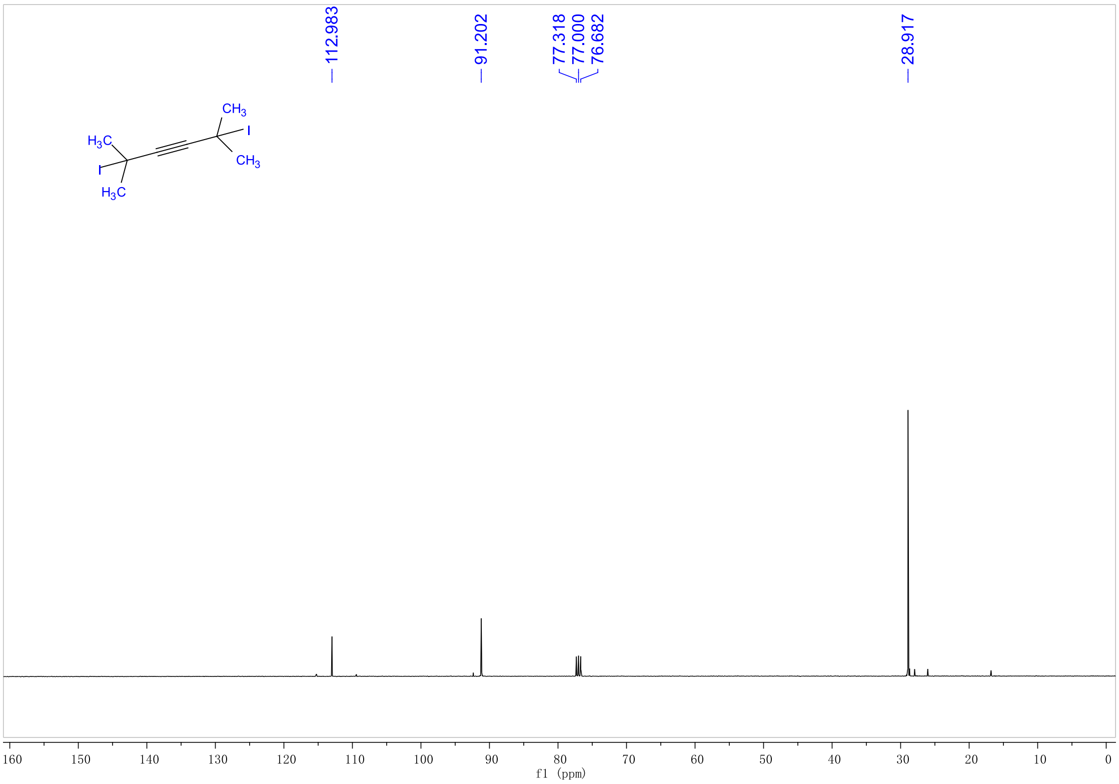

^{1}H NMR (400 MHz, $CDCl_3$) spectrum of compound 5d

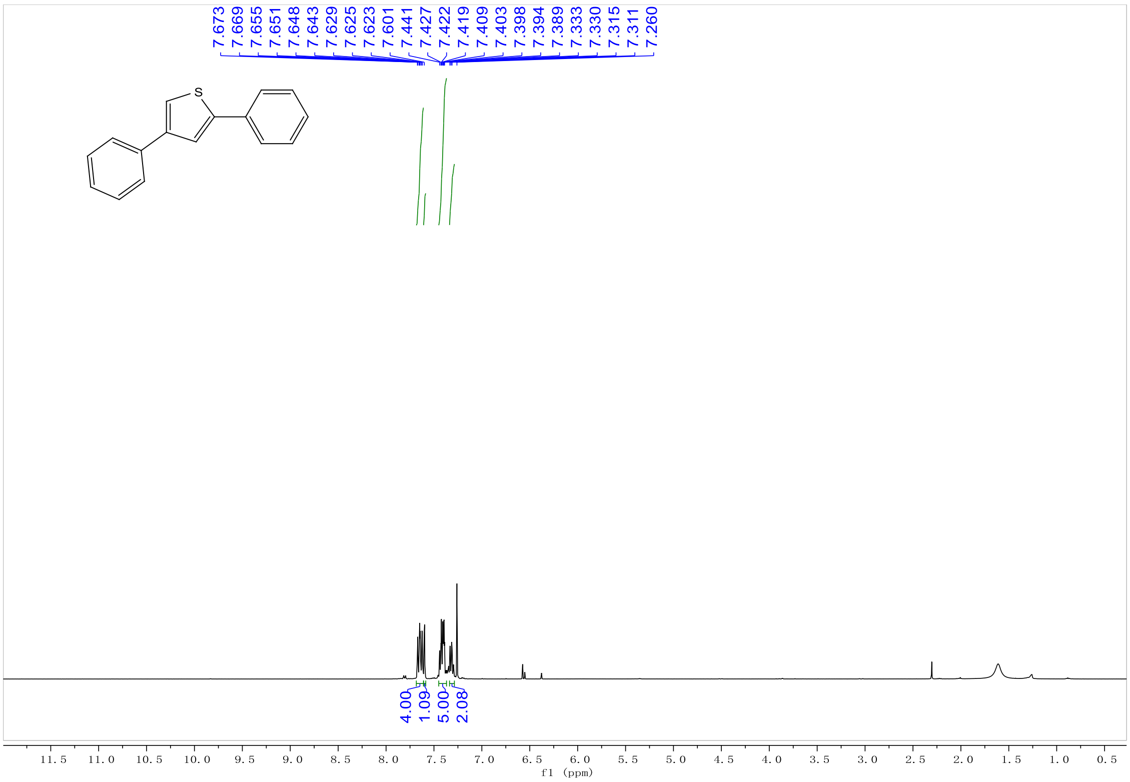

^{13}C NMR (100 MHz, $CDCl_3$) spectrum of compound 5d

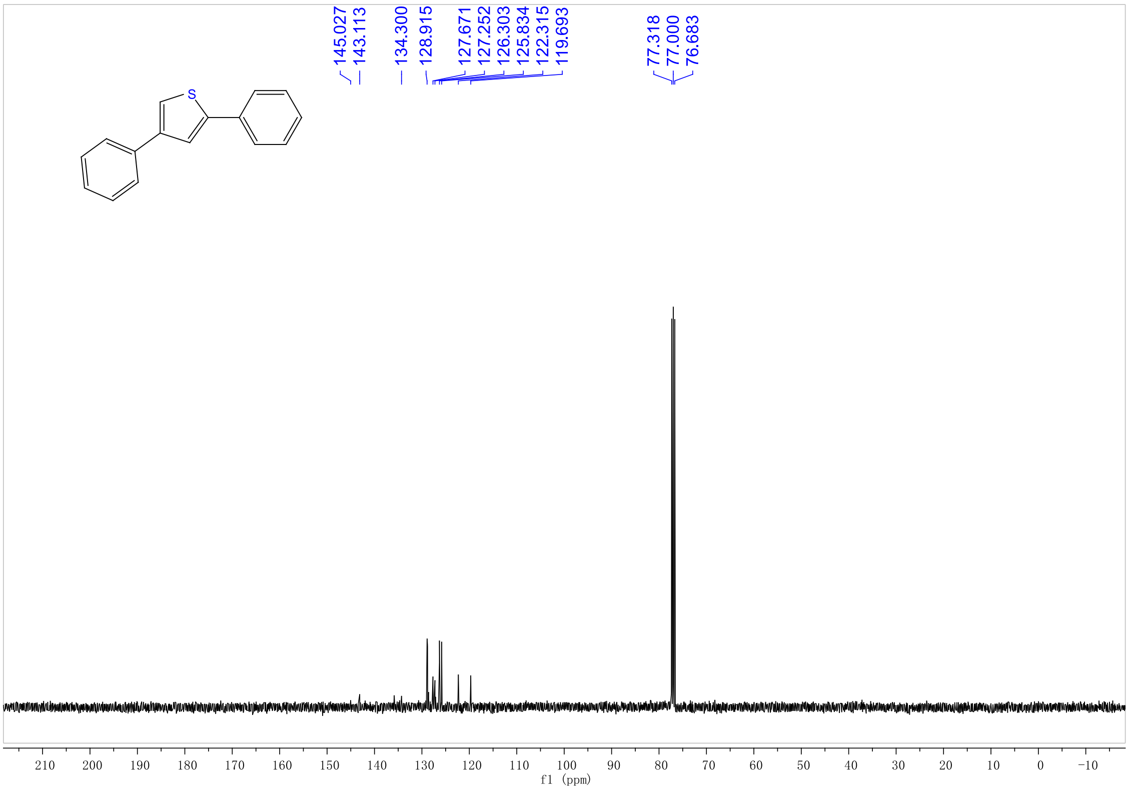

^{1}H NMR (400 MHz, $CDCl_3$) spectrum of compound 2a-D

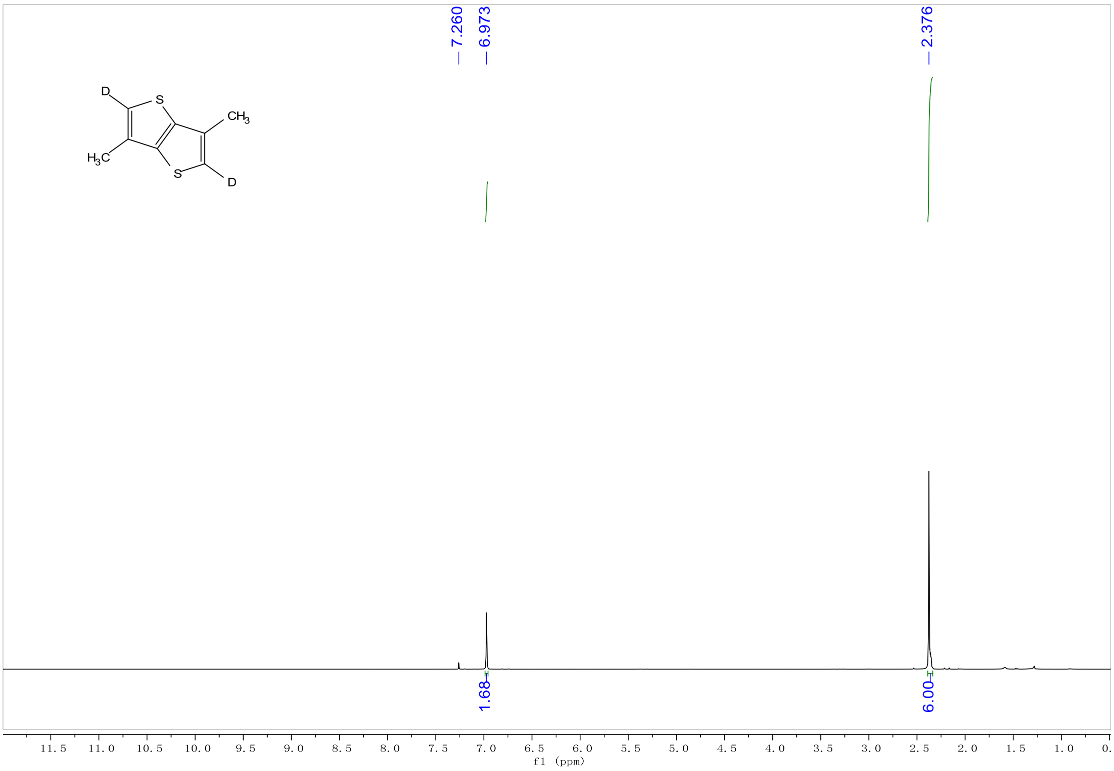

^{13}C NMR (100 MHz, $CDCl_3$) spectrum of compound 2a-D

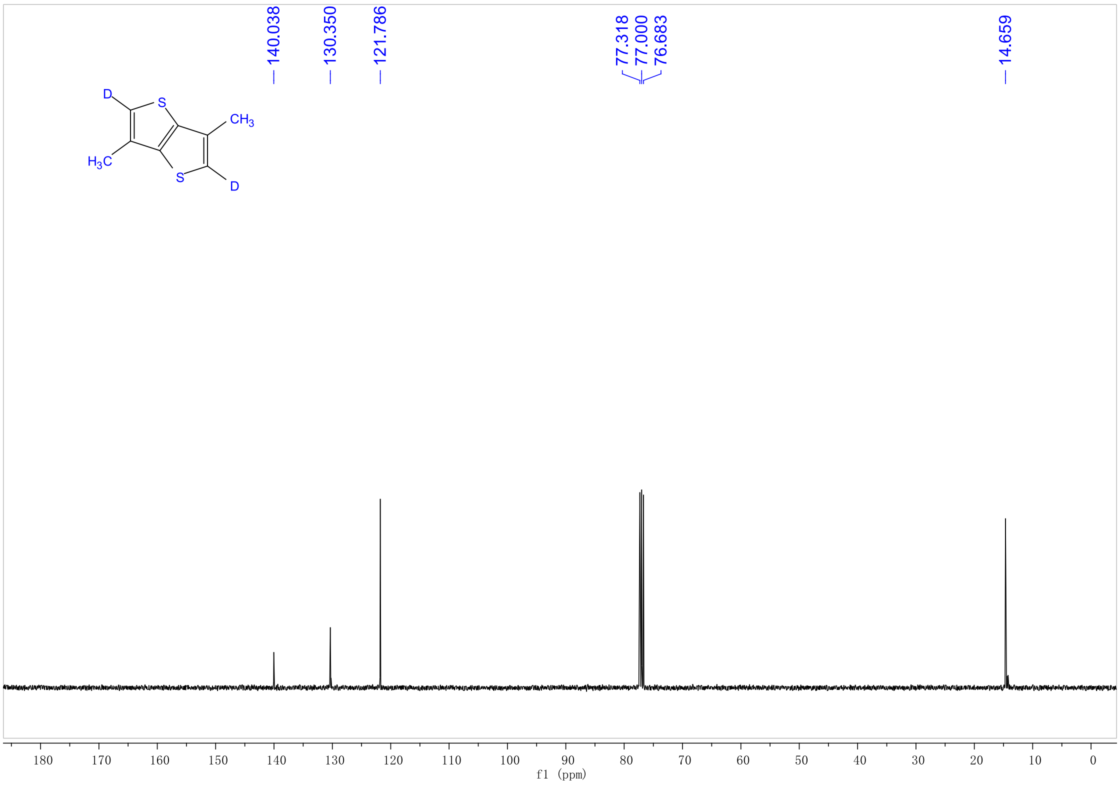

^{2}H NMR (77 MHz, CH_2Cl_2) spectrum of compound 2a-D

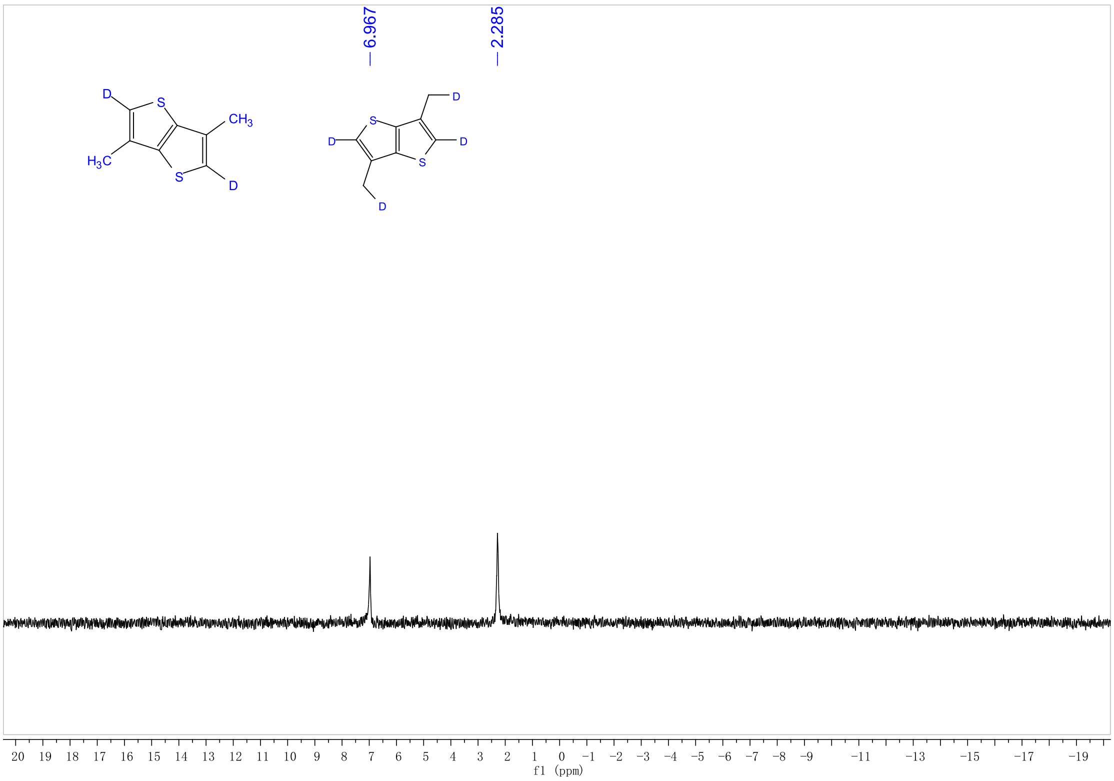

Supplement: Supplementary file 1 [file molecules-29-05507-s001.zip › molecules-3303427-supplementary.pdf]
